# Methyl isocyanide as a convertible functional group for the synthesis of spirocyclic oxindole γ-lactams via post-Ugi-4CR/transamidation/cyclization in a one-pot, three-step sequence

**DOI:** 10.3762/bjoc.14.74

**Published:** 2018-04-18

**Authors:** Amarendar Reddy Maddirala, Peter R Andreana

**Affiliations:** 1 Department of Chemistry and Biochemistry and School of Green Chemistry and Engineering, University of Toledo, 2801 W. Bancroft Street, Toledo, OH 43606, USA

**Keywords:** convertible isocyanides, lactams, molecular diversity, oxindoles, transamidation

## Abstract

The synthesis of spiro[indoline-3,2'-pyrrole]-2,5'(1'*H*)-diones and spiro[indoline-3,2'-pyrrolidine]-2,5'-diones, via a post-Ugi-domino transamidation/cyclization sequential process, has been achieved in three sequential steps utilizing a one-pot reaction protocol. The variation in carboxylic acid substrates allows for the generation of new chiral racemic quaternary carbon centers under basic conditions providing molecular diversity and a small library of spirocyclic oxindoles.

## Introduction

The Ugi-multicomponent coupling reaction [1–2], followed by post-modification transformations involving tandem reaction sequences [3] and the Ugi–deprotection–cyclization (UDC) strategies [4–8] have been exploited as powerful tools allowing access to biological and pharmaceutical high-value heterocyclic scaffolds [9–11]. These reactions are appealing in that they are atom economical, simple and generate ample molecular diversity with the ease of using readily available starting materials. Developing new, post-modified Ugi-four-component reaction (Ugi-4CR) transformations in domino cyclization [12–15] sequences are very important for achieving unprecedented chemical bonds and functionality towards the construction of synthetic scaffolds.

The synthesis of spirocyclic oxindoles has always been of key interest to organic chemists because of significant biological activity [16–20] and their presence in naturally occurring molecules [21–23]. Significant efforts have been made to design creative synthetic strategies for spirocyclic oxindole molecules, of which, isatin-based domino reactions [24–30] have proved to be very versatile [31] and readily achievable [32–37]. However, finding a simple and efficient synthetic method for these molecules that allows for structural diversity is also important but not necessarily trivial. For these and other reasons, we became interested in synthesizing spiro[indoline-3,2'-pyrrole]-2,5'(1'*H*)-dione and spiro[indoline-3,2'-pyrrolidine]-2,5'-dione scaffolds (a class of spirocyclic oxindole γ-lactams).

**Scheme 1:**
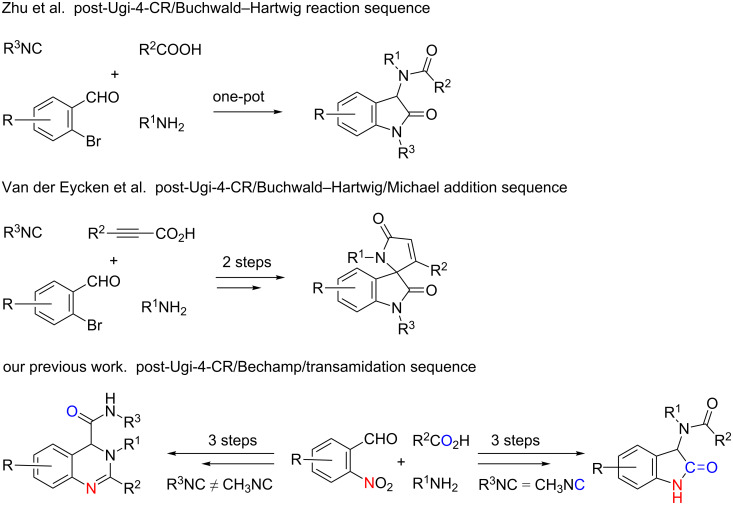
Previously reported post-Ugi-4CR methods for the synthesis of 2-oxindoles and spirocyclic 2-oxindoles.

There have been other groups in the past, including our own research group, who have reported on post-modified Ugi-four-component synthetic strategies (Scheme 1) towards the synthesis of 2-oxindoles and spiro[indoline-3,2'-pyrrole]-2,5'(1'*H*)-diones and spiro[indoline-3,2'-pyrrolidine]-2,5'-diones. Zhu et al. [38] reported 3-substituted-2-indolinones via a microwave-assisted post-Ugi-4CR/Buchwald–Hartwig reaction and another similar approach was illustrated by Van der Eycken et al. [39] for spiro[indoline-3,2'-pyrrole]-2,5'(1'*H*)-diones. In previous efforts to study 3-substituted 2-indolinones through a three-step post-Ugi-4CR/Bechamp type-reduction followed by a transamidation sequence strategy [40], we came across interesting observations. We noted that when methyl isocyanide [41–42] was used for the Ugi-4CR and the subsequent post-intramolecular transamidation was performed under acidic conditions, particularly in the presence of TFA, the reaction led to 3-substituted 2-indolinones in a three-step process [40]. In this work, we discovered that methyl isocyanide [43] operates under a mechanism of convertible isocyanides (CICs) [44–49], and could be thought of as a synthetic equivalent to ‘CO’ for insertion into the 2-indolinone backbone (shown in Scheme 1). To further elaborate on this observation and for understanding the role of methyl isocyanide as a CIC, we designed an efficient synthetic strategy for spiro[indoline-3,2'-pyrrole]-2,5'(1'*H*)-diones and spiro[indoline-3,2'-pyrrolidine]-2,5'-diones via a one-pot, three-step reaction sequence. Advantages of this strategy include: a) minimal number of synthetic steps, b) avoidance of tedious work-up procedures including purification, and c) use of starting materials either readily available or facile to synthesize.

**Scheme 2:**
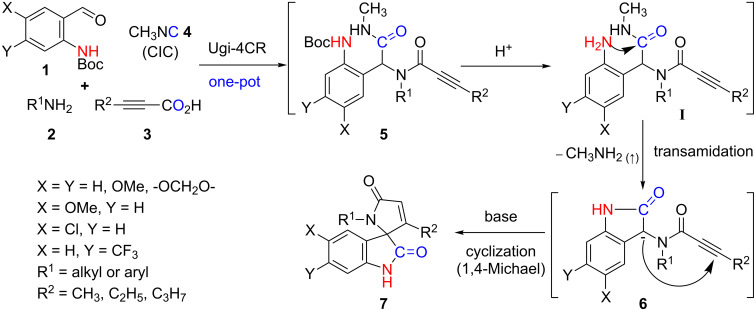
Post-Ugi-4CR/transamidation/cyclization sequence.

Here within, we document that the reaction sequence for spirocyclic oxindole γ-lactams (Scheme 2) follows a three-step sequential strategy involving: a) an Ugi-4CR, b) an acid-promoted intramolecular transamidation, and c) a base-mediated cyclization giving spiro[indoline-3,2'-pyrrole]-2,5'(1'*H*)-diones. We propose that the acid-mediated Boc deprotection in the Ugi intermediate **5** leads to aniline intermediate **I** which can simultaneously undergo a CIC cyclization through an intramolecular transamidation process giving compound **6** and, in the process, extrude methylamine as a gaseous byproduct. Furthermore, compound **6** is proposed to undergo an intramolecular cyclization, under basic conditions, yielding target compounds **7** (Scheme 2).

## Results and Discussion

**Scheme 3:**
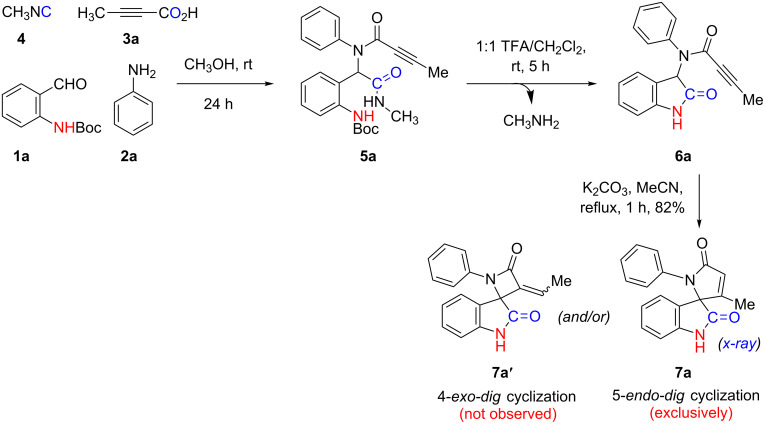
Base-promoted intramolecular 5-*endo-dig* cyclization.

**Figure 1:**
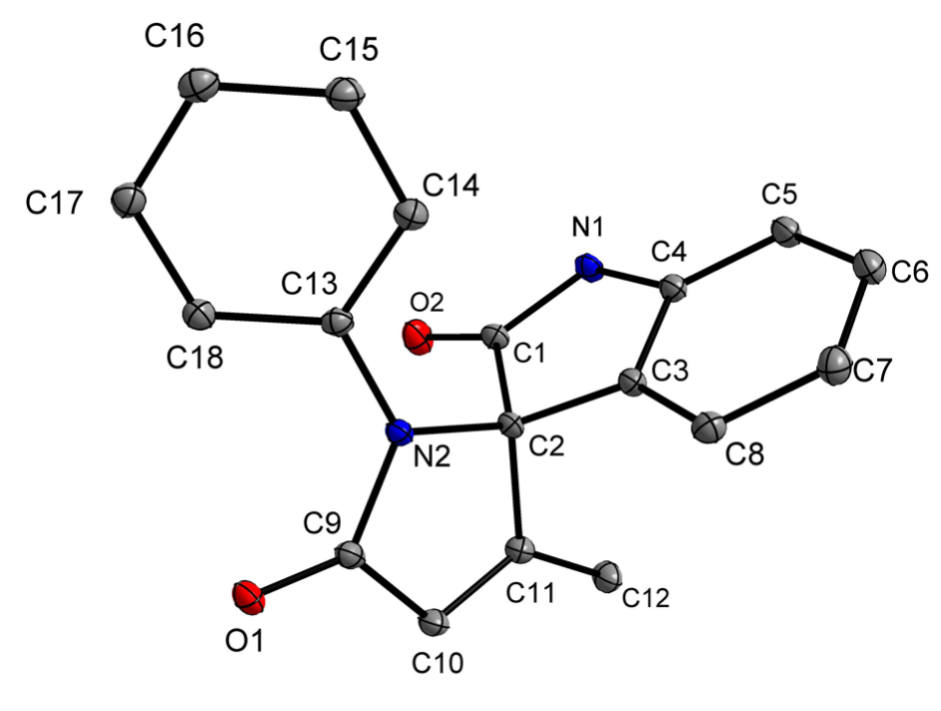
ORTEP diagram of compound **7a**.

Our initial synthetic attempts began with the Ugi-4CR involving stoichiometrically equal amounts of 2-(Boc-amino)benzaldehyde (**1a**), aniline (**2a**), tetrolic acid (**3a**), and methyl isocyanide (**4**) in methanol at room temperature to generate adduct **5a**; confirmed using mass spectroscopic analysis (Scheme 3). Intermediate **5a**, which was not purified, was prone to undergo an intramolecular transamidation when 50% TFA was used to remove the Boc group in DCM at room temperature for 5 h ultimately yielding **6a** and methylamine as the gaseous byproduct. Following the neutralization of TFA, unpurified compound **6a** was tested for the ability to cyclize under basic conditions. We began our cyclization studies with **6a** by dissolving it in acetonitrile following by the addition of 2 equiv K_2_CO_3_ and then refluxing for 1 h. The reaction was monitored by TLC, which indicated the disappearance of the starting material and formation of a new spot. Post-work-up and purification, spectroscopic analysis of the product matched nicely with the 5-*endo-dig* cyclization product **7a** and not the 4-*exo-dig*-cyclization compound **7a'** (Scheme 3). We rationalized, as per Baldwin’s rules, that the 5-*endo-dig* cyclization of a 1,4-Michael addition was more favorable than the 4-*exo-dig*-cyclization [50–51]. The structure of compound **7a** was definitively confirmed by X-ray analysis (Figure 1).

We then elected to carry out yield optimization studies of the cyclization reaction using a number of solvents, reagents and reaction temperatures (Table 1) and also to explore the possibility of a possible 4*-exo-dig* outcome. The study validated that all the reaction conditions, with the exception of AgOTf/DCM (Table 1, entry 6), led to the formation of the desired 5-*endo*-*dig* cyclization product **7a** and none of the 4-*exo-dig* cyclization product **7a'** was observed. In Table 1, entries 2–5 and 7 show moderate product yields whereas entries 1 and 8 show superior yields of >80%.

**Table 1:** Optimization conditions for the intramolecular cyclization (Scheme 3, **6**→**7a** and **7a'**).^a^

entry	reagent	solvent	temperature	time	yield **7a** (%)	yield **7a'** (%)

**1**	**K_2_CO_3_**	**MeCN**	**reflux**	**1 h**	**82**	**n.o.**
2	K_2_CO_3_	methanol	rt	2 h	72	n.o.
3	K_2_CO_3_	toluene	reflux	2 h	70	n.o.
4	Cs_2_CO_3_	toluene	reflux	2 h	72	n.o.
5	Et_3_N	DCM	rt	4 h	65	n.o.
6	AgOTf	DCM	rt	24 h	n.r.	n.r.
7	KO*t-*Bu	THF	rt	30 min	75	n.o.
**8**	**KO*t-*Bu**	**MeCN**	**rt**	**30 min**	**80**	**n.o.**

^a^n.o.: not observed, n.r.: no reaction; highlighted entries denote best results.

**Figure 2:**
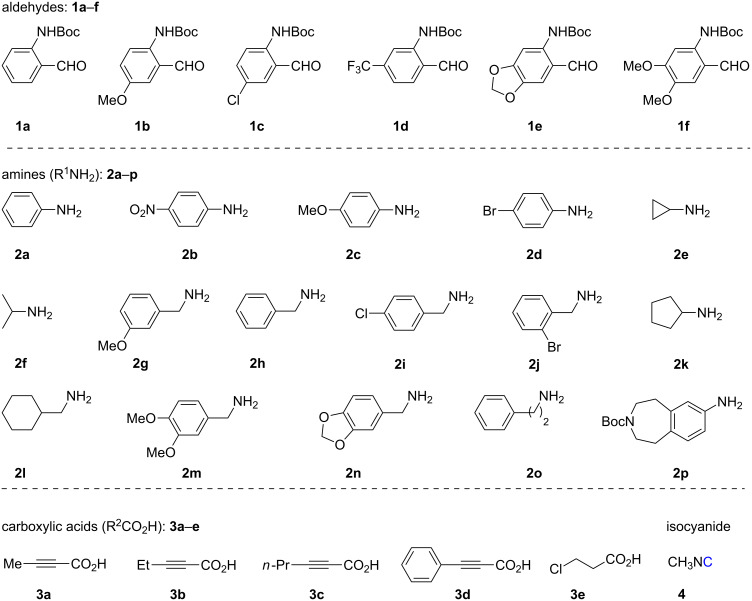
Readily and synthetically accessible starting materials.

**Scheme 4:**
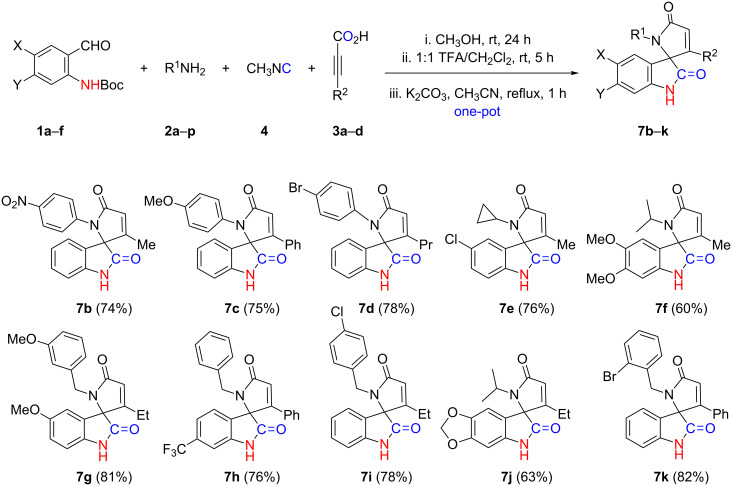
Reaction scope with varying combinations of substrates.

With the optimized reaction conditions in hand (Table 1, entries 1 and 8), the substrate scope was explored using a one-pot Ugi-4CR/transamidation/cyclization sequence employing various combinations of readily available and synthetically accessible starting materials (Figure 2). Under all circumstances, the intramolecular cyclization proceeded smoothly using K_2_CO_3_/MeCN reflux conditions and products **7b**–**k** were obtained in good to excellent yields (Scheme 4). Although the results shown only reflect the use of K_2_CO_3_/MeCN reflux conditions, similar, if not exact reaction outcomes were observed when conditions from Table 1, entry 8 were used.

**Scheme 5:**
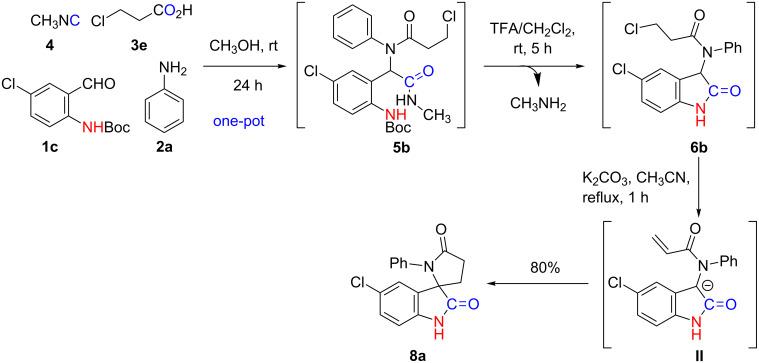
Synthesis of 5-chloro-1'-phenylspiro[indoline-3,2'-pyrrolidine]-2,5'-dione (**8a**).

To further explore the utility of our methodology, we examined similar reaction conditions for the synthesis of spiro[indoline-3,2'-pyrrolidine]-2,5'-dione scaffolds (Scheme 5). For this reaction we used 2-(Boc-amino)benzaldehyde **1c**, aniline (**2a**), 3-chloropropanoic acid (**3e**) and methyl isocyanide (**4**) in a one-pot reaction process to generate compound **6b** from **5b**. In all cases, the Michael acceptor [52] (intermediate **II**) was generated in situ from **6b**, under basic conditions (Table 1, entry 1, K_2_CO_3_/MeCN/reflux), followed by the intramolecular cyclization proceeding through a 1,4-Michael addition to form an exclusive 5*-endo-trig* cyclization of 5-chloro-1'-phenylspiro[indoline-3,2'-pyrrolidine]-2,5'-dione (**8a**, Scheme 5). Compound **8a** was unequivocally confirmed by both, mass spectral analysis and NMR.

**Figure 3:**
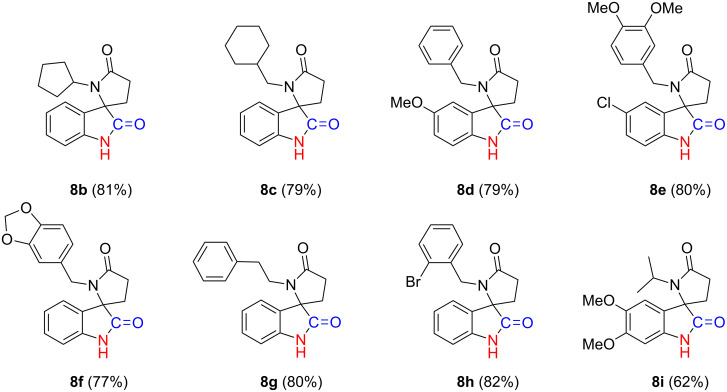
Small molecule library of spiro[indoline-3,2'-pyrrolidine]-2,5'-dione analogs.

**Scheme 6:**
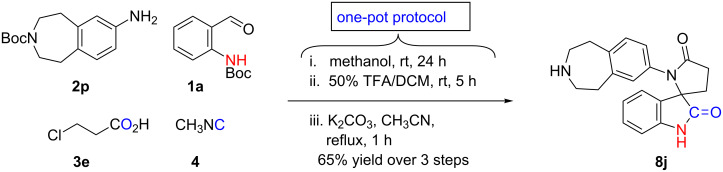
Method applicability for the one-pot synthesis of 5-HT6 receptor antagonist **8j** [53].

Encouraged by the results, we prepared a library of spiro[indoline-3,2'-pyrrolidine]-2,5'-diones **8b**–**i** from readily available starting materials in which overall yields were determined to be moderate to good (Figure 3). Furthermore, the applicability and utility of this process was demonstrated through the synthesis of a 5-HT6 receptor antagonist **8j** (Scheme 6) [53].

## Conclusion

In conclusion, we have investigated and developed an efficient process towards spirocyclic α,β-unsaturated γ-lactam oxindoles and spirocyclic γ-lactams using a one-pot three-step post-Ugi-4CR intramolecular transamidation/cyclization approach. We utilized this strategy for the synthesis of a small library of spiro[indoline-3,2'-pyrrole]-2,5'(1'*H*)-dione and spiro[indoline-3,2'-pyrrolidine]-2,5'-dione analogs illustrating important utility toward biologically relevant compounds. To this point, our strategy was further extended and applied towards the synthesis of a well-known 5-HT6 receptor antagonist **8j**. We also successfully utilized methyl isocyanide, as a CIC, for spiro[indoline-3,2'-pyrrole]-2,5'(1'*H*)-diones and spiro[indoline-3,2'-pyrrolidine]-2,5'-diones. Immediate plans are underway to test the inhibitory properties of the newly synthesized compounds in in vitro assays.

## Experimental

All reagents and solvents that were purchased were used without further purification unless otherwise stated. Reaction progress was monitored by thin-layer chromatography (TLC). Spots on TLC were visualized using UV light. Column chromatography was performed using normal-phase silica gel. Yields refer to chromatographically and spectroscopically pure compounds. ^1^H and ^13^C NMR were recorded using a Bruker Avance III 600 MHz. The residual DMSO-*d*_6_^1^H quintet at δ 2.50 ppm and residual ^13^C septet at δ 39.51 ppm, CDCl_3_^1^H singlet at δ 7.27 ppm and ^13^C triplet at δ 77.23 ppm were used as standards for ^1^H NMR and ^13^C NMR spectra, respectively. Signal patterns are indicated as s: singlet; d: doublet; t: triplet; q: quartet; m: multiplet; dd: doublet of doublets; and br: broad. Coupling constants are reported in hertz (Hz). High resolution mass spectra (HRMS) were obtained using a Bruker Maxis 4G mass spectrometer.

**General procedure for 7a**–**k:** Into a clear solution of 2-(Boc-amino)benzaldehyde **1** (1 mmol) in methanol (5 mL) was added amine **2** (1 mmol) and stirred for 5 minutes at room temperature. Carboxylic acid **3** (1 mmol) and methyl isocyanide (**4**, 1 mmol) were then added simultaneously. The mixture was stirred until no noticeable amounts of starting material were visible by TLC. Upon completion of the reaction, methanol was evaporated under reduced pressure and the crude Ugi products, without any purification, dissolved in a CH_2_Cl_2_ and trifluoroacetic acid mixture (1:1, 2 mL) and subsequently stirred at room temperature for 5 h. The reaction progress was monitored by TLC and upon completion of the reaction the solvent was evaporated under reduced pressure. Without any purification, the crude compound was dissolved in acetonitrile (2 mL) and K_2_CO_3_ (2 mmol) was added. The reaction was allowed to stir under refluxing conditions for 1 h and the reaction was monitored for completion using TLC. Upon noted completion of the reaction, the mixture was cooled to room temperature and the solvent was evaporated under reduced pressure. The crude compound(s) was subjected to flash column chromatography (EtOAc/hexanes) to yield pure compounds **7a**–**k**.

**General procedure for 8a–j:** Into a clear solution of 2-(Boc-amino)benzaldehyde **1** (1 mmol) in methanol (5 mL) was added amine **2** (1 mmol) and stirred for 5 minutes at room temperature. Then, 3-chloropropanoic acid (**3**, 1 mmol) and methyl isocyanide (**4**, 1 mmol) were added to the reaction pot. The reaction was allowed to stir until no noticeable amounts of starting material were visible using TLC. Upon completion of the reaction, methanol was evaporated under reduced pressure and the crude Ugi products, without any purification, was dissolved in a CH_2_Cl_2_ and trifluoroacetic acid mixture (1:1, 2 mL). The reaction was then allowed to stir at room temperature for 5 h and monitored by TLC. Upon completion of the reaction, the solvent was evaporated under reduced pressure and the crude compound, without any purification, was dissolved in acetonitrile (2 mL) and K_2_CO_3_ (2 mmol) was added. The reaction was then allowed to stir under refluxing conditions for 1 h and reaction completion was monitored using TLC. The reaction mixture was then allowed to cool to room temperature and the solvent evaporated under reduced pressure. The crude products were subjected to flash column chromatography (EtOAc/hexanes) to yield pure compounds **8a**–**j**.

## Supporting Information

File 1 ^1^H and ^13^C NMR spectroscopic data for compounds **7a**–**k** and **8a**–**j**, and X-ray crystal structure details for **7a**.
