# Supplementary material for: Methyl isocyanide as a convertible functional group for the synthesis of spirocyclic oxindole γ-lactams via post-Ugi-4CR/transamidation/cyclization in a one-pot, three-step sequence
Source: Beilstein J Org Chem. 2018 Apr 18;14:875–83. doi: 10.3762/bjoc.14.74 (PMC5942382; doi:10.3762/bjoc.14.74)
Supplement: File 1 — 1H and 13C NMR spectroscopic data for compounds 7a–k and 8a–j, and X-ray crystal structure details for 7a. [file Beilstein_J_Org_Chem-14-875-s001.pdf]

Supporting Information

for

Methyl isocyanide as a convertible functional group for the synthesis of spirocyclic oxindole γ–lactams via post Ugi-4CR/transamidation/cyclization in a one-pot, three-step sequence

Amarendar Reddy Maddirala[1] and Peter R. Andreana[1]*

Address: [1]Department of Chemistry and Biochemistry *and* School of Green Chemistry and Engineering, University of Toledo, 2801 W. Bancroft Street, Toledo, OH 43606

Email: Peter R. Andreana - peter.andreana@utoledo.edu

*Corresponding author

^{1}H and ^{13}C NMR spectroscopic data for compounds 7a–k and 8a–j, and X-ray crystal structure details for 7a

Table of Content

Experimental data for compounds **7a**–**7k** and **8a**–**8j**	S2
^{1}H and ^{13}C NMR spectral data for compounds **7a**–**7k**	S10
^{1}H and ^{13}C NMR spectral data for compounds **8a**–**8j**	S32
X-ray crystallographic analysis for compound **7a**	S52

Experimental data

3'-Methyl-1'-phenylspiro[indoline-3,2'-pyrrole]-2,5'(1'*H*)-dione (7a): The compound was obtained as an orange solid; m.p.: 178-180 °C; yield: (107 mg, 82%); ^{1}H NMR (600 MHz, DMSO-d_6): δ11.10 (s, 1H), 7.31-7.28 (m, 1H), 7.24 (t, *J* = 8.1 Hz, 2H), 7.15 (d, *J* = 7.4 Hz, 1H), 7.12-7.08 (m, 3H), 7.02 (t, *J* = 7.5 Hz, 1H), 6.94 (d, *J*= 7.8 Hz, 1H), 6.31 (q, *J* = 1.4 Hz, 1H), 1.64 (d, *J*= 1.3 Hz, 3H) ppm; ^{13}C NMR (150 MHz, DMSO-d_6): δ172.78, 170.91, 156.93, 142.19, 136.78, 130.60, 128.92, 125.96, 124.30, 124.19, 123.94, 123.33, 122.99, 110.94, 77.06, 11.91 ppm; HRMS (ESI-Q-TOF): $[M+H]^+$ calculated for $C_{18}H_{15}N_2O_2$: 291.1134; found: 291.1138.

3'-Methyl-1'-(4-nitrophenyl)spiro[indoline-3,2'-pyrrole]-2,5'(1'*H*)-dione (7b): The compound was obtained as a yellow solid; m.p.: 140-142 °C; yield: (111 mg, 74%); ^{1}H NMR (600 MHz, DMSO-d_6): δ1.42 (s, 1H), 8.17 (t, *J* = 3.2 Hz, 1H), 8.15 (d, *J* = 2.2 Hz, 1H), 7.43 (t, *J* = 3.2 Hz, 1H), 7.42 (t, *J* = 2.2 Hz, 1H), 7.39-7.36 (m, 1H), 7.13 (d, *J*= 7.4 Hz, 1H), 7.08 (d, *J* = 7.8 Hz, 1H), 7.04-7.01 (m, 1H), 6.41 (q, *J* = 1.6 Hz, 1H), 1.66 (d, *J*= 1.5 Hz, 3H) ppm; ^{13}C NMR (150 MHz, DMSO-d_6): δ172.08, 171.09, 158.53, 143.17, 142.85, 141.87, 131.05, 124.89, 123.95, 123.67, 123.39, 122.93, 120.24, 111.51, 76.50, 11.71 ppm; HRMS (ESI-Q-TOF): $[M+H]^+$calculated for $C_{18}H_{14}N_3O_4$: 336.0984; found: 336.0996.

1'-(4-Methoxyphenyl)-3'-phenylspiro[indoline-3,2'-pyrrole]-2,5'(1'*H*)-dione (7c): The compound was obtained as an off-white solid; m.p.: 146-148 °C; yield: (130 mg, 75%); ^{1}H NMR (600 MHz, DMSO-d_6): δ10.86 (s, 1H), 7.37-7.31 (m, 3H), 7.27-7.26 (m, 2H), 7.06 (s, 1H), 6.95 (d, *J* = 2.5 Hz, 1H), 6.91-6.89 (m, 2H), 6.84-6.82 (m, 3H), 6.78 (d, *J* = 8.5 Hz, 1H), 3.68 (s, 3H), 3.65 (s, 3H) ppm; ^{13}C NMR (150 MHz, DMSO-d_6): δ173.02, 170.32, 158.62, 130.74, 130.27, 129.08, 129.02, 128.00, 126.51, 126.46, 123.98, 116.08, 114.17, 111.64, 111.23, 76.07, 55.61,

55.26 ppm; HRMS (ESI-Q-TOF): $[M+H]^+$calculated for $C_{25}H_{21}N_2O_4$: 413.1501; found: 413.1518.

1'-(4-Bromophenyl)-3'-propylspiro[indoline-3,2'-pyrrole]-2,5'(1'*H*)-dione (7d): The compound was obtained as a light-yellow solid; m.p.: 226-228 °C; yield: (133 mg, 78%); ^{1}H NMR (600 MHz, DMSO-d_6): δ11.15 (s, 1H), 7.47-7.44 (m, 2H), 7.33-7.30 (m, 1H), 7.15 (d, J = 7.3 Hz, 1H), 7.04-7.00 (m, 3H), 6.96 (d, J = 7.8 Hz, 1H), 6.33 (t, J = 1.6 Hz, 1H), 1.93-1.87 (m, 1H), 1.74-1.68 (m, 1H), 1.46-1.37 (m, 2H), 0.77 (t, J= 7.4 Hz, 3H) ppm; ^{13}C NMR (150 MHz, DMSO-d_6): δ172.70, 170.90, 161.30, 142.21, 136.00, 131.92, 130.79, 125.66, 124.34, 124.07, 123.05, 122.04, 118.39, 111.06, 76.69, 27.94, 19.54, 13.42 ppm; HRMS (ESI-Q-TOF): $[M+H]^+$calculated for $C_{20}H_{18}BrN_2O_2$: 397.0552; found: 397.0544.

5-Chloro-1'-cyclopropyl-3'-methylspiro[indoline-3,2'-pyrrole]-2,5'(1'*H*)-dione (7e): The compound was obtained as an off-white solid; m.p.: 284-286 °C; yield: (86 mg, 76%); ^{1}H NMR (600 MHz, DMSO-d_6): δ11.17 (s, 1H),7.41 (dd, J_1 = 6.2 Hz,J_2 = 2.2 Hz, 1H), 7.17-7.16 (d, J = 2.1 Hz, 1H),7.01-7.00 (d, J = 8.3 Hz, 1H), 6.09 (d, J = 1.6 Hz, 1H),2.42-2.20 (m, 1H),1.56 (d, J = 1.5 Hz, 3H), 0.51-0.43 (m, 3H), 0.34-0.31 (m, 1H) ppm; ^{13}C NMR (150 MHz, DMSO-d_6): δ173.19, 172.69, 155.62, 141.44, 130.38, 127.06, 126.87, 124.07, 123.90, 112.24, 76.44, 23.13, 11.91, 3.99 ppm; HRMS (ESI-Q-TOF): $[M+H]^+$calculated for $C_{15}H_{14}ClN_2O_2$: 289.0744; found: 297.0753.

1'-Isopropyl-5,6-dimethoxy-3'-methylspiro[indoline-3,2'-pyrrole]-2,5'(1'*H*)-dione (7f): The compound was obtained as a pink solid; m.p.: 242-244 °C; yield: (67 mg, 60%); ^{1}H NMR (600 MHz, DMSO-d_6): δ10.77 (s, 1H), 6.66 (s, 1H), 6.61 (s, 1H), 6.00 (d, J = 1.6 Hz, 1H), 3.79 (s, 3H), 3.66 (s, 3H), 3.39-3.34 (m, 1H), 1.52 (d, J = 1.5 Hz, 3H), 1.10 (t, J = 6.9 Hz, 6H) ppm; ^{13}C

NMR (150 MHz, DMSO-d_6): δ173.98, 171.06, 155.49, 150.71, 144.91, 136.21, 123.95, 114.17, 108.81, 69.24, 76.33, 56.30, 55.67, 45.32, 20.41, 20.10, 11.78 ppm; HRMS (ESI-Q-TOF): $[M+H]^+$calculated for $C_{17}H_{21}N_2O_4$: 317.1501; found: 317.1499.

5-Methoxy-1'-(3-methoxybenzyl)-3'-ethylspiro[indoline-3,2'-pyrrole]-2,5'(1'*H*)-dione (7g): The compound was obtained as a yellow solid; m.p.: 70-72 °C; yield: (122 mg, 81%); ^{1}H NMR (600 MHz, DMSO-d_6): δ10.73 (s, 1H), 7.02 (t, J = 7.9 Hz, 1H), 6.79-6.78 (m, 2H), 6.97 (d, J = 7.2 Hz,1H), 6.67 (dd, J_1 = 5.9 Hz, J_2 = 2.3 Hz, 1H), 6.56 (d, J = 7.6 Hz, 1H), 6.49 (s, 1H), 6.29 (d, J = 1.6 Hz, 1H), 6.20 (t, J = 1.6 Hz, 1H), 4.35 (d, J = 15.4 Hz, 1H),3 .95 (d, J = 15.4 Hz, 1H), 3.61 (s, 1H), 3.53 (s, 1H), 1.88-1.84 (m, 1H), 1.83-1.75 (m, 1H), 0.97-0.95 (t, J= 7.4 Hz, 3H) ppm; ^{13}C NMR (150 MHz, DMSO-d_6): δ172.55, 171.65, 162.43, 158.80, 155.14, 138.50, 135.65, 128.91, 124.87, 121.28, 120.18, 115.97, 113.26, 112.70, 111.09, 110.32, 75.99, 55.28, 54.76, 43.85, 19.50, 11.01 ppm; HRMS (ESI-Q-TOF): $[M+H]^+$calculated for $C_{22}H_{23}N_2O_4$: 379.1658; found: 379.1651.

1'-Benzyl-3'-phenyl-6-(trifluoromethyl)spiro[indoline-3,2'-pyrrole]-2,5'(1'*H*)-dione (7h): The compound was obtained as an off-white solid; m.p.: 188-190 °C; yield: (114 mg, 76%); ^{1}H NMR (600 MHz, DMSO-d_6): δ11.44 (s, 1H), 7.34-7.29 (m, 3H), 7.14-7.05 (m, 6H), 6.97 (d, J = 7.8 Hz, 1H), 6.94-6.93 (m, 2H), 4.41 (d, J= 15.6 Hz, 1H), 4.04 (d, J= 15.6 Hz, 1H) ppm; ^{13}C NMR (150 MHz, DMSO-d_6): δ172.43, 170.52, 154.75, 143.33, 136.61, 132.00, 130.29, 130.25, 129.13, 127.85, 127.79, 127.14, 126.13, 126.02, 124.51, 123.99, 119.60, 107.09, 73.21, 43.50 ppm; HRMS (ESI-Q-TOF): $[M+H]^+$calculated for $C_{25}H_{18}F_3N_2O_2$: 435.1242; found: 435.1313.

1'-(4-Chlorobenzyl)-3'-ethylspiro[indoline-3,2'-pyrrole]-2,5'(1'*H*)-dione (7i): The compound was obtained as an off-white solid; m.p.: 149-151 °C; yield: (124 mg, 78%); ^{1}H NMR (600

MHz, DMSO-d_6): δ10.91 (s, 1H), 7.28-7.25 (m, 1H), 7.18-7.16 (m, 2H), 6.97-6.95 (m, 1H), 6.89-6.86 (m, 2H), 6.77 (d, *J* = 7.3 Hz, 1H), 6.22 (t, *J* = 1.8 Hz, 1H), 4.16 (q, *J* = 15.6 Hz, 2H), 1.88-1.81 (m, 1H), 1.74-1.67 (m, 1H), 0.95 (t, *J*= 7.3 Hz, 3H) ppm; ^{13}C NMR (150 MHz, DMSO-d_6): δ172.57, 171.69, 162.61, 142.64, 135.86, 131.58, 130.42, 129.76, 127.80, 124.54, 123.57, 122.41, 121.28, 110.60, 75.40, 43.08, 40.04, 19.52, 11.02 ppm; HRMS (ESI-Q-TOF): $[M+H]^+$calculated for $C_{20}H_{18}ClN_2O_2$: 353.1057; found: 353.1049.

3-Ethyl-1-isopropylspiro[pyrrole-2,7'-[1,3]dioxolo[4,5-*f*]indole]-5,6'(1*H*,5'*H*)-dione (7j): The compound was obtained as a light-yellow solid; m.p.: 241-243 °C; yield: (75 mg, 63%); ^{1}H NMR (600 MHz, DMSO-d_6): δ10.81 (s, 1H), 6.69 (s, 1H), 6.62 (s, 1H), 6.02 (s, 1H), 6.00 (s, 1H), 5.99 (t, *J* = 2.3 Hz, 1H), 3.34-3.29 (m, 2H), 1.82-1.75 (m, 1H), 1.71-1.64 (m, 1H), 1.12-1.09 (m, 6H), 0.94 (t, *J* = 7.4 Hz, 3H) ppm; ^{13}C NMR (150 MHz, DMSO-d_6): δ173.95, 170.99, 161.21, 148.63, 143.05, 137.01, 122.26, 115.69, 105.37, 101.30, 94.05, 75.95, 45.27, 40.04, 20.32, 20.15, 19.11, 10.96 ppm; HRMS (ESI-Q-TOF): $[M+H]^+$calculated for $C_{17}H_{19}N_2O_4$: 315.1345; found: 315.1348.

1'-(2-Bromobenzyl)-3'-phenylspiro[indoline-3,2'-pyrrole]-2,5'(1'*H*)-dione (7k): The compound was obtained as a white solid; m.p.: 167-169 °C; yield: (165 mg, 82%); ^{1}H NMR (600 MHz, DMSO-d_6): δ11.11 (s, 1H), 7.38 (t, *J* = 7.9 Hz, 1H), 7.33-7.27 (m, 3H), 7.25-7.19 (m, 5H), 7.09-7.06 (m, 2H), 6.92(dd, J_1 = 7.7 Hz, J_2 = 7.4 Hz, 2H), 6.38 (t, *J* = 7.6 Hz, 1H), 4.36 (d, *J* = 16.2 Hz, 1H), 4.25 (d, *J*= 16.2 Hz, 1H) ppm; ^{13}C NMR (150 MHz, DMSO-d_6): δ172.54, 170.84, 155.81, 142.70, 135.10, 132.02, 130.85, 130.58, 130.17, 129.83, 129.13, 127.42, 126.28, 124.86, 124.03, 123.35, 122.75, 122.21, 111.08, 73.85, 43.28 ppm; HRMS (ESI-Q-TOF): $[M+Na]^+$calculated for $C_{24}H_{17}BrN_2NaO_2$: 467.0371; found: 467.0363.

5-Chloro-1'-phenylspiro[indoline-3,2'-pyrrolidine]-2,5'-dione (8a): The compound was obtained as an off-white solid; m.p.: 268-270 °C; yield: (98 mg, 80%); ^{1}H NMR (600 MHz, DMSO-d_6): δ10.77 (s, 1H), 7.63 (d, *J* = 2.2 Hz, 1H), 7.28-7.23 (m, 3H), 7.18-7.15 (m, 1H), 6.97-6.95 (m, 2H), 6.79 (d, *J* = 8.3 Hz, 1H), 2.79-2.69 (m, 2H), 2.46-2.42 (m, 1H), 2.37-2.32 (m, 1H) ppm; ^{13}C NMR (150 MHz, DMSO-d_6): δ177.52, 174.84, 140.32, 136.62, 130.81, 129.69, 128.93, 127.06, 126.48, 126.17, 124.85, 111.68, 69.91, 30.65, 29.29 ppm; HRMS (ESI-Q-TOF): $[M+H]^+$calculated for $C_{17}H_{14}ClN_2O_2$: 313.0744; found: 313.0747.

1'-Cyclopentylspiro[indoline-3,2'-pyrrolidine]-2,5'-dione (8b): The compound was obtained as a white solid; m.p.: 250-252 °C; yield: (99 mg, 81%); ^{1}H NMR (600 MHz, DMSO-d_6): δ10.62 (s, 1H), 7.36 (d, *J* = 7.0 Hz, 1H), 7.30-7.27 (m, 1H), 7.05 (t, *J* = 7.3 Hz, 1H), 6.88 (t, *J* = 7.4 Hz, 1H), 3.14-3.11 (m, 1H), 2.47-2.20 (m, 4H), 1.75-174 (m, 2H), 1.51-1.25 (m, 6H) ppm; ^{13}C NMR (150 MHz, DMSO-d_6): δ178.47, 174.89, 141.54, 129.77, 129.74, 124.16, 122.43, 110.29, 69.15, 54.92, 30.58, 29.64, 28.44, 23.70, 23.59 ppm; HRMS (ESI-Q-TOF): $[M+H]^+$calculated for $C_{16}H_{19}N_2O_2$: 271.1447; found: 271.1437.

1'-(Cyclohexylmethyl)spiro[indoline-3,2'-pyrrolidine]-2,5'-dione (8c): The compound was obtained as a white solid; m.p.: 100-102 °C; yield: (106 mg, 79%); ^{1}H NMR (600 MHz, DMSO-d_6): δ10.65 (s, 1H), 7.34 (d, *J* = 7.4 Hz, 1H), 7.29 (t, *J* = 7.6 Hz, 1H), 7.04(t, *J* = 7.4 Hz, 1H), 6.89 (d, *J* = 7.7 Hz, 1H), 2.71 (d, *J* = 7.6 Hz, 2H), 2.60-2.51 (m, 2H), 2.28-2.27 (m, 1H), 2.13-2.09 (m, 1H), 1.55-1.39 (m, 5H), 1.02-0.89 (m, 2H), 0.88-0.85 (m, 1H), 0.71-0.61 (m, 2H) ppm; ^{13}C NMR (150 MHz, DMSO-d_6): δ178.00, 175.59, 141.70, 129.90, 128.59, 124.49, 122.28, 110.23, 68.24, 46.80, 40.04, 35.79, 30.29, 30.26, 30.15, 28.98, 25.82, 25.24 ppm; HRMS (ESI-Q-TOF): $[M+H]^+$calculated for $C_{18}H_{23}N_2O_2$: 299.1760; found: 299.1767.

1'-Benzyl-5-methoxyspiro[indoline-3,2'-pyrrolidine]-2,5'-dione (8d): The compound was obtained as an off-white solid; m.p.: 128-130 °C; yield: (101 mg, 79%); ^{1}H NMR (600 MHz, $CDCl_3$): δ7.55 (s, 1H), 7.14-7.09 (m, 3H), 6.93(d, J = 6.7 Hz, 2H), 6.79 (d, J = 8.3 Hz, 1H), 6.73 (d, J = 8.3 Hz, 1H), 6.50 (s, 1H), 4.36 (d, J = 14.8 Hz, 1H), 4.20 (d, J = 14.7 Hz, 1H), 3.66 (s, 3H), 2.99-2.93 (m, 1H), 2.67-2.62 (m, 1H), 2.42-2.38 (m, 1H), 2.18-2.13 (m, 1H) ppm; ^{13}C NMR (150 MHz, DMSO-d_6): δ178.17, 176.16, 156.41, 136.22, 133.82, 129.36, 129.06, 128.15, 127.75, 115.60, 111.11, 110.98, 68.96, 55.90, 45.22, 31.37, 29.64 ppm; HRMS (ESI-Q-TOF): $[M+H]^+$calculated for $C_{19}H_{19}N_2O_3$: 323.1396; found: 323.1398.

5-Chloro-1'-(3,4-dimethoxybenzyl)spiro[indoline-3,2'-pyrrolidine]-2,5'-dione (8e): The compound was obtained as a white solid; m.p.: 157-159 °C; yield: (121 mg, 80%); ^{1}H NMR (600 MHz, DMSO-d_6): δ10.58 (s, 1H), 7.23-7.21 (dd, J_1 = 6.2 Hz, J_2 = 2.1 Hz, 1H), 7.13 (d, J = 2.0 Hz, 1H), 6.76 (d, J = 8.2 Hz, 1H), 6.67 (d, J = 8.2 Hz, 1H), 6.40-6.37 (m, 2H), 4.10 (d, J = 14.6 Hz, 1H), 3.97 (d, J= 14.8 Hz, 1H), 3.67 (s, 3H), 3.59 (s, 3H), 2.64-2.53 (m, 2H), 2.25-2.20 (m, 2H) ppm; ^{13}C NMR (150 MHz, DMSO-d_6): δ177.30, 175.08, 147.99, 140.59, 130.01, 129.39, 128.43, 126.11, 125.12, 120.79, 112.12, 111.24, 111.23, 67.92, 55.41, 55.07, 43.96, 40.04, 30.05, 28.84 ppm; HRMS (ESI-Q-TOF): $[M+H]^+$calculated for $C_{20}H_{20}ClN_2O_4$: 387.1112; found: 387.1103.

1'-(Benzo[*d*][1,3]dioxol-5-ylmethyl)spiro[indoline-3,2'-pyrrolidine]-2,5'-dione (8f): The compound was obtained as an off-white solid; m.p.: 138-140 °C; yield: (127 mg, 77%); ^{1}H NMR (600 MHz, DMSO-d_6): δ10.45 (s, 1H), 7.24-7.21 (m, 1H), 7.13 (d, J = 7.4 Hz, 1H), 6.93-6.90 (m, 1H), 6.80 (d, J = 7.7 Hz, 1H), 6.62 (d, J = 7.9 Hz, 1H), 6.43 (d, J = 1.6 Hz, 1H), 6.26-6.24 (m, 1H), 5.92-5.91 (m, 2H), 4.05 (d, J= 14.9 Hz, 1H), 3.88 (d, J = 14.9 Hz, 1H), 2.67-2.58 (m,

1H), 2.56-2.52 (m, 1H), 2.26-2.22 (m, 1H), 2.17-2.12 (m, 1H) ppm; ^{13}C NMR (150 MHz, DMSO-d_6): δ177.55, 175.27, 146.74, 146.22, 141.91, 130.12, 129.76, 127.70, 124.72, 121.99, 121.48, 109.99, 108.63, 107.52, 100.75, 67.81, 43.83, 30.28, 28.96 ppm; HRMS (ESI-Q-TOF): $[M+H]^+$calculated for $C_{19}H_{17}N_2O_4$: 337.1188; found: 337.1183.

1'-Phenethylspiro[indoline-3,2'-pyrrolidine]-2,5'-dione (8g): The compound was obtained as an off-white solid; m.p.: 84-86 °C; yield: (111 mg, 80%); ^{1}H NMR (600 MHz, DMSO-d_6): δ10.71 (s, 1H), 7.33-7.28 (m, 2H), 7.20 (t, J = 7.2 Hz, 2H), 7.14 (t, J = 7.3 Hz, 1H), 7.05-7.03 (m, 1H), 6.94 (d, J = 7.7 Hz, 1H), 6.89 (d, J = 7.1 Hz, 2H), 2.99-2.96 (m, 2H), 2.63-2.57 (m, 1H), 2.55-2.48 (m, 2H), 2.32-2.27 (m, 1H), 2.18-2.12 (m, 1H) ppm; ^{13}C NMR (150 MHz, DMSO-d_6): δ178.22, 175.15, 141.94, 138.48, 130.07, 128.47, 128.24, 128.18, 126.28, 124.63, 122.48, 110.31, 68.19, 42.43, 33.76, 29.94, 29.02 ppm; HRMS (ESI-Q-TOF): $[M+H]^+$calculated for $C_{19}H_{19}N_2O_2$: 307.1447; found: 307.1439.

1'-(2-Bromobenzyl)spiro[indoline-3,2'-pyrrolidine]-2,5'-dione (8h): The compound was obtained as a white solid; m.p.: 177-179 °C; yield: (137 mg, 82%); ^{1}H NMR (600 MHz, DMSO-d_6): δ10.55 (s, 1H), 7.37 (d, J = 8.0 Hz, 1H), 7.25-7.22 (m, 2H), 7.17 (t, J = 7.7 Hz, 1H), 7.12-7.02 (m, 2H), 6.82 (t, J = 7.5 Hz, 1H), 6.78 (d, J = 7.7 Hz, 1H), 4.26 (d, J = 16.0 Hz, 1H), 3.96 (d, J= 16.0 Hz, 1H), 2.72-2.59 (m, 2H), 2.34-2.30 (m, 1H), 2.25-2.20 (m, 1H) ppm; ^{13}C NMR (150 MHz, DMSO-d_6): δ177.42, 175.75, 141.96, 135.16, 131.92, 129.82, 129.77, 129.05, 127.53, 127.36, 124.55, 122.26, 122.06, 110.18, 68.26, 44.10, 30.14, 28.89 ppm; HRMS (ESI-Q-TOF): $[M+H]^+$calculated for $C_{18}H_{16}BrN_2O_2$: 370.0317; found: 370.0315.

1'-Isopropyl-5,6-dimethoxyspiro[indoline-3,2'-pyrrolidine]-2,5'-dione (8i): The compound was obtained as a white solid; m.p.: 206-208 °C; yield: (67 mg, 62%); ^{1}H NMR (600 MHz,

DMSO-d_6): δ10.39 (s, 1H), 7.09 (s, 1H), 6.51 (s, 1H), 3.76 (s, 3H), 3.70 (s, 3H), 3.13-3.06 (m, 1H), 2.48-2.38 (m, 2H), 2.17-2.07 (m, 2H), 1.10 (d, *J* = 6.9 Hz, 3H), 1.06 (d, *J* = 6.9 Hz, 3H) ppm; ^{13}C NMR (150 MHz, DMSO-d_6): δ179.14, 174.78, 150.33, 144.72, 135.30, 119.33, 109.67, 95.89, 69.10, 56.43, 55.70, 45.56, 30.40, 29.73, 19.80, 19.68 ppm; HRMS (ESI-Q-TOF): $[M+Na]^+$calculated for $C_{16}H_{20}N_2NaO_4$: 327.1321; found: 327.1323.

1'-(2,3,4,5-Tetrahydro-1*H*-benzo[*d*]azepin-7-yl)spiro[indoline-3,2'-pyrrolidine]-2,5'-dione (8j): The compound was obtained as a brown solid; m.p.: 205-207 °C; yield: (102 mg, 65%); ^{1}H NMR (600 MHz, DMSO-d_6): δ10.61 (s, 1H), 7.45 (d, *J* = 7.4 Hz, 1H), 7.19 (t, *J* = 7.6 Hz,1H), 6.98 (t, *J* = 7.5 Hz, 1H), 6.92 (d, *J* = 8.0 Hz, 1H), 6.78-6.76 (d, *J* = 7.7 Hz,1H), 6.73 (s, 1H), 6.59 (d, *J* = 7.9 Hz, 1H), 2.78-2.72 (m, 2H), 2.69-2.64 (m, 9H), 2.43-2.39 (m, 1H), 2.31-2.24 (m, 1H) ppm; ^{13}C NMR (150 MHz, DMSO-d_6): δ177.77, 174.97, 142.98, 141.40, 141.38, 134.39, 129.70, 129.38, 128.92, 127.10, 124.55, 123.36, 122.41, 110.16, 69.93, 48.19, 48.05, 40.04, 30.86, 29.37 ppm; HRMS (ESI-Q-TOF): $[M+H]^+$calculated for $C_{21}H_{22}N_3O_2$: 347.1634; found: 347.1636.

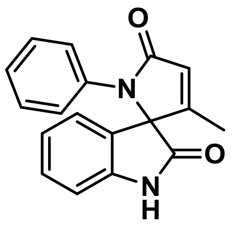

7a

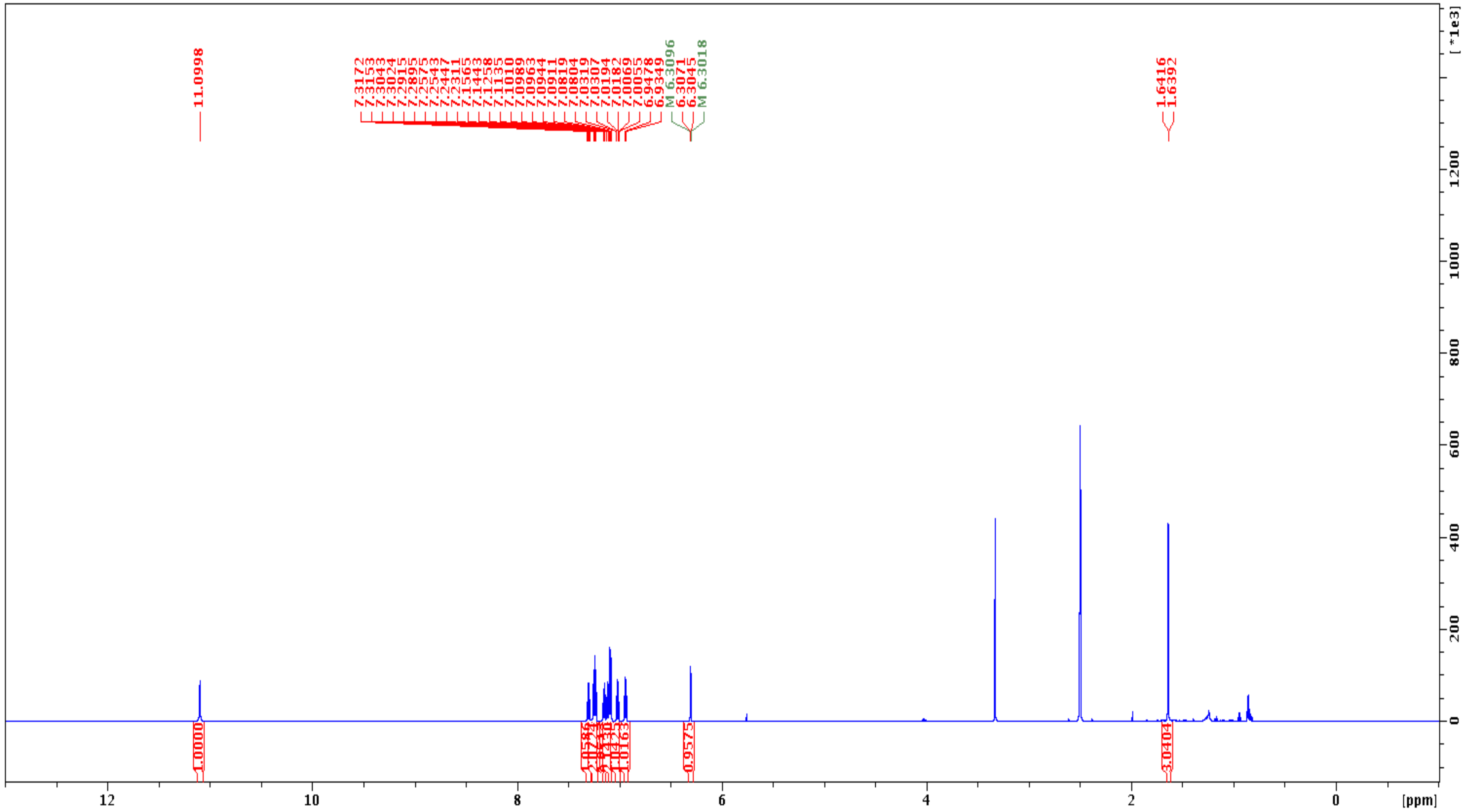

^{1}H NMR spectrum of compound **7a**

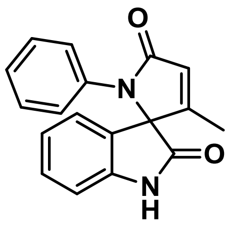

7a

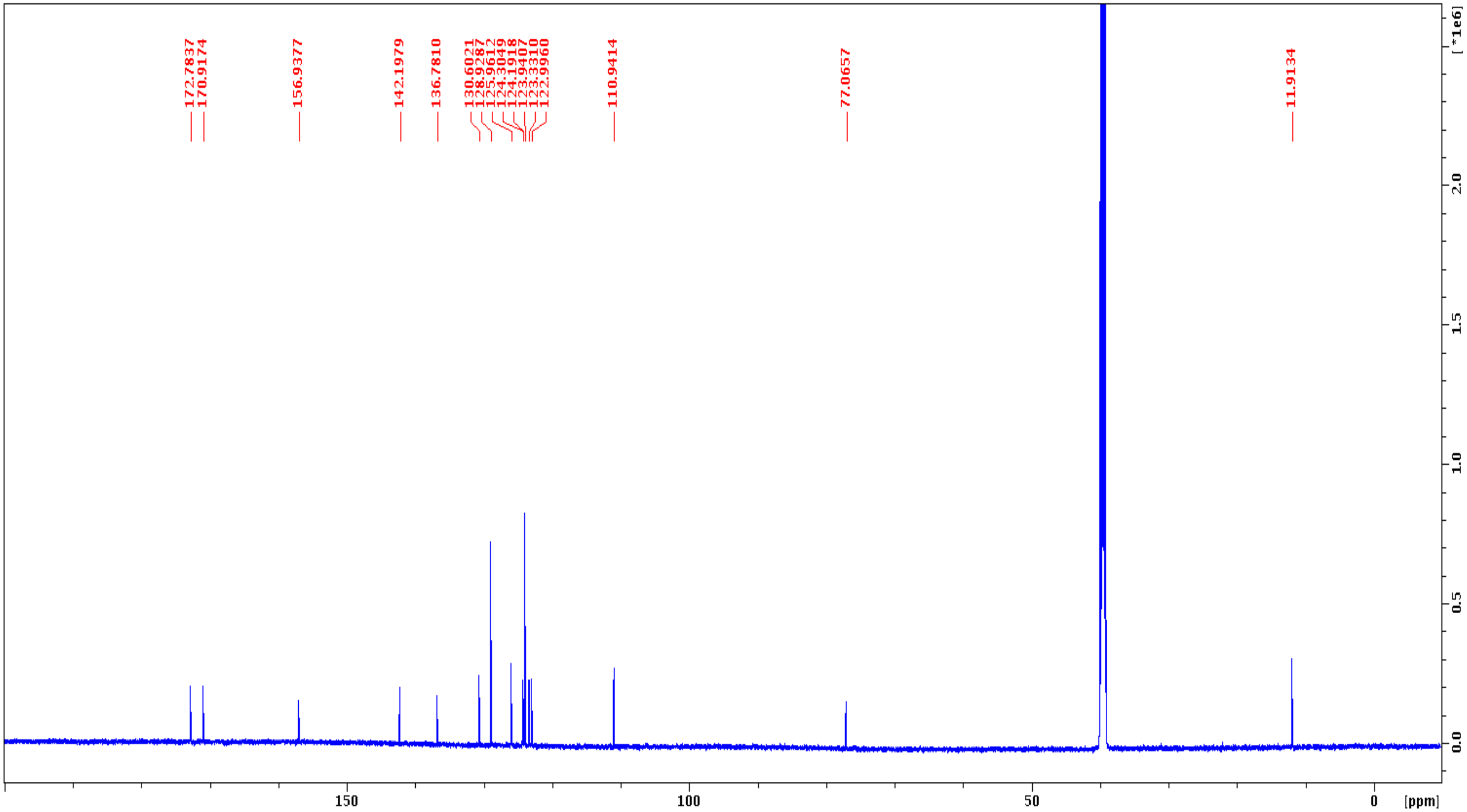

^{13}C NMR spectrum of compound **7a**

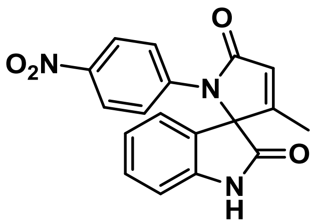

7b

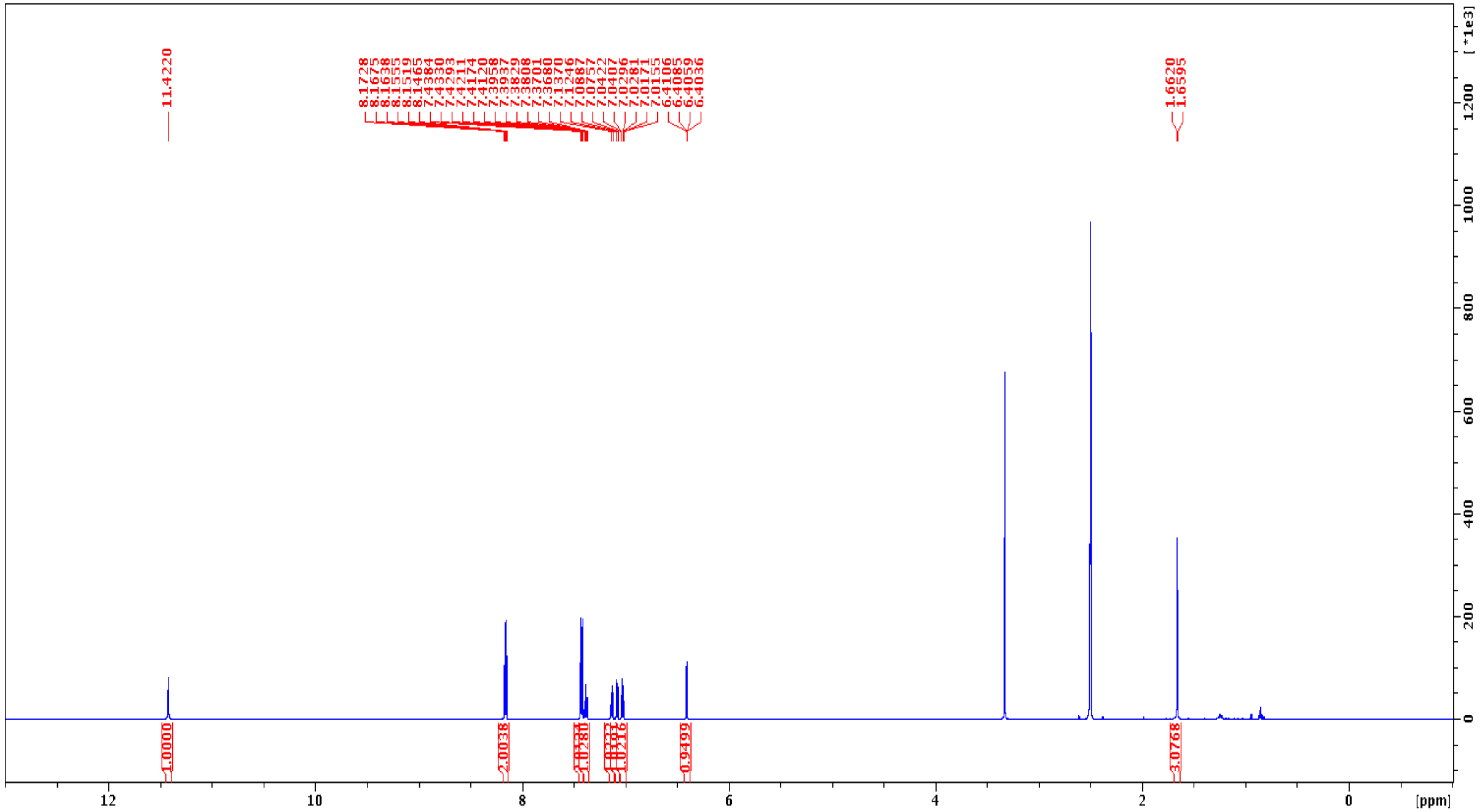

^{1}H NMR spectrum of compound **7b**

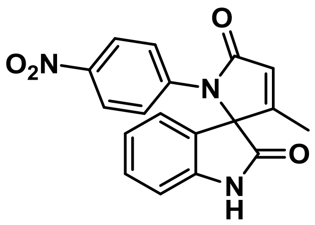

7b

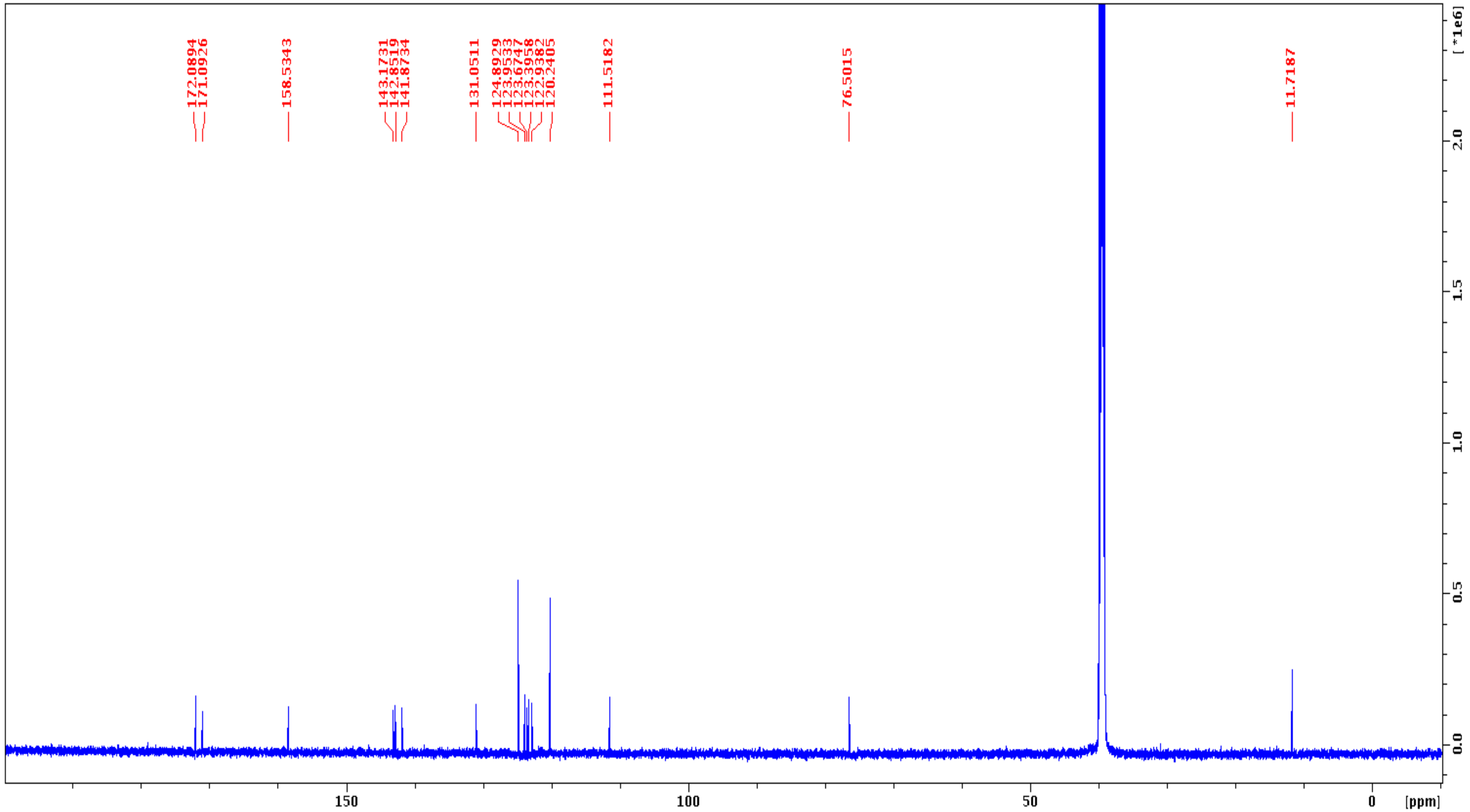

^{13}C NMR spectrum of compound **7b**

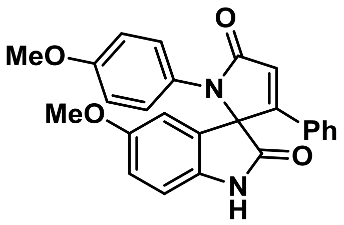

7c

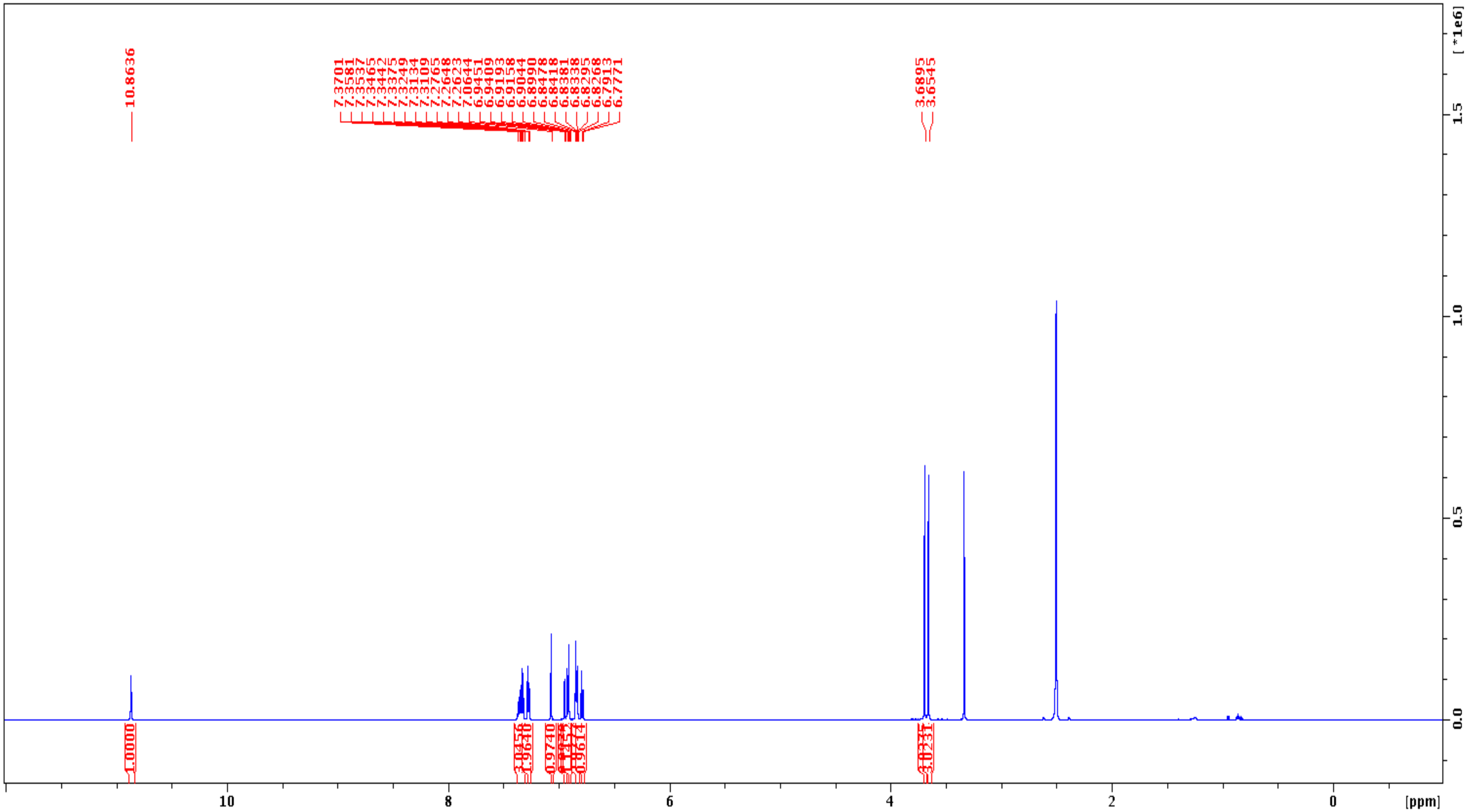

^{1}H NMR spectrum of compound **7c**

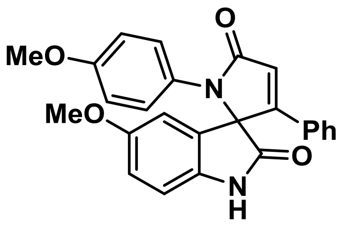

7c

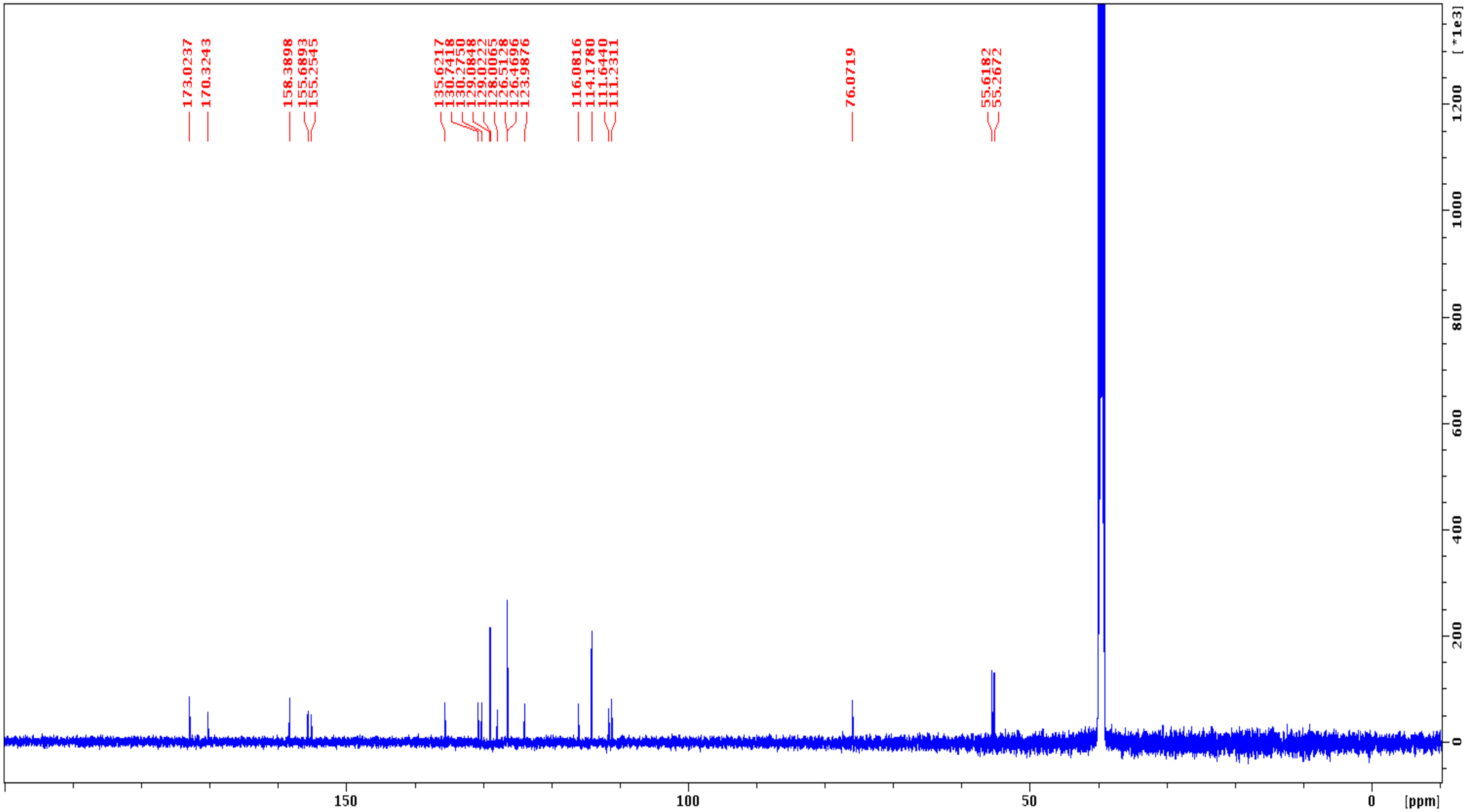

^{13}C NMR spectrum of compound **7c**

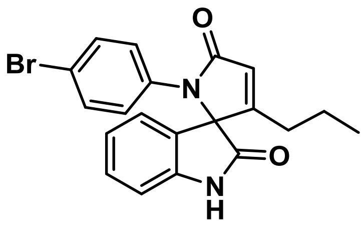

7d

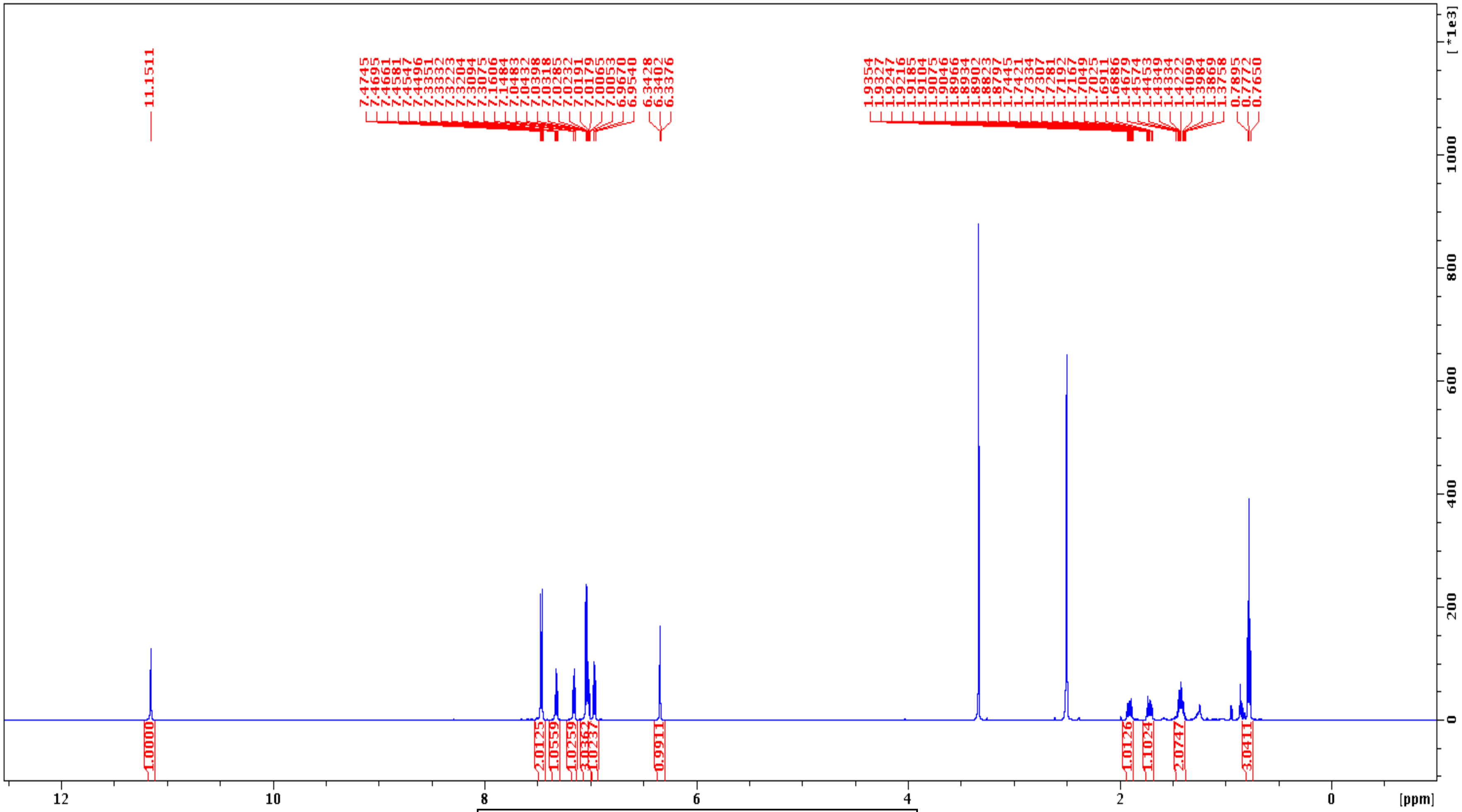

^{1}H NMR spectrum of compound **7d**

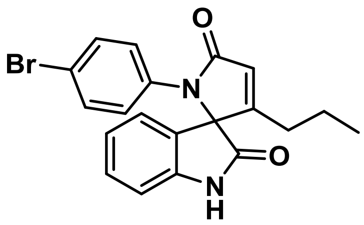

7d

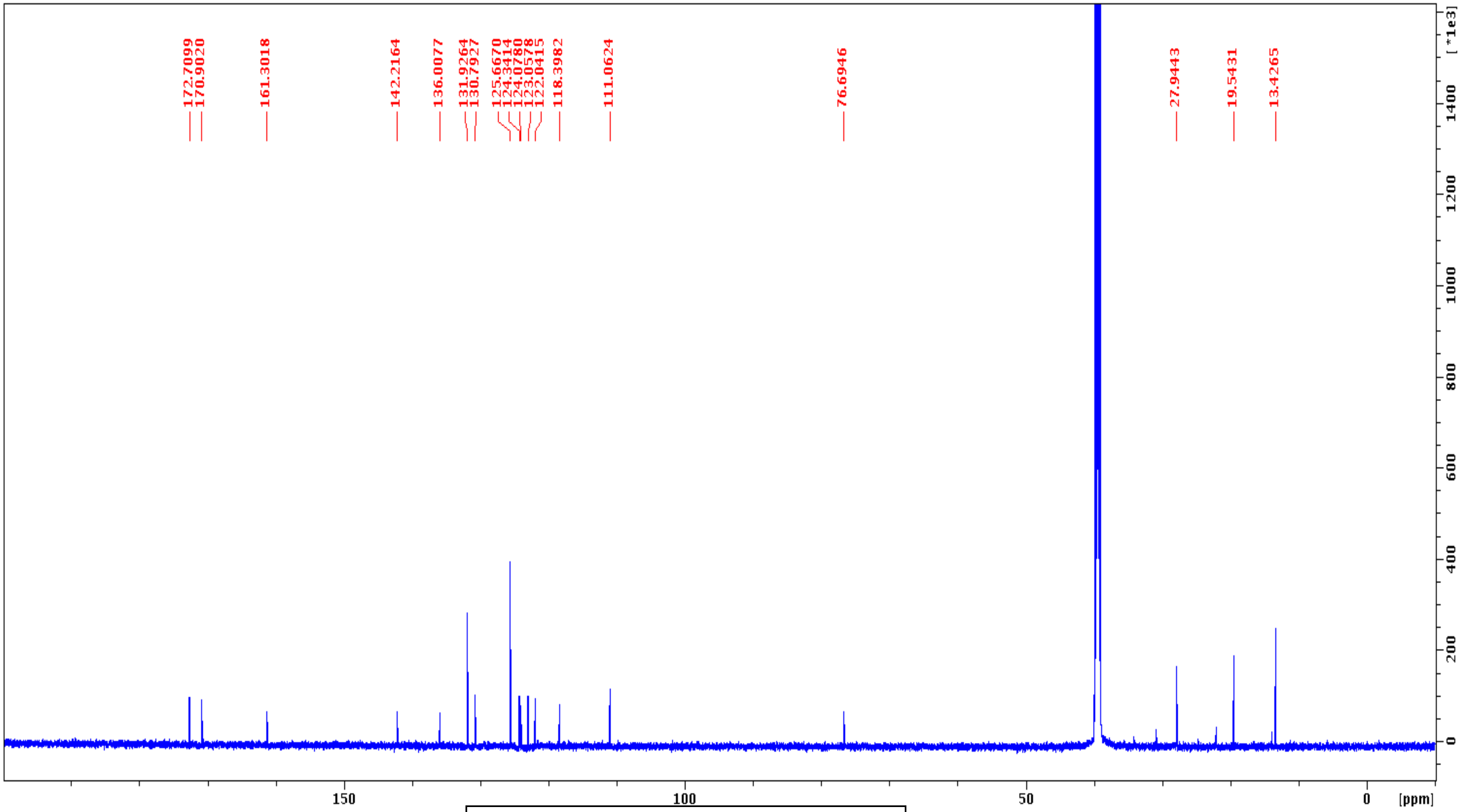

^{13}C NMR spectrum of compound **7d**

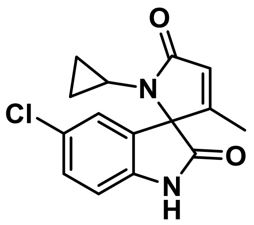

7e

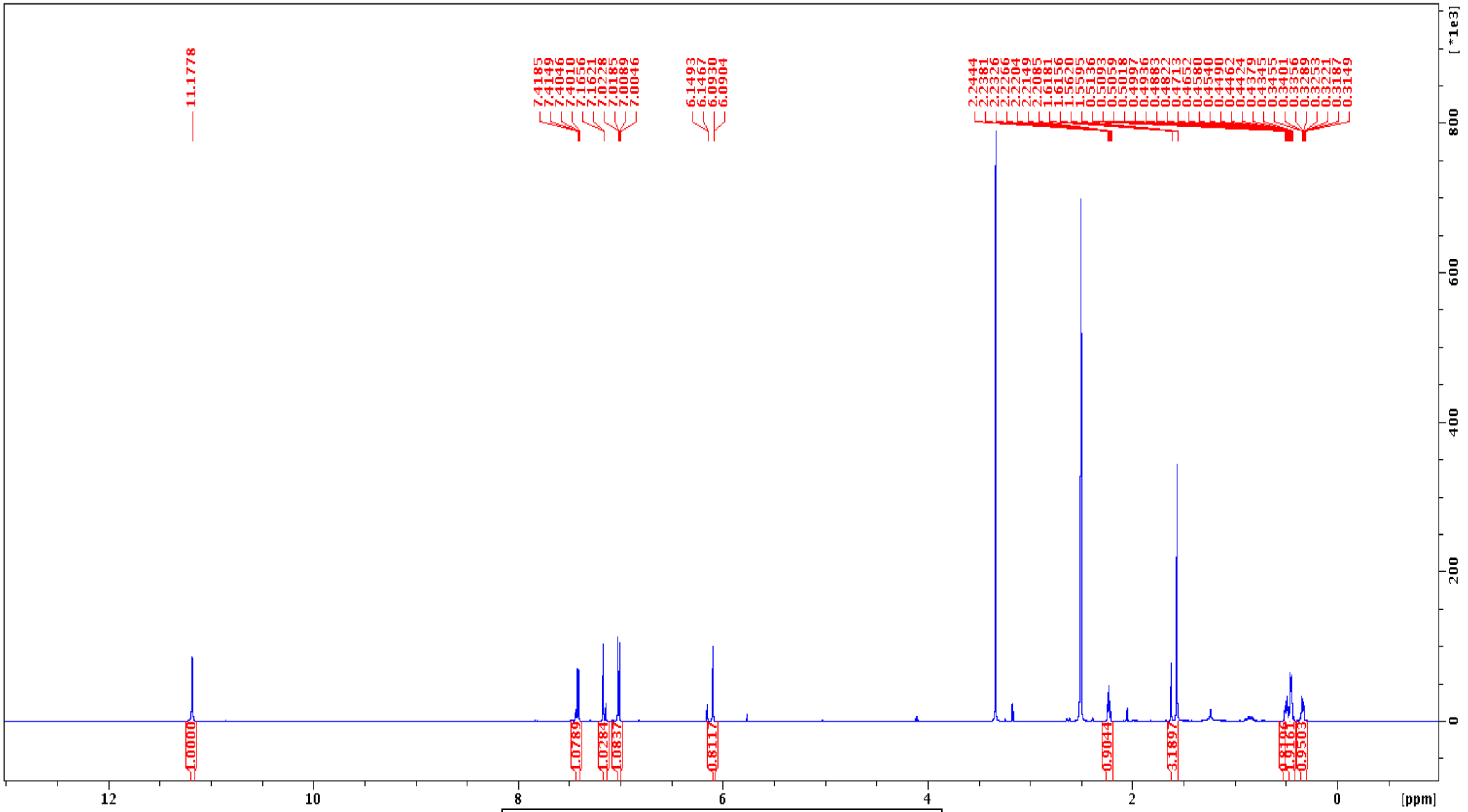

^{1}H NMR spectrum of compound **7e**

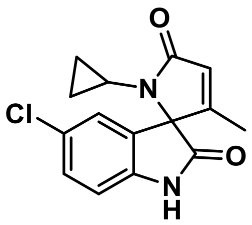

7e

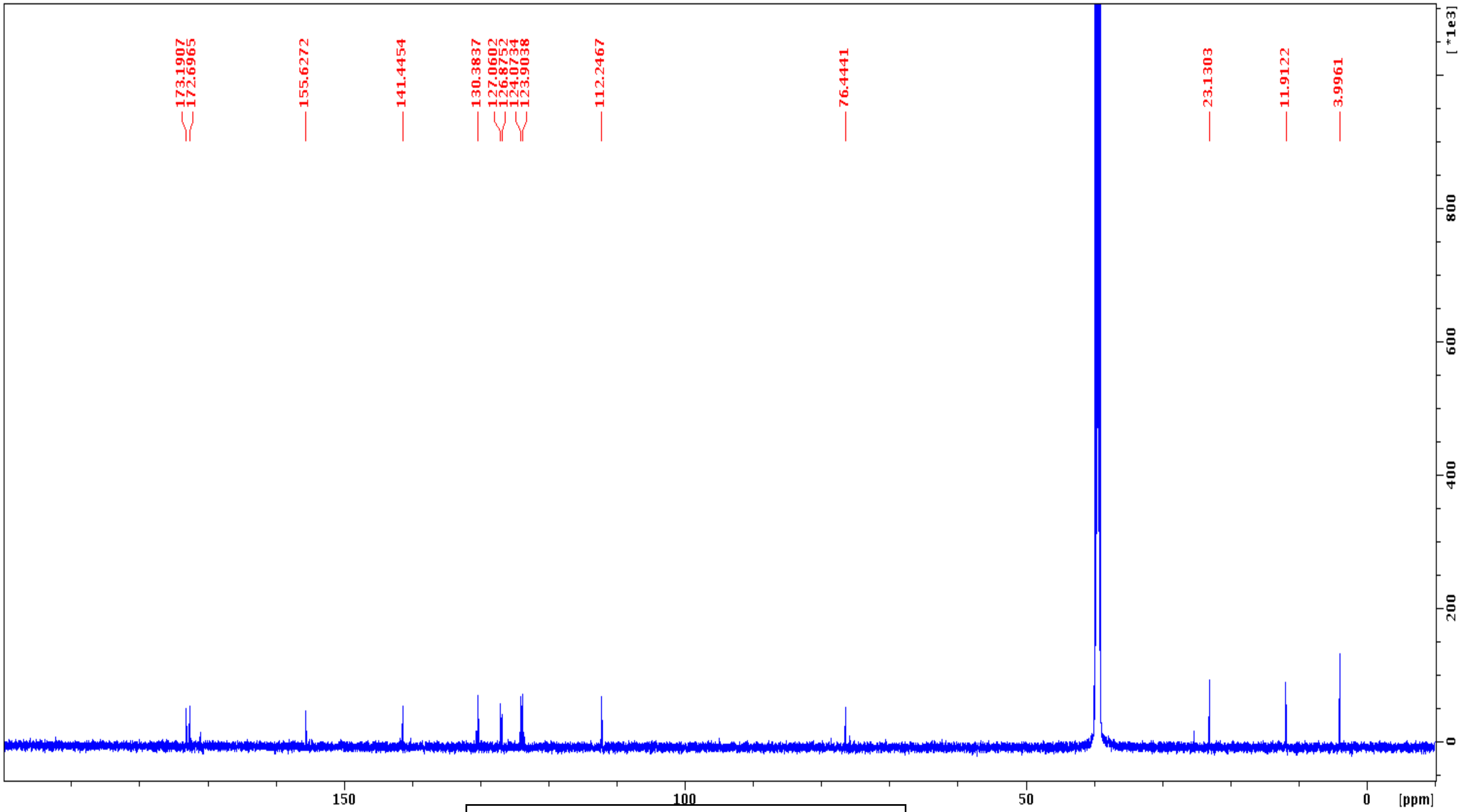

^{13}C NMR spectrum of compound **7e**

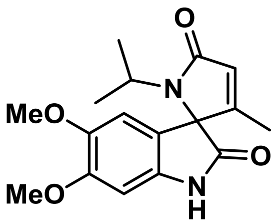

7f

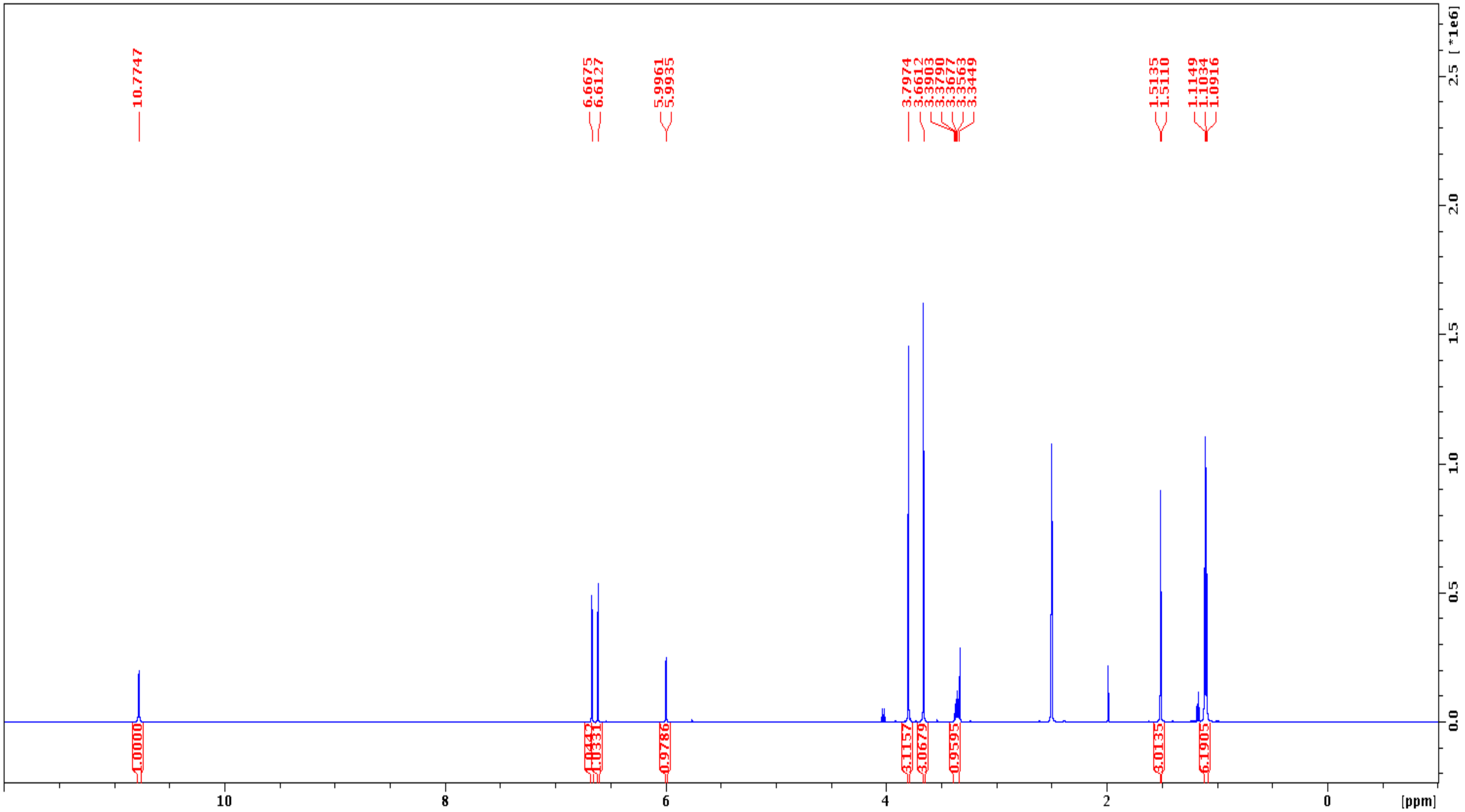

^{1}H NMR spectrum of compound **7f**

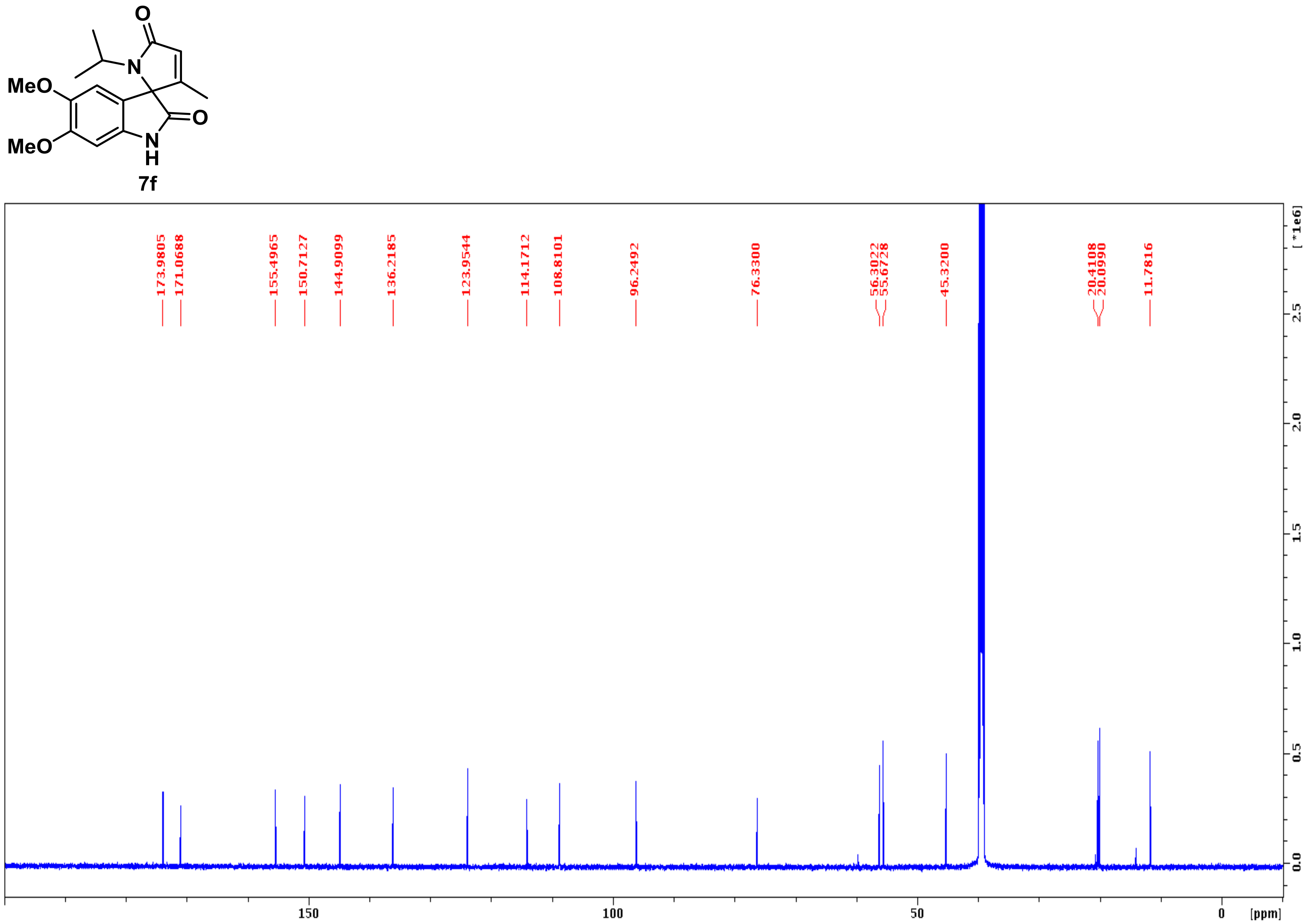

^{13}C NMR spectrum of compound **7f**

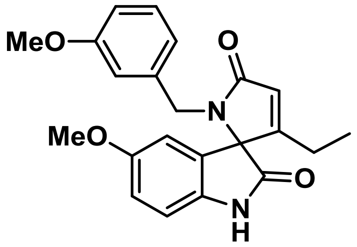

7g

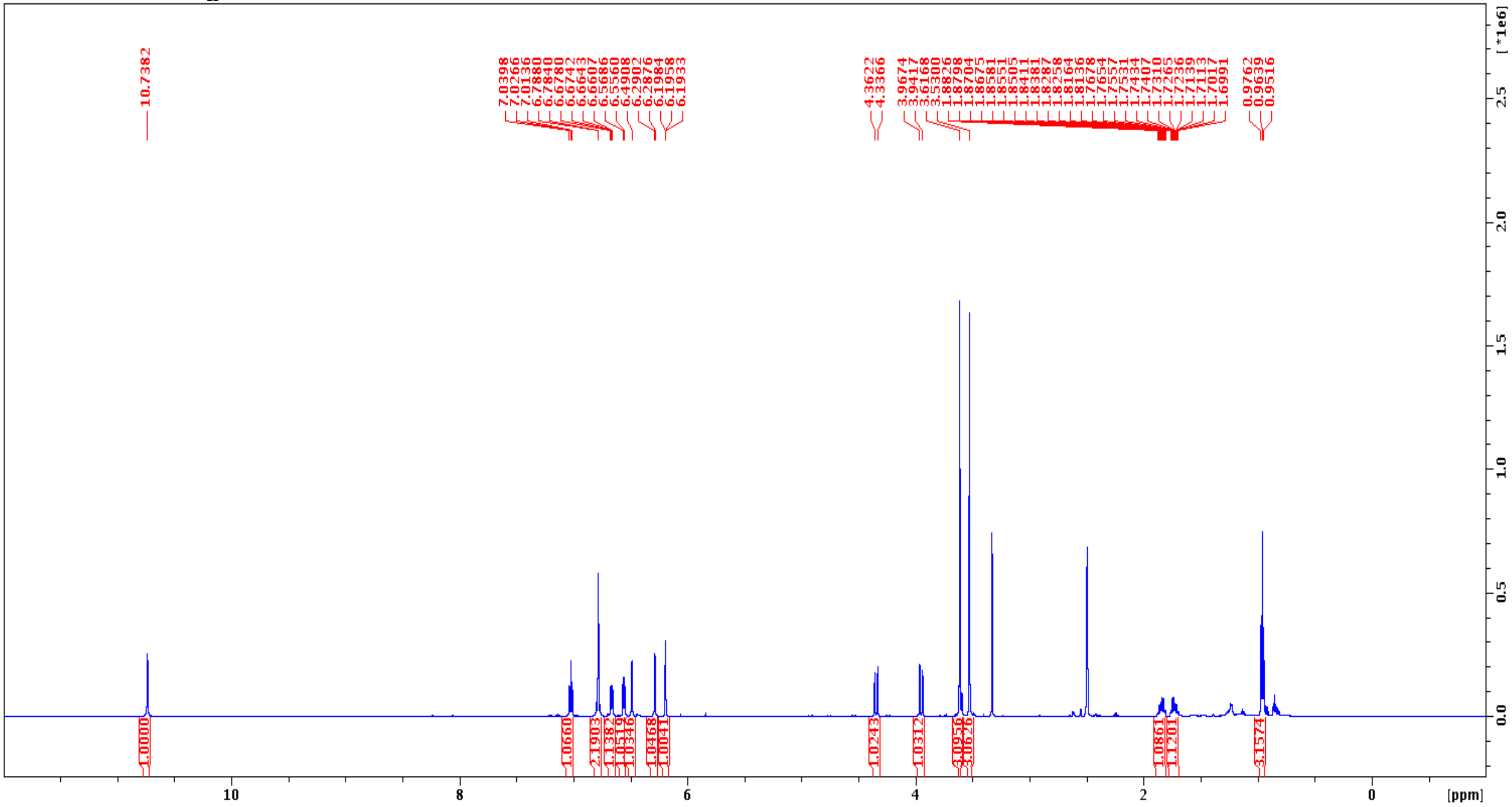

^{1}H NMR spectrum of compound **7g**

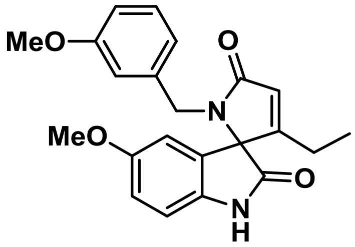

7g

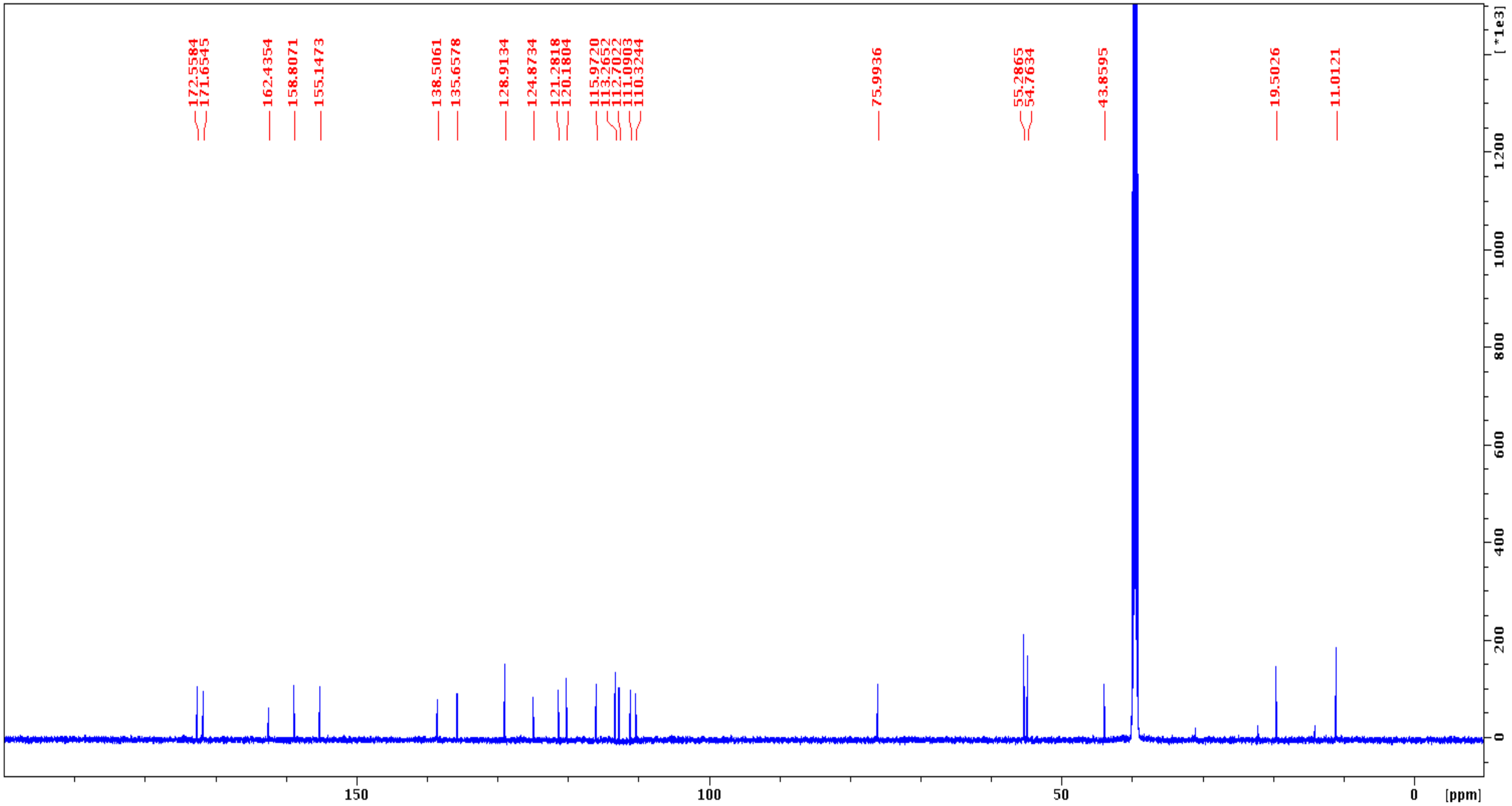

^{13}C NMR spectrum of compound **7g**

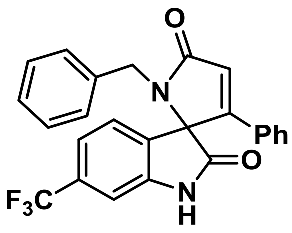

7h

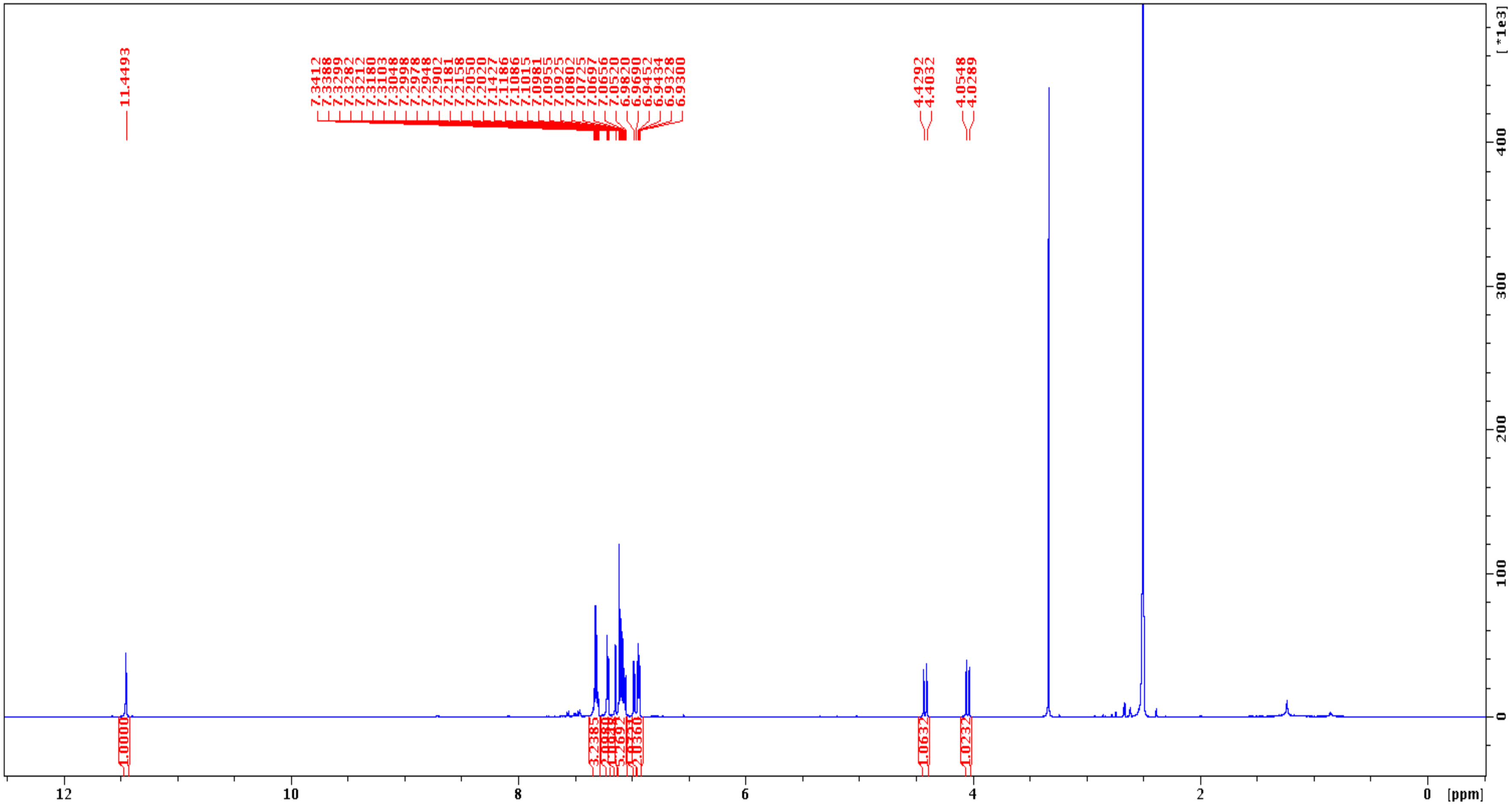

^{1}H NMR spectrum of compound **7h**

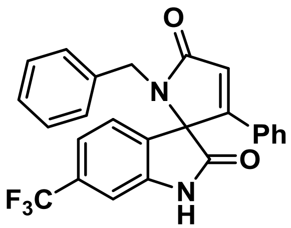

7h

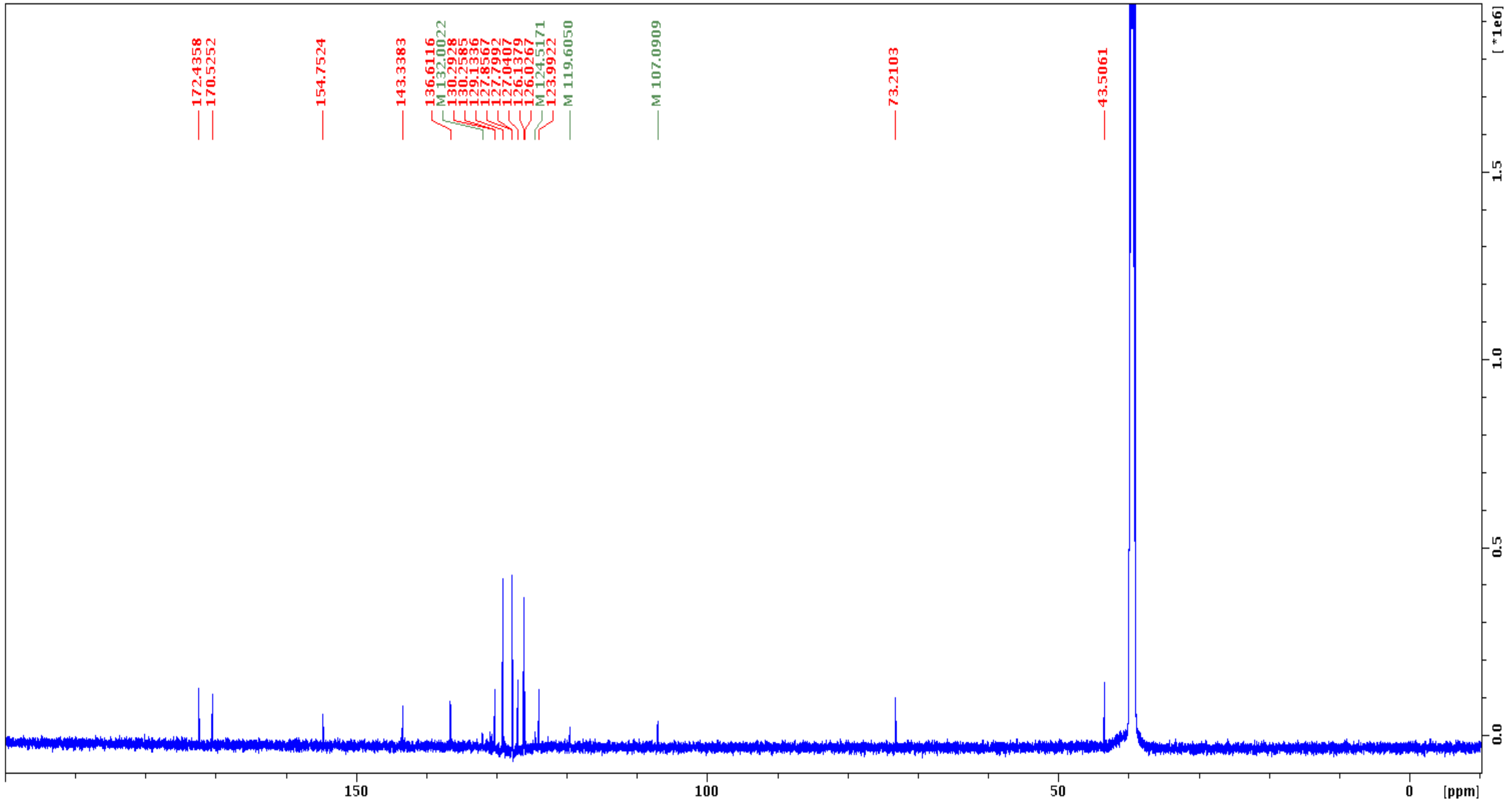

^{13}C NMR spectrum of compound **7h**

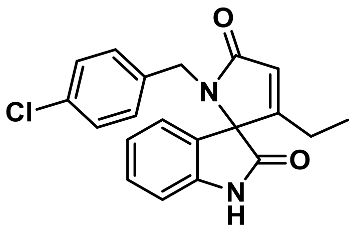

7i

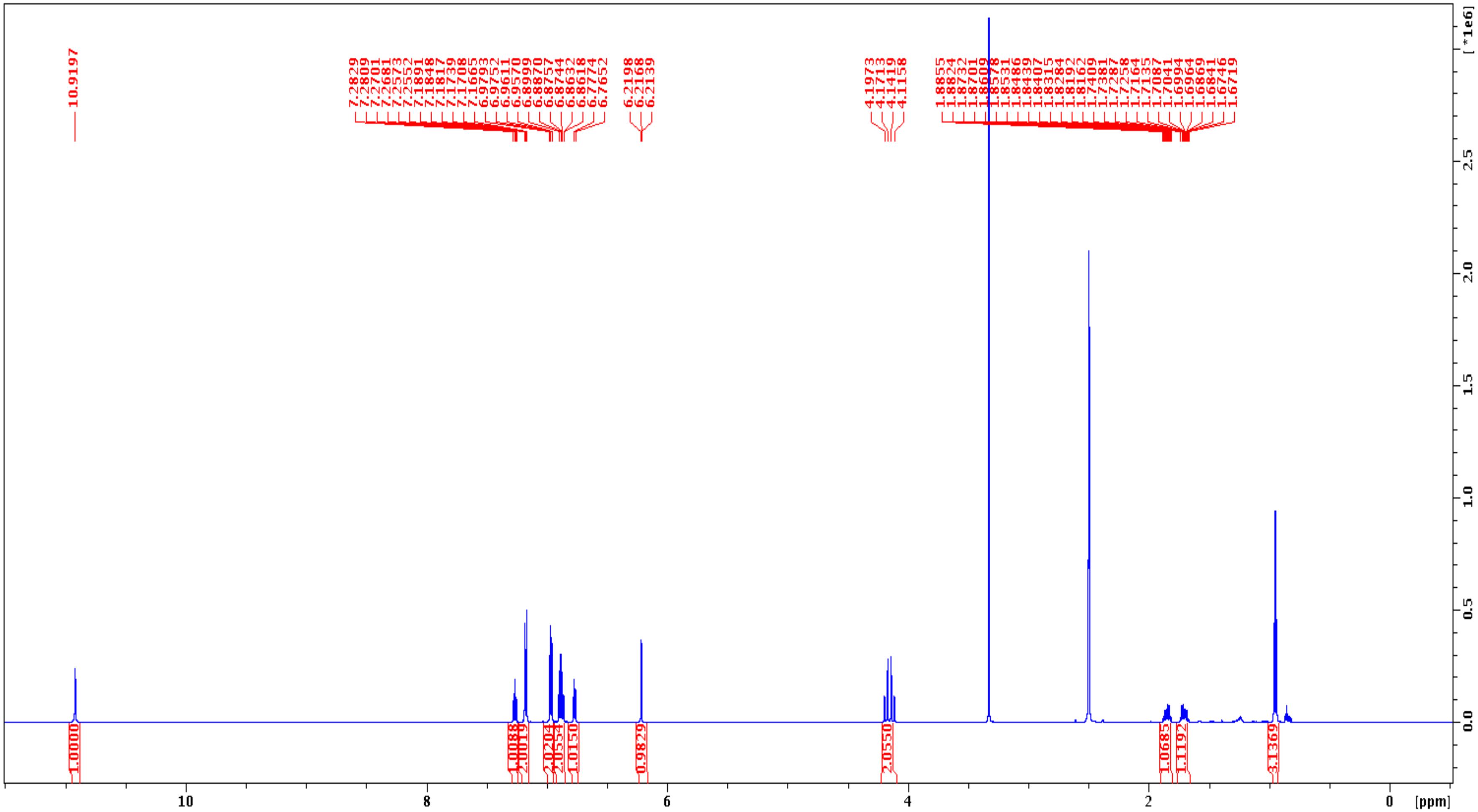

^{1}H NMR spectrum of compound **7i**

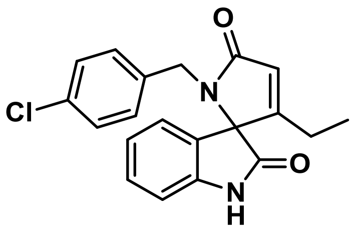

7i

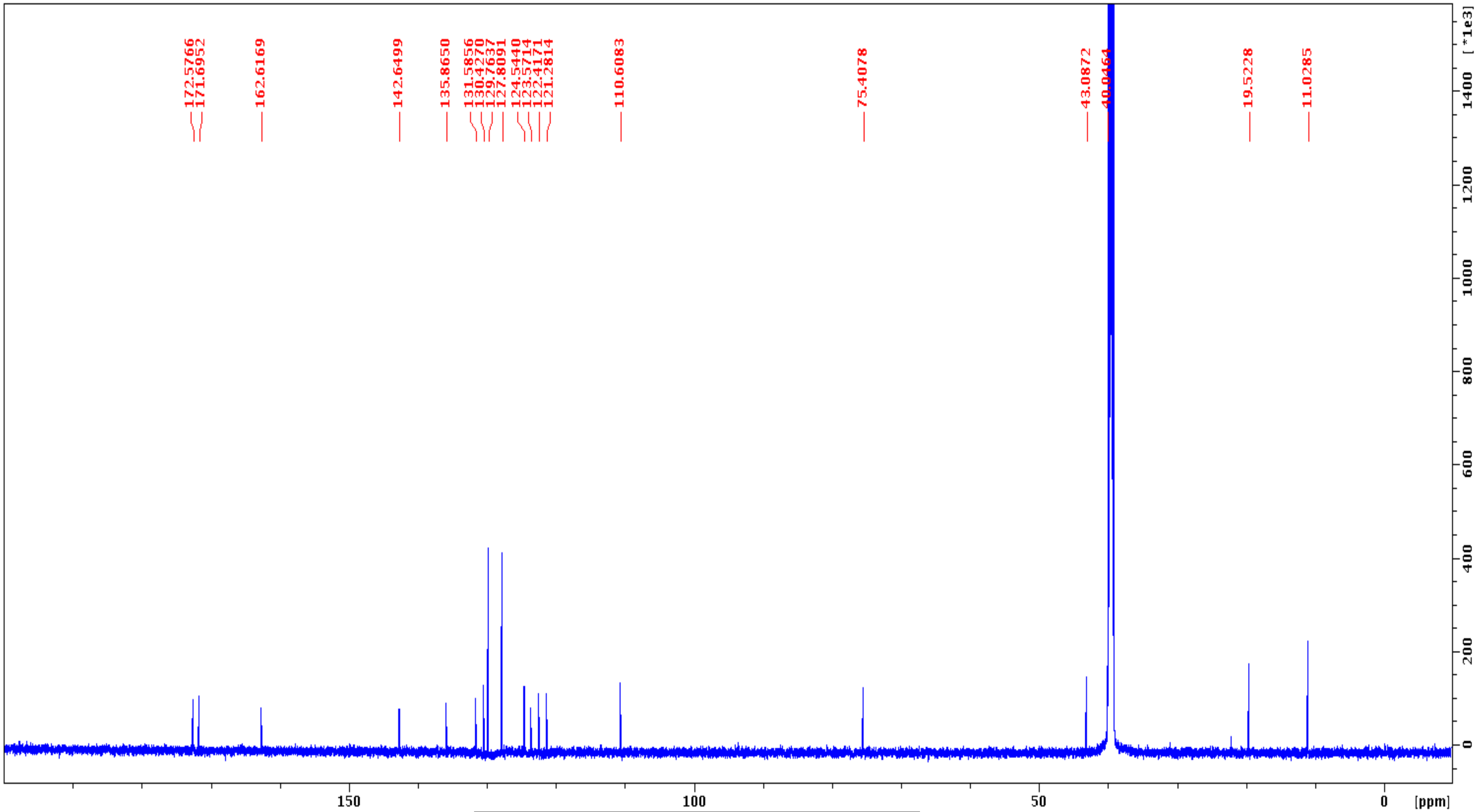

^{13}C NMR spectrum of compound **7i**

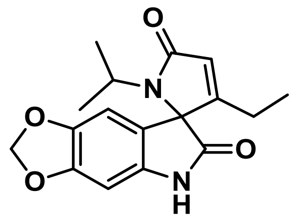

7j

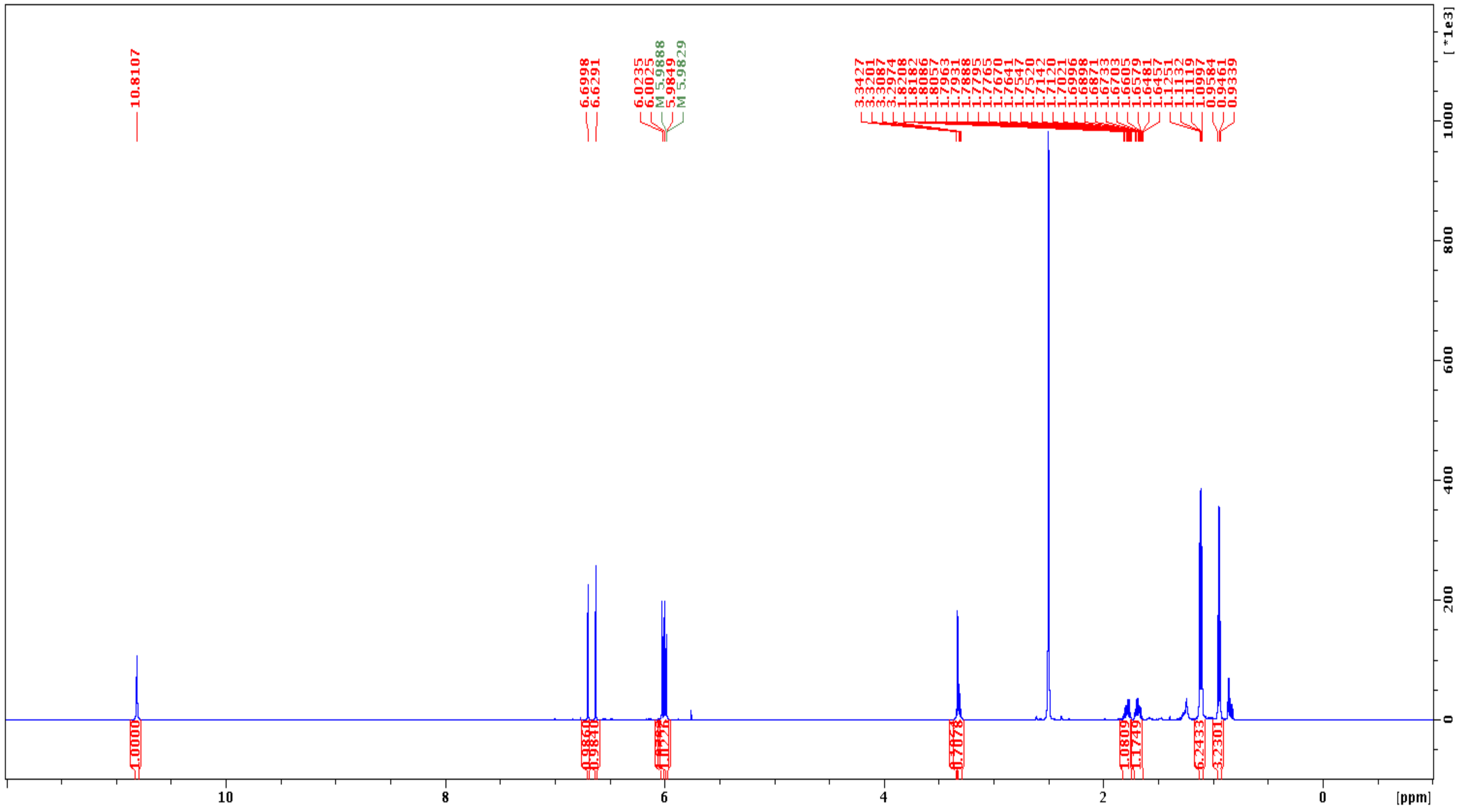

^{1}H NMR spectrum of compound **7j**

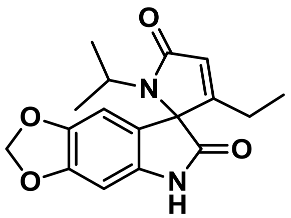

7j

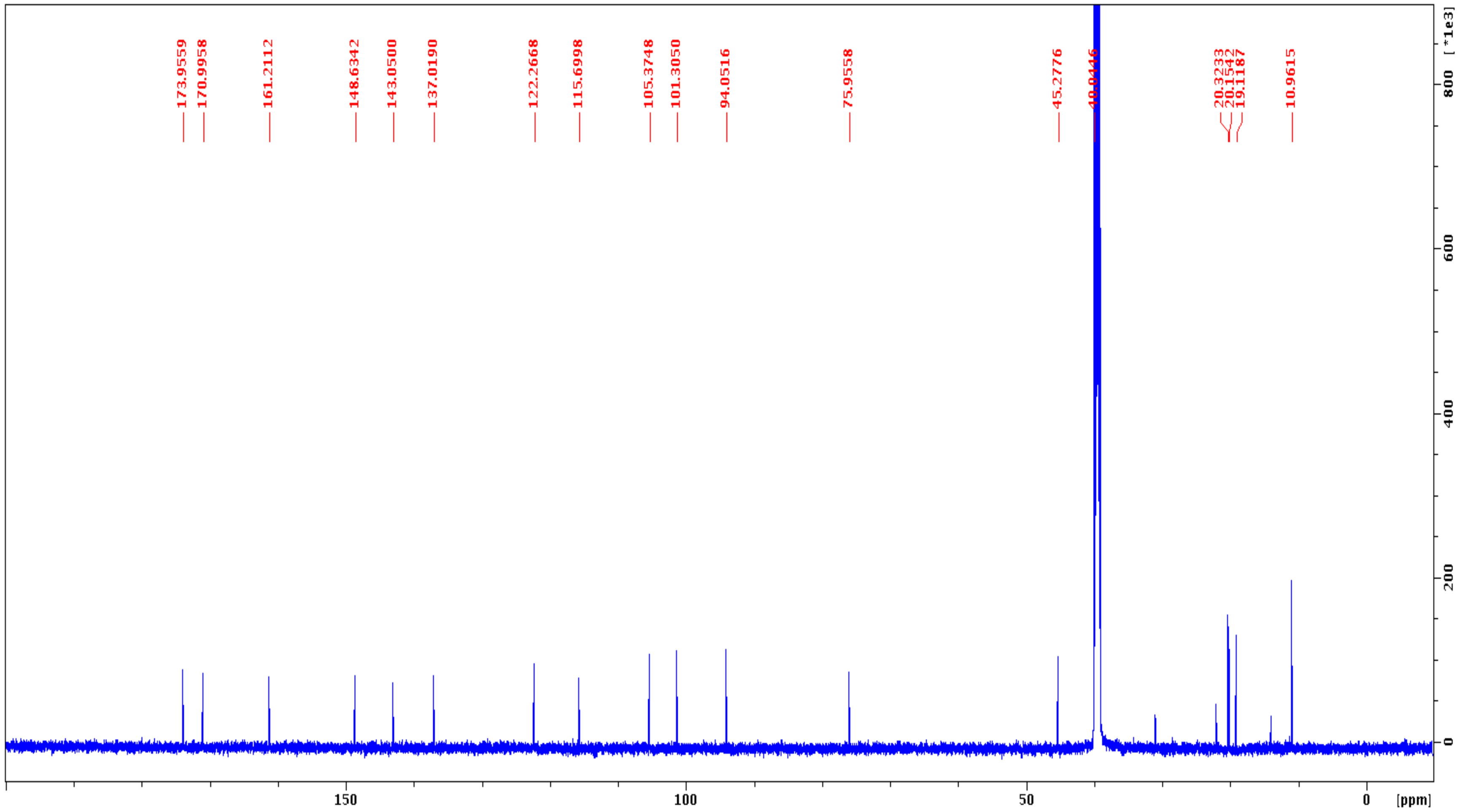

^{13}C NMR spectrum of compound **7j**

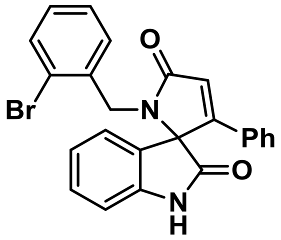

7k

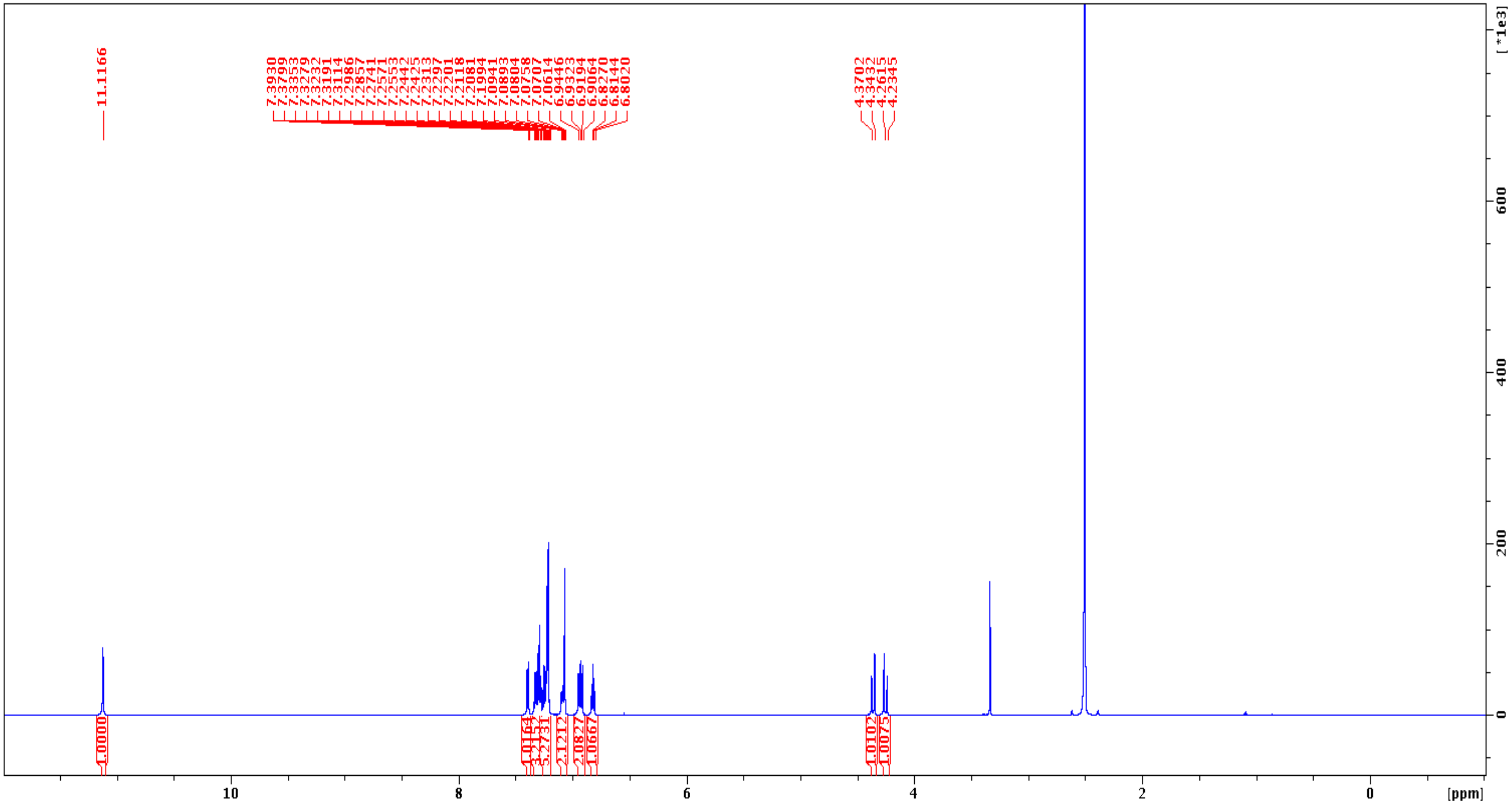

^{1}H NMR spectrum of compound **7k**

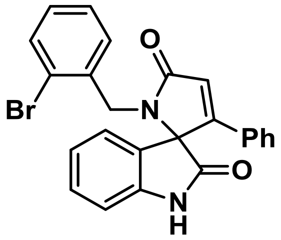

7k

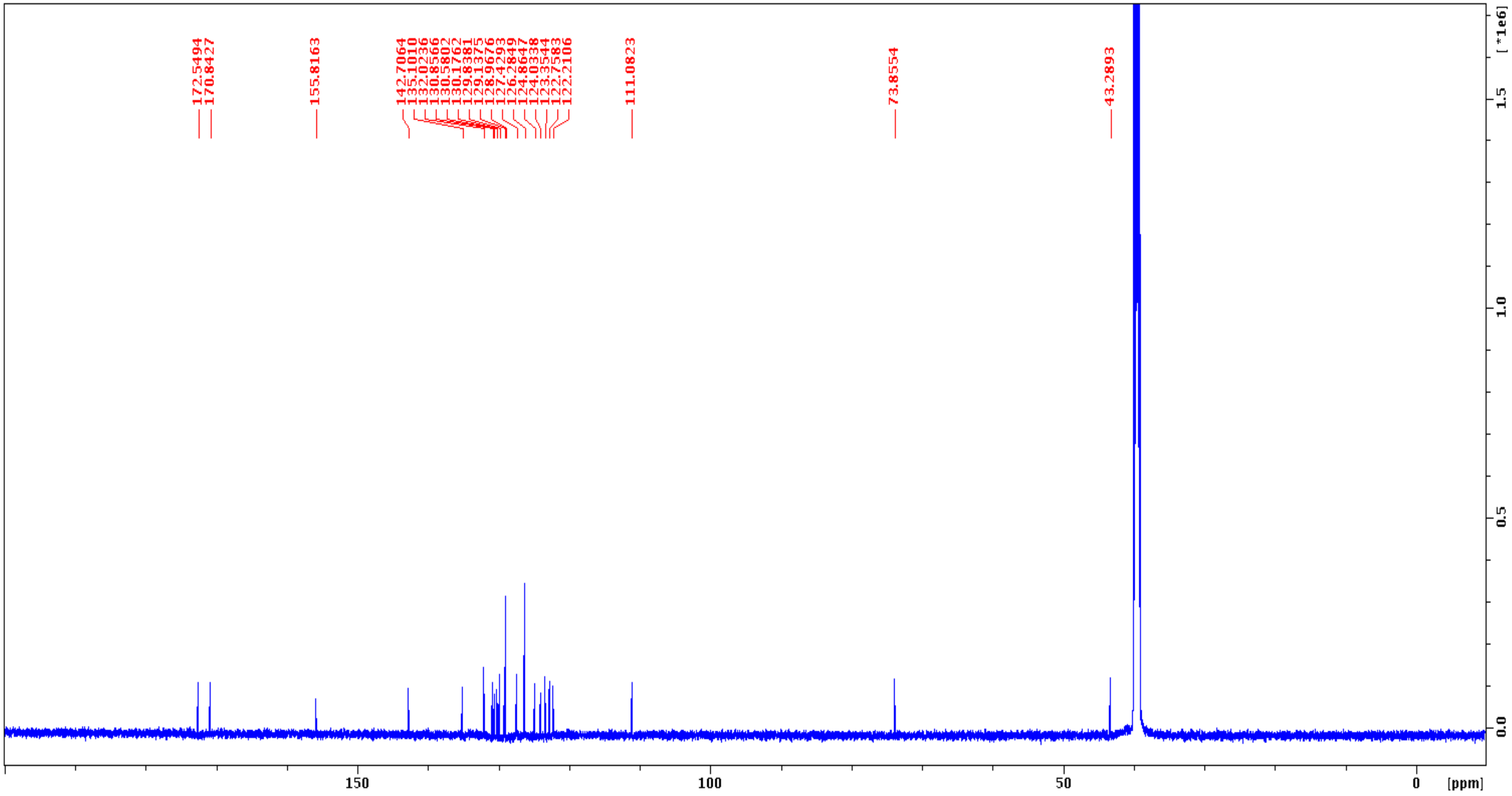

^{13}C NMR spectrum of compound **7k**

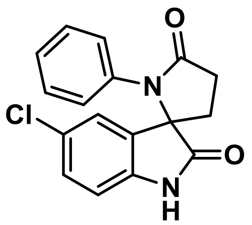

8a

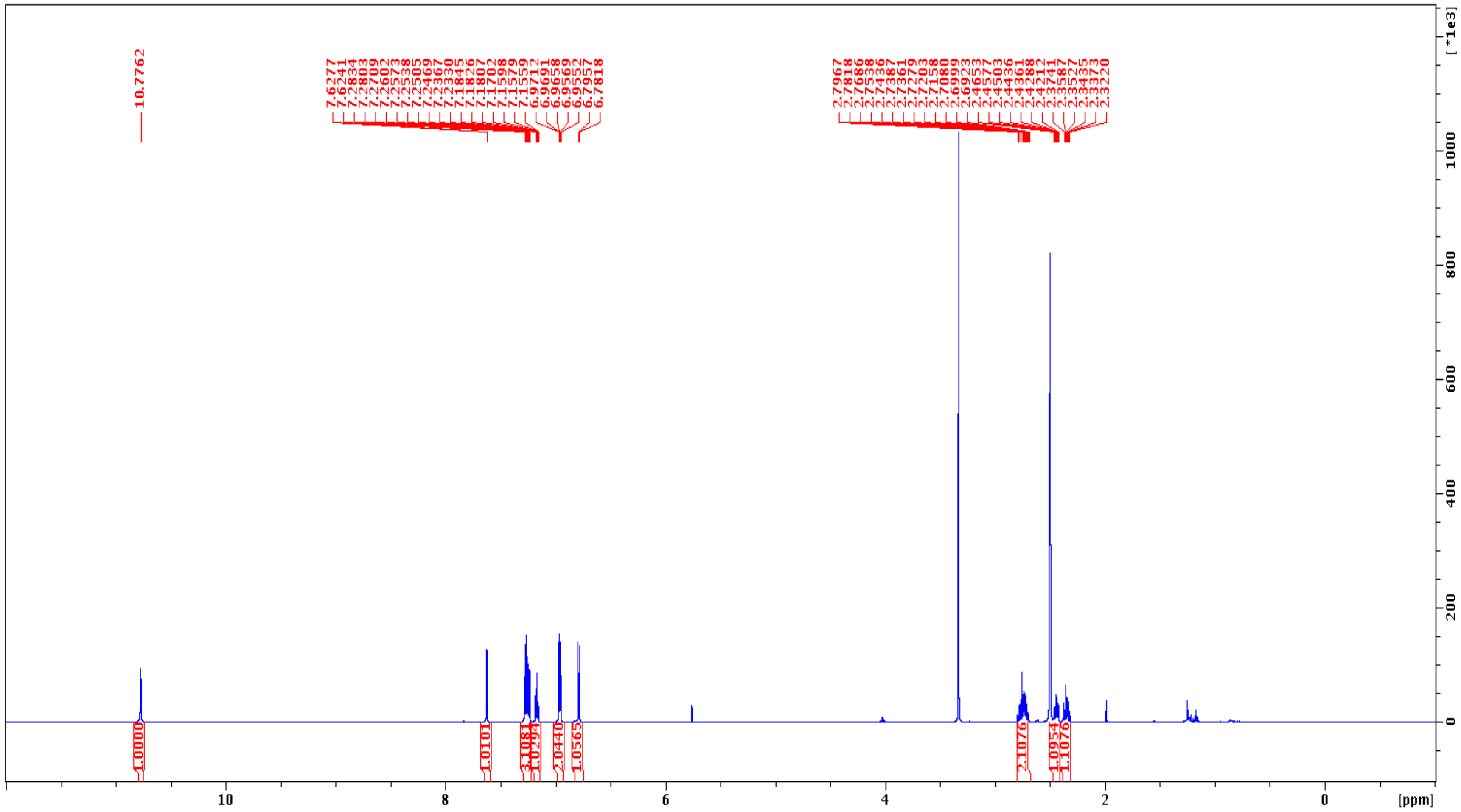

^{1}H NMR spectrum of compound **8a**

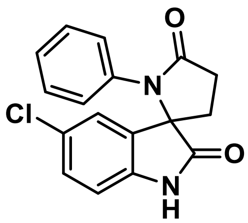

8a

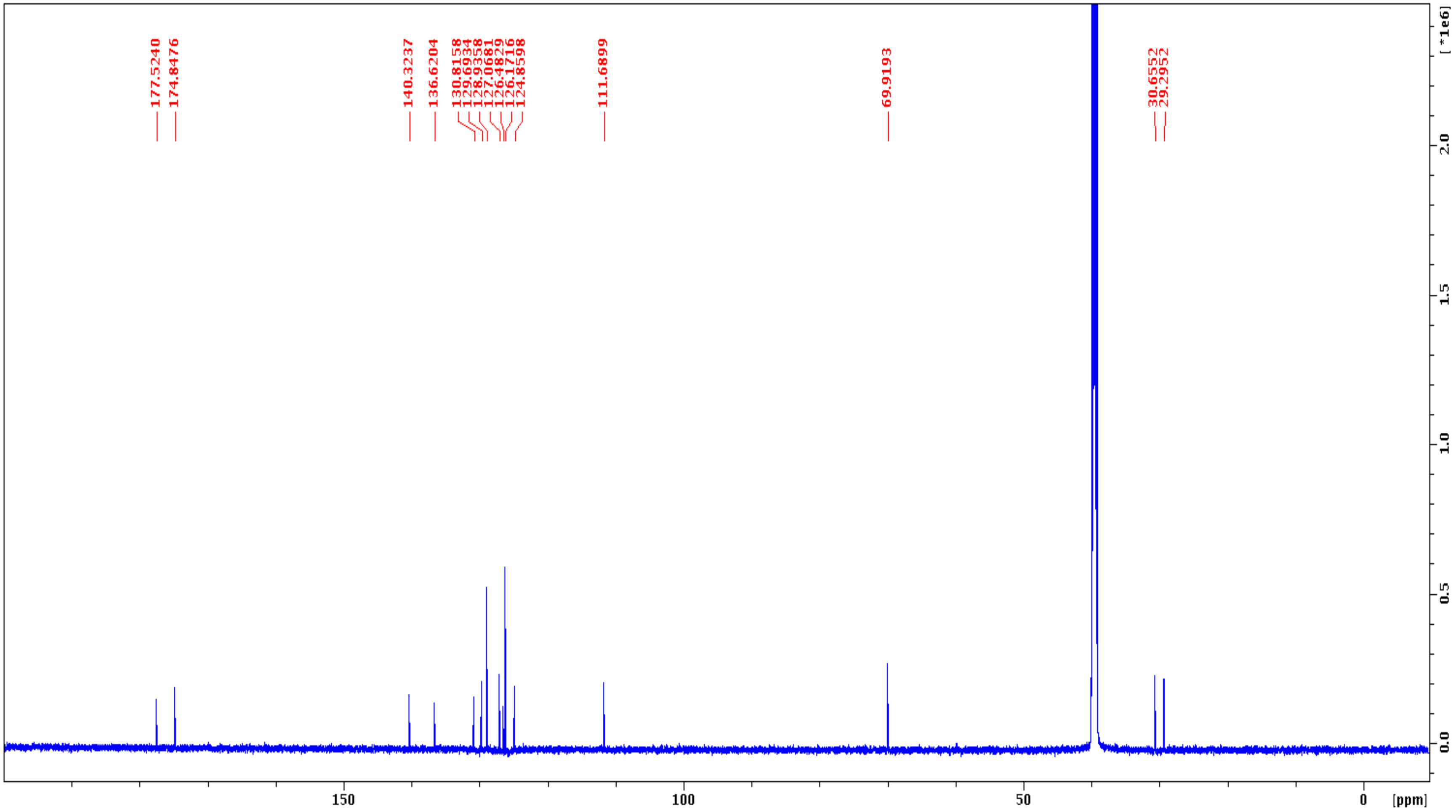

^{13}C NMR spectrum of compound **8a**

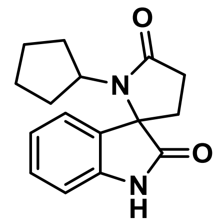

8b

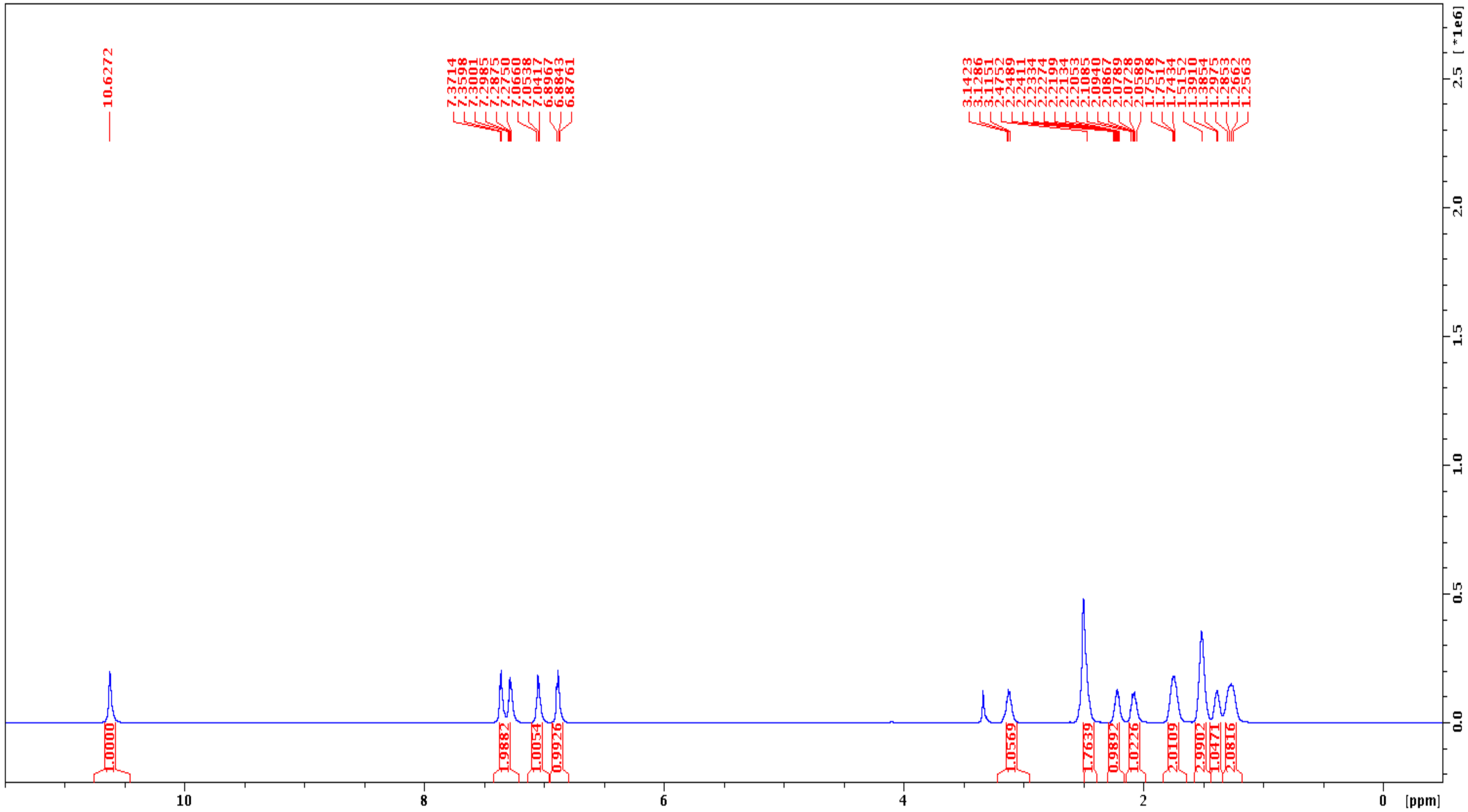

^{1}H NMR spectrum of compound **8b**

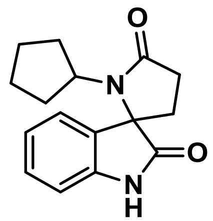

8b

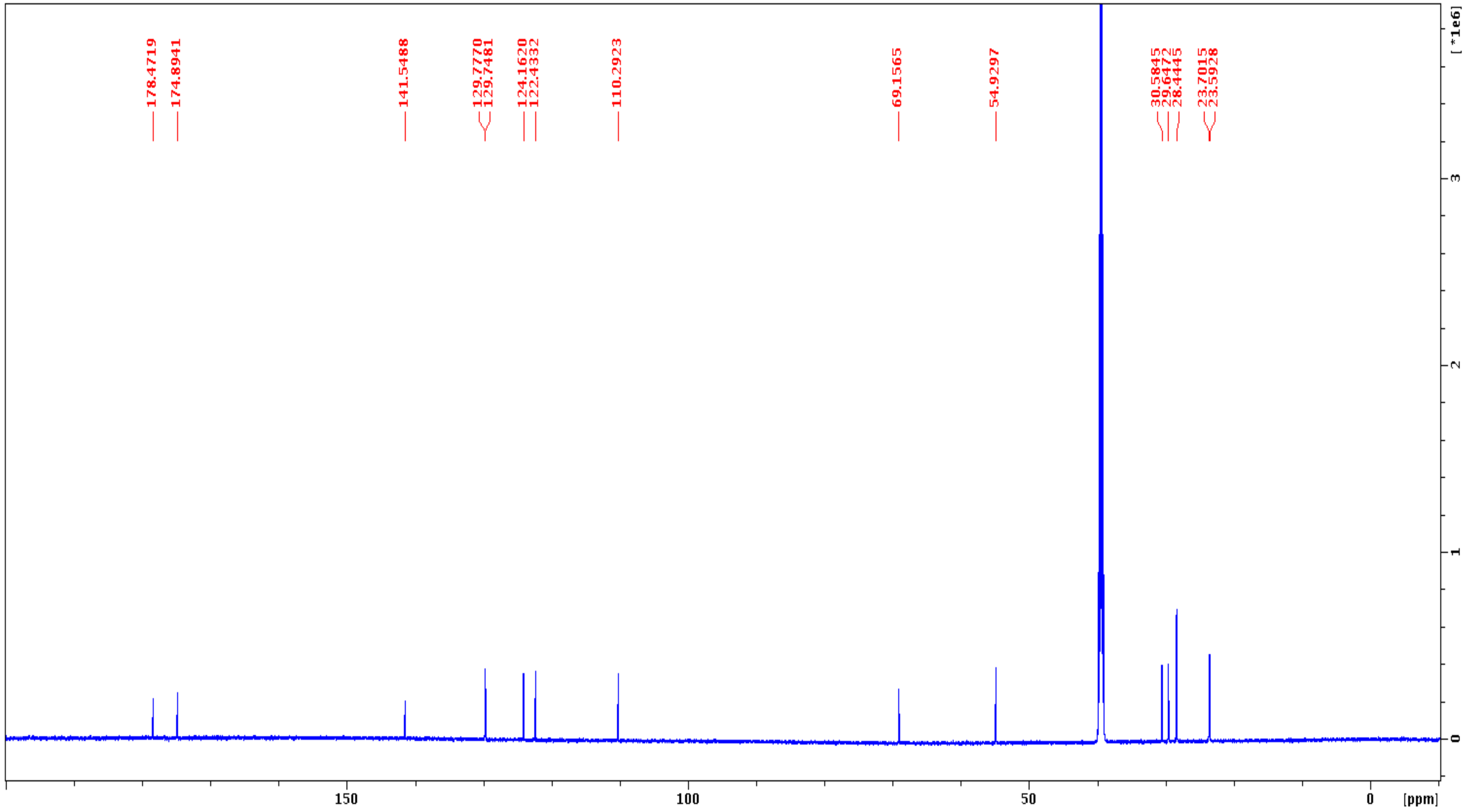

^{13}C NMR spectrum of compound **8b**

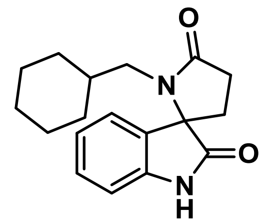

8c

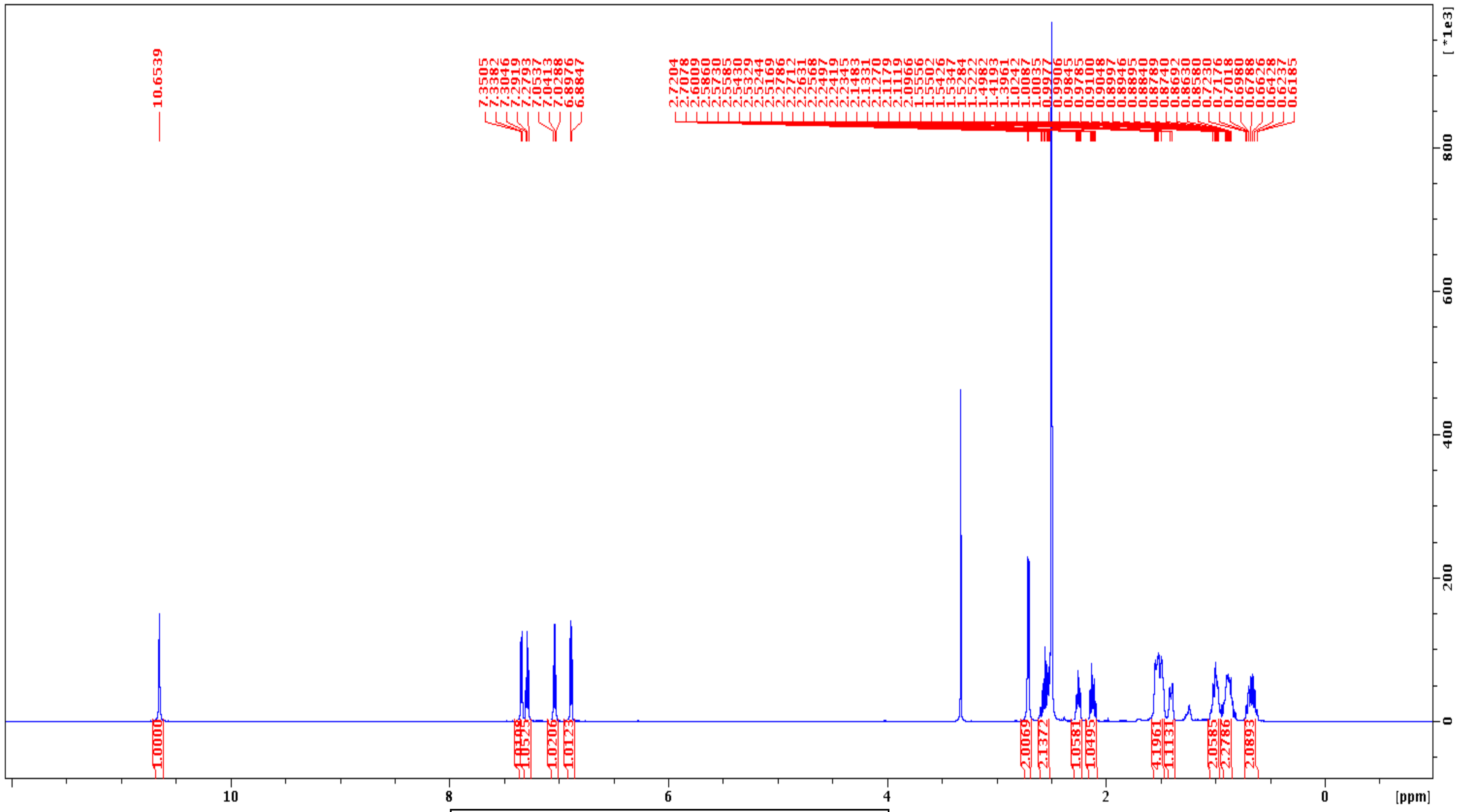

^{1}H NMR spectrum of compound **8c**

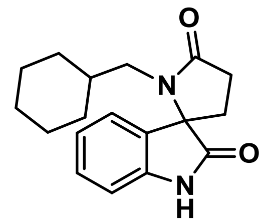

8c

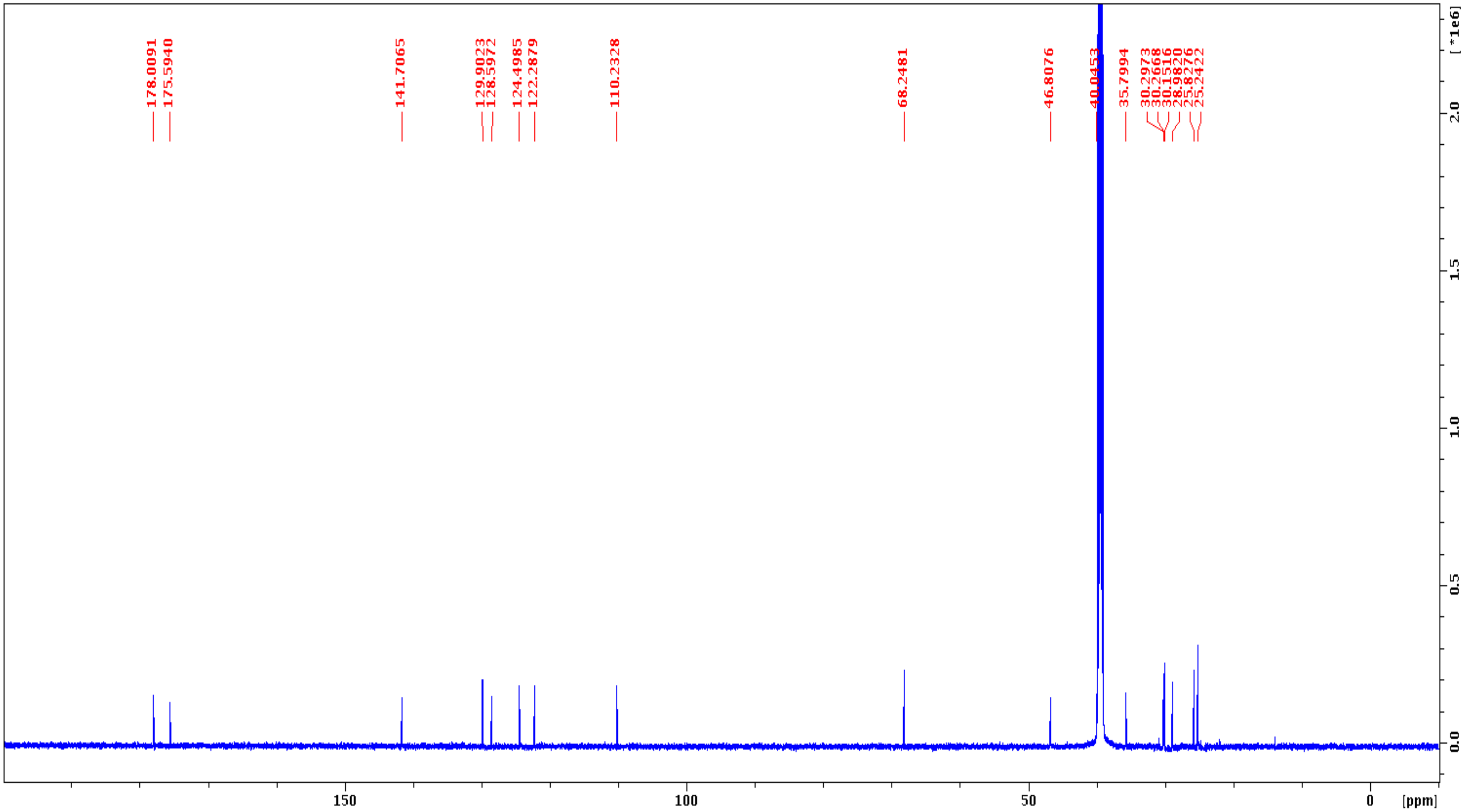

^{13}C NMR spectrum of compound **8c**

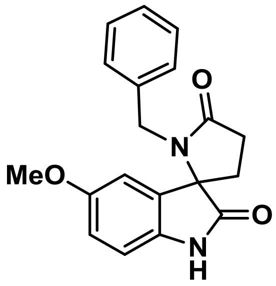

8d

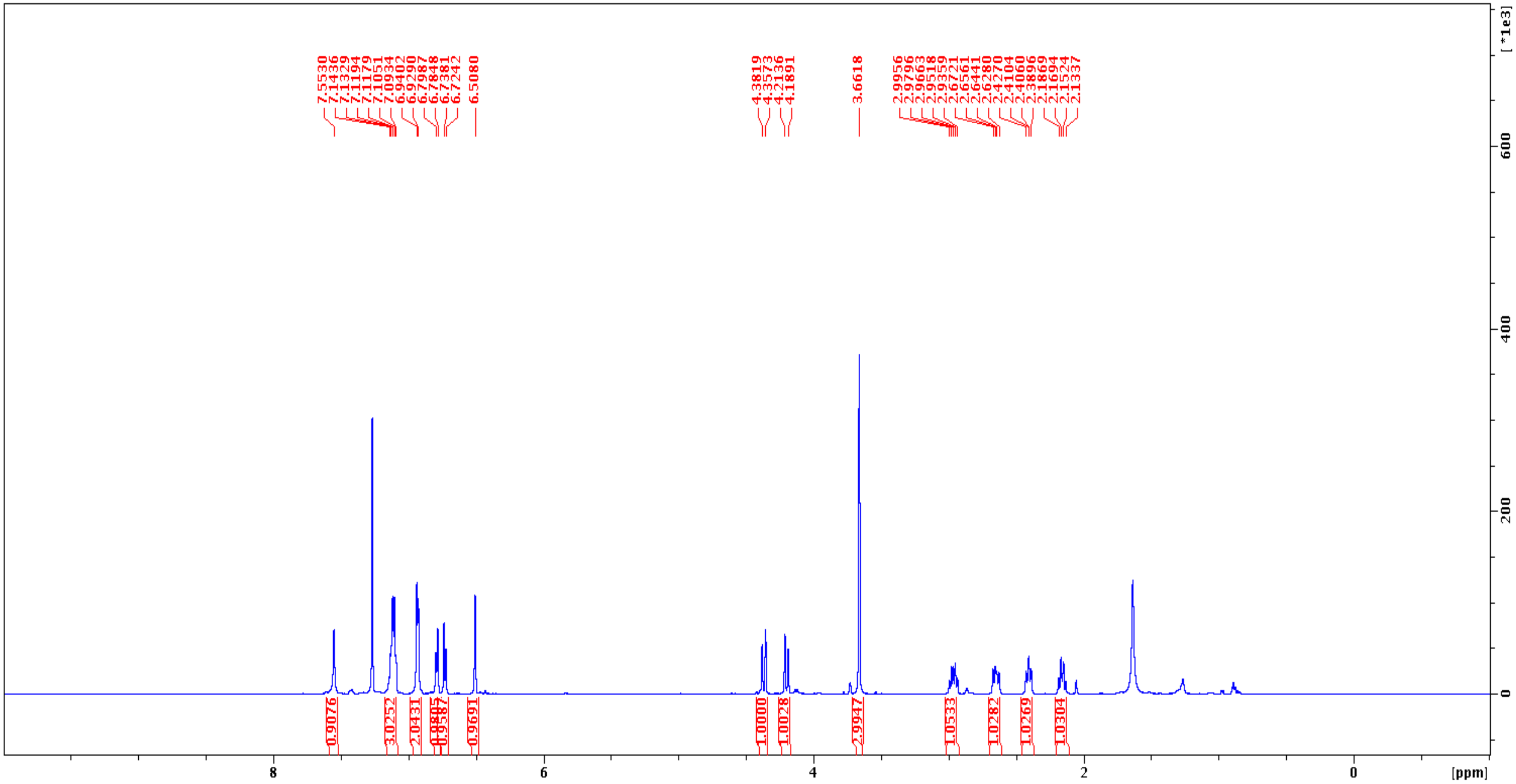

^{1}H NMR spectrum of compound **8d**

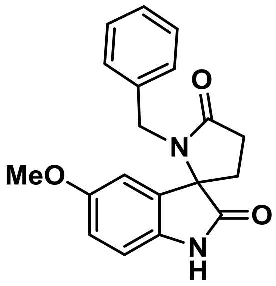

8d

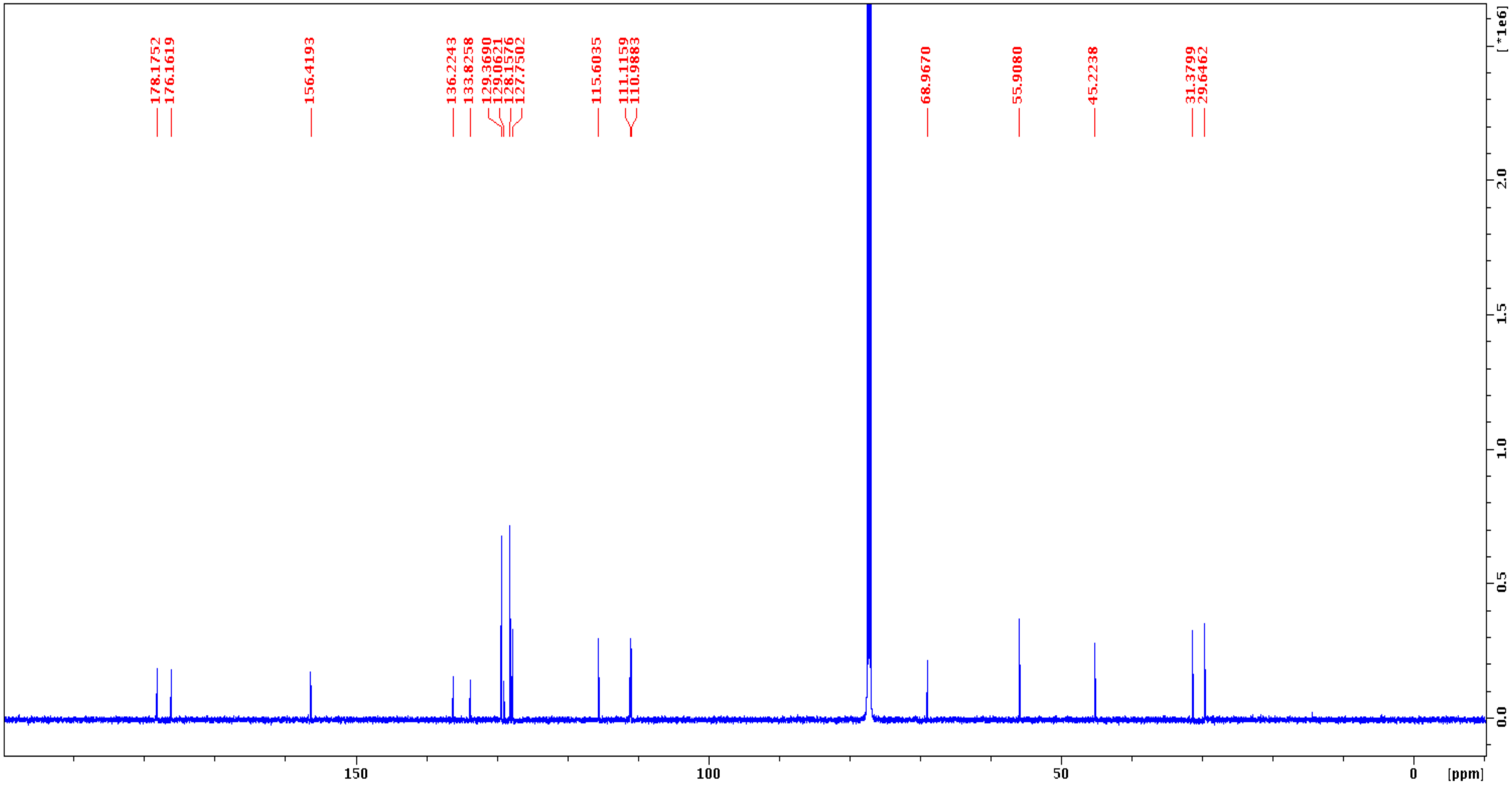

^{13}C NMR spectrum of compound **8d**

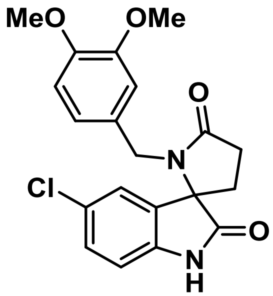

8e

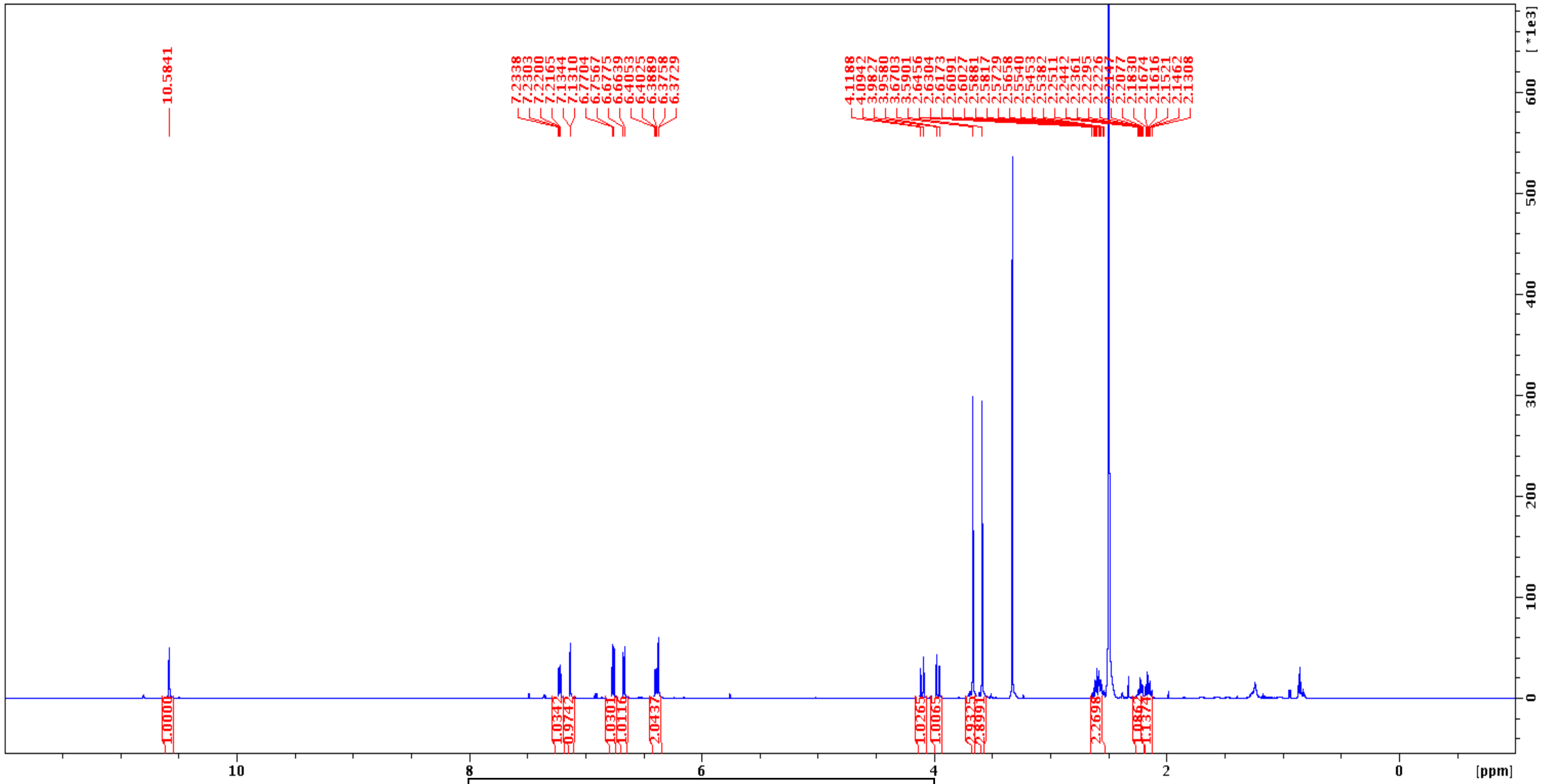

^{1}H NMR spectrum of compound **8e**

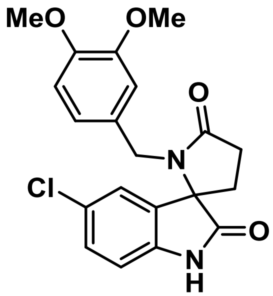

8e

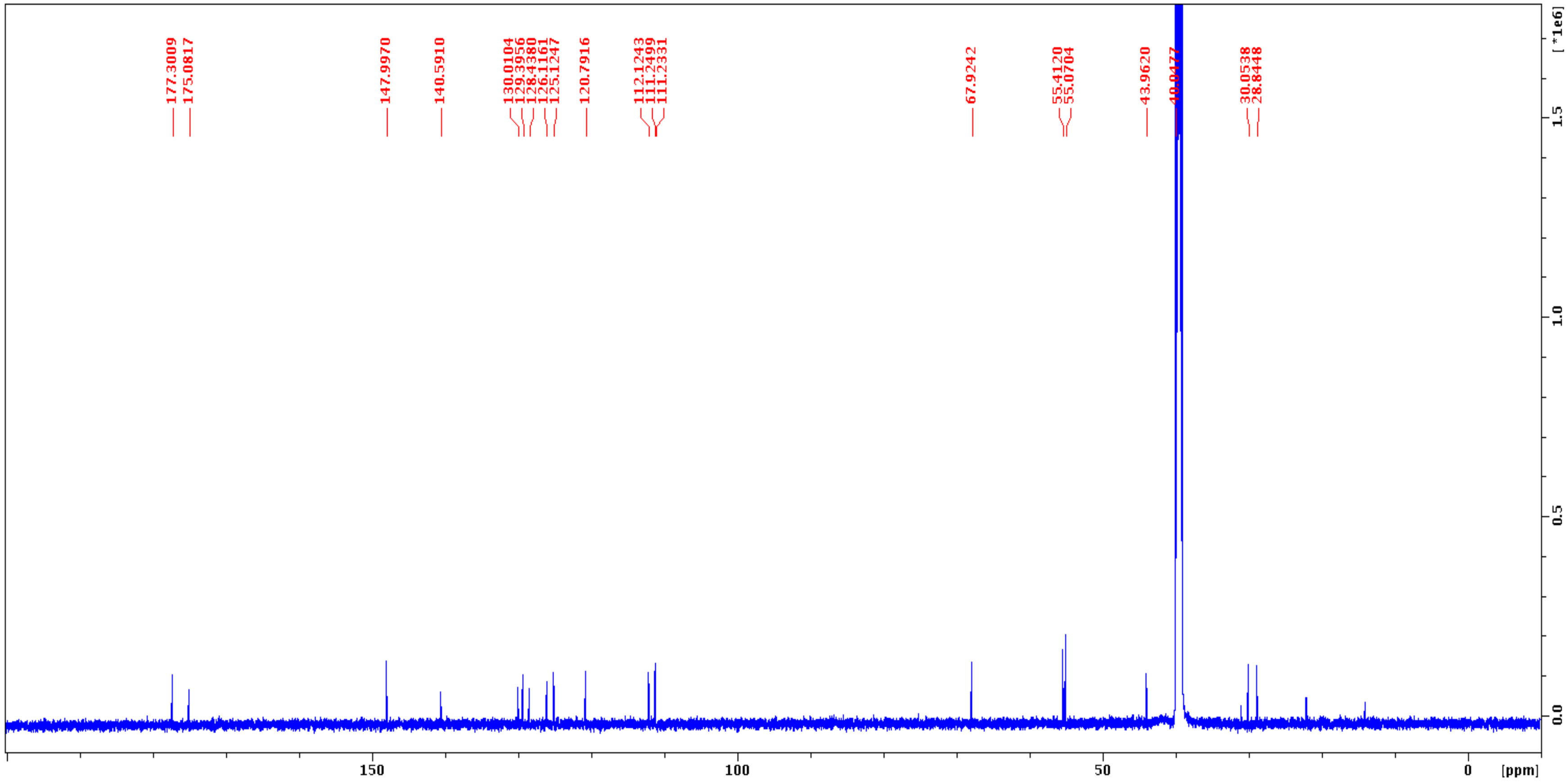

^{13}C NMR spectrum of compound **8e**

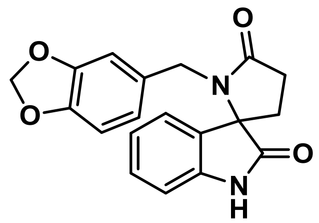

8f

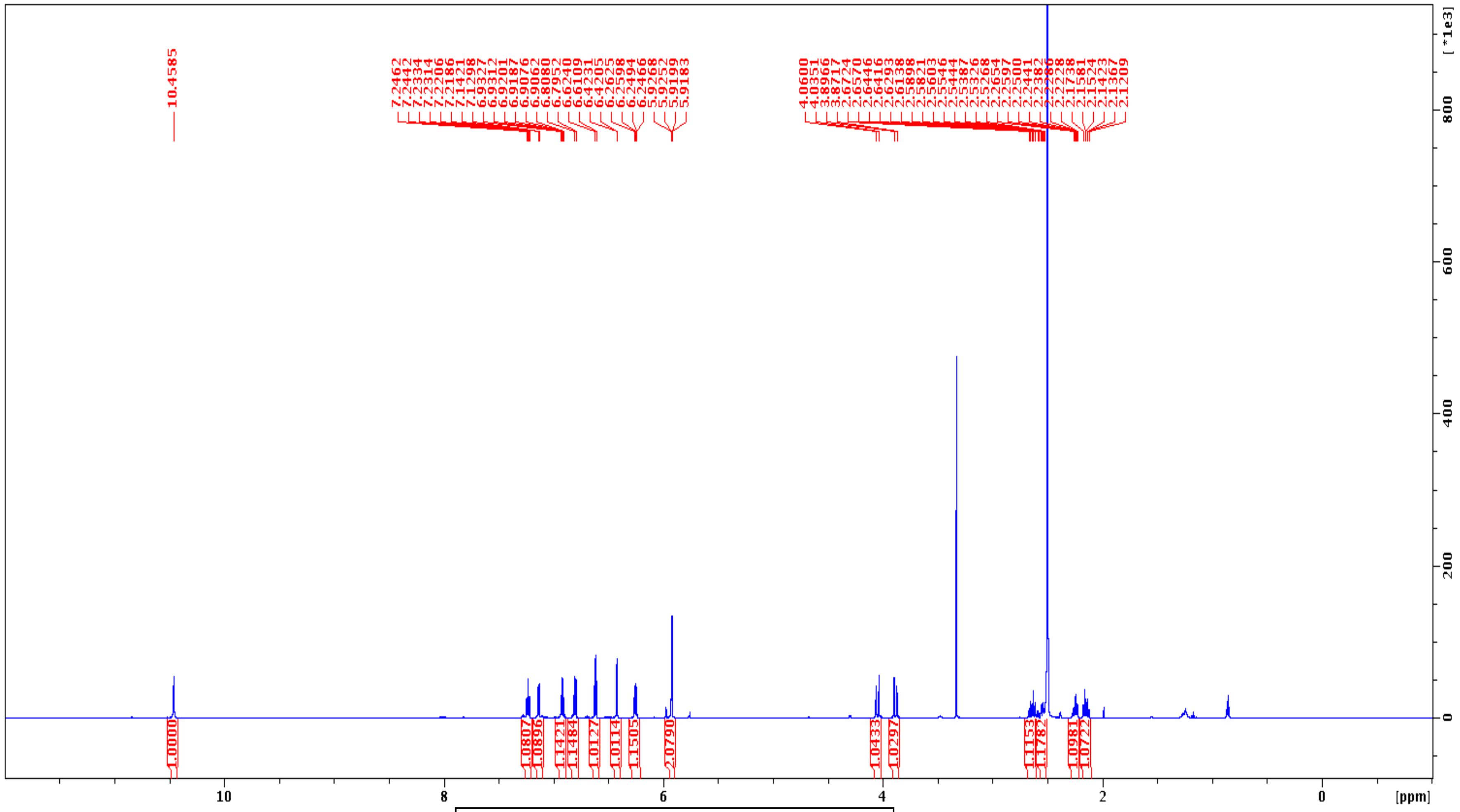

^{1}H NMR spectrum of compound **8f**

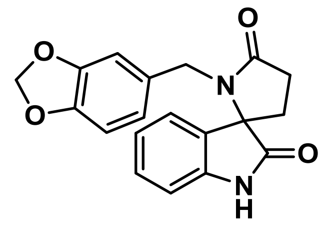

8f

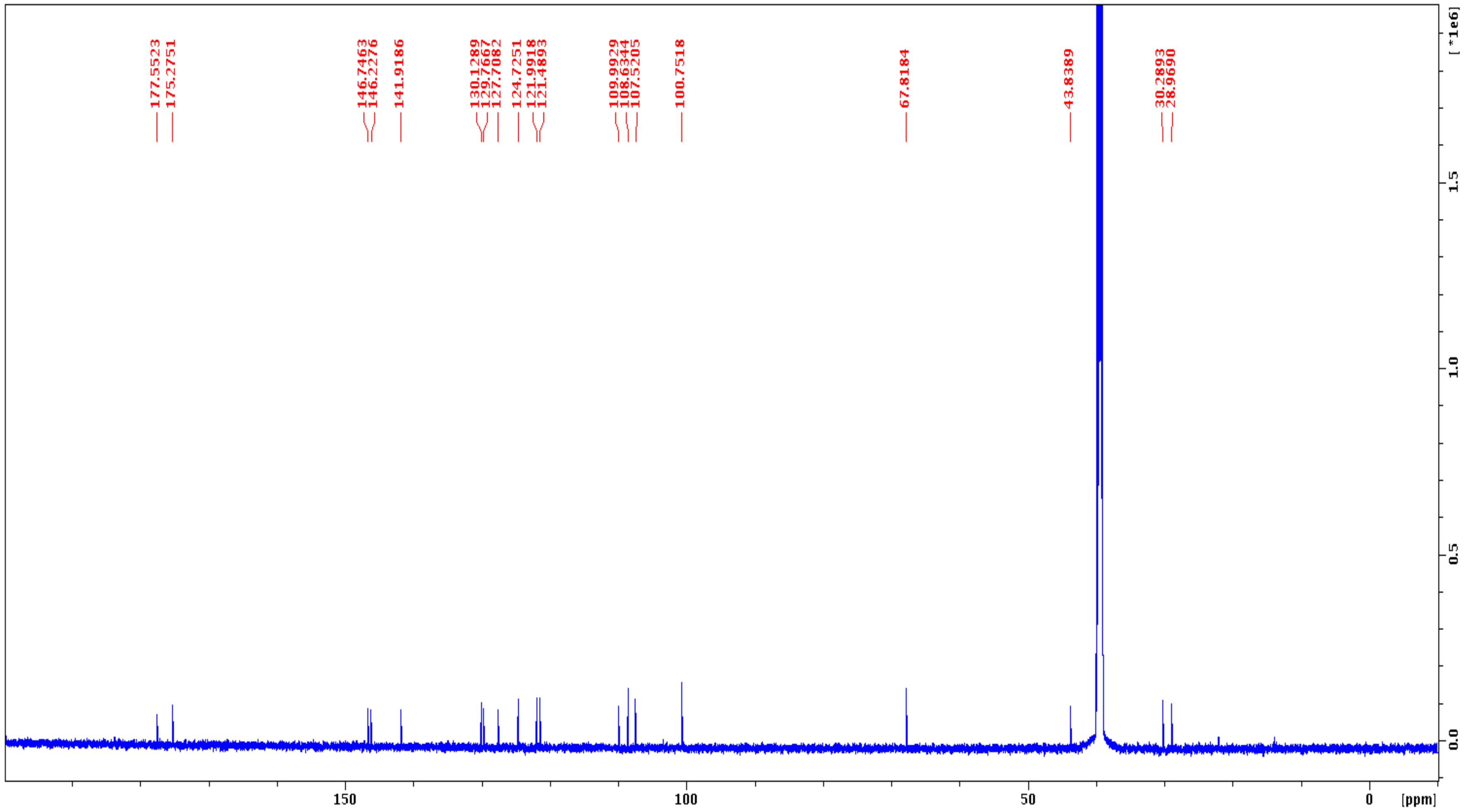

^{13}C NMR spectrum of compound **8f**

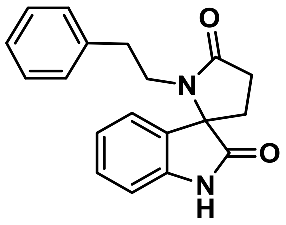

8g

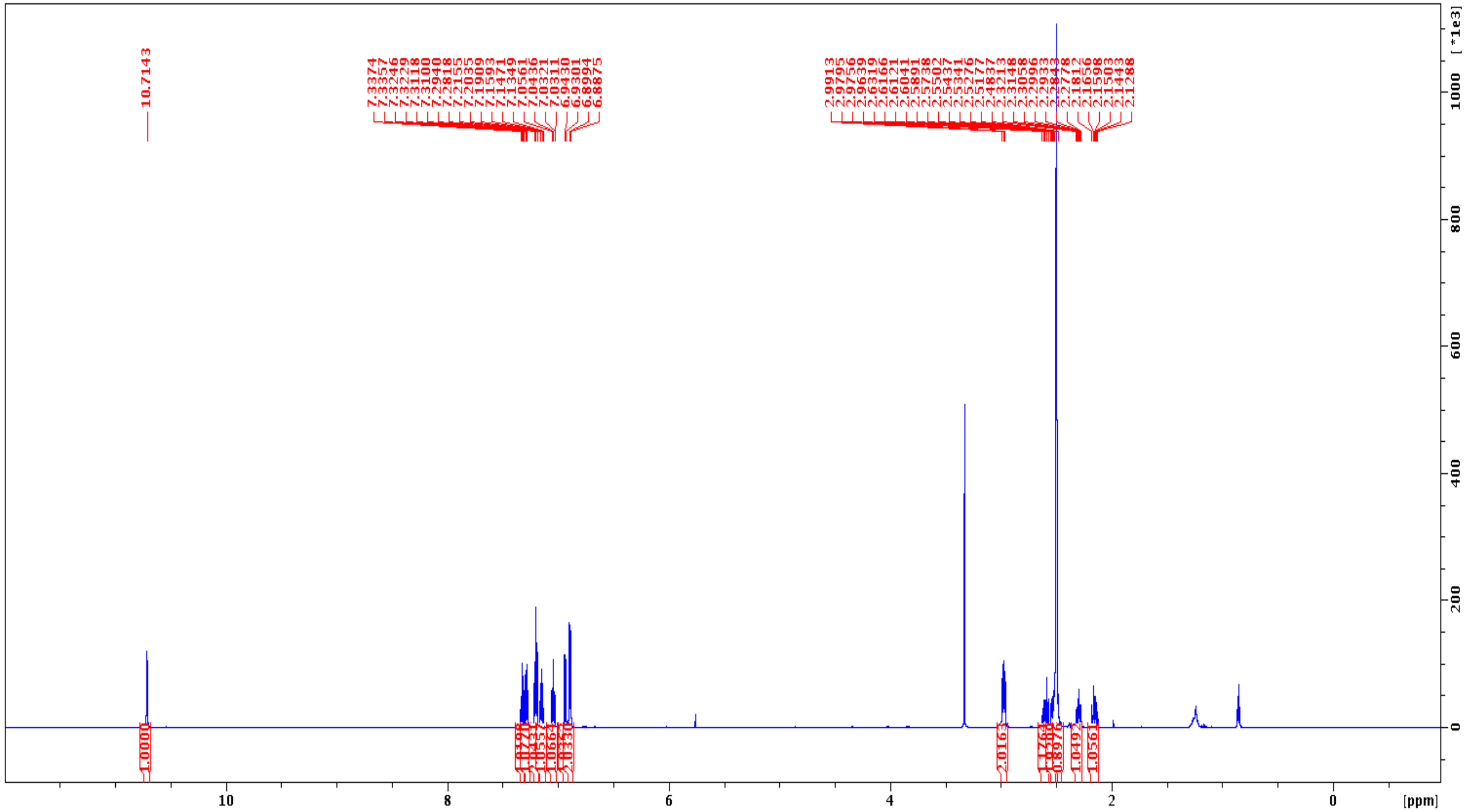

^{1}H NMR spectrum of compound **8g**

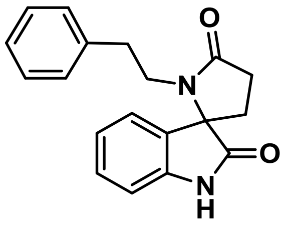

8g

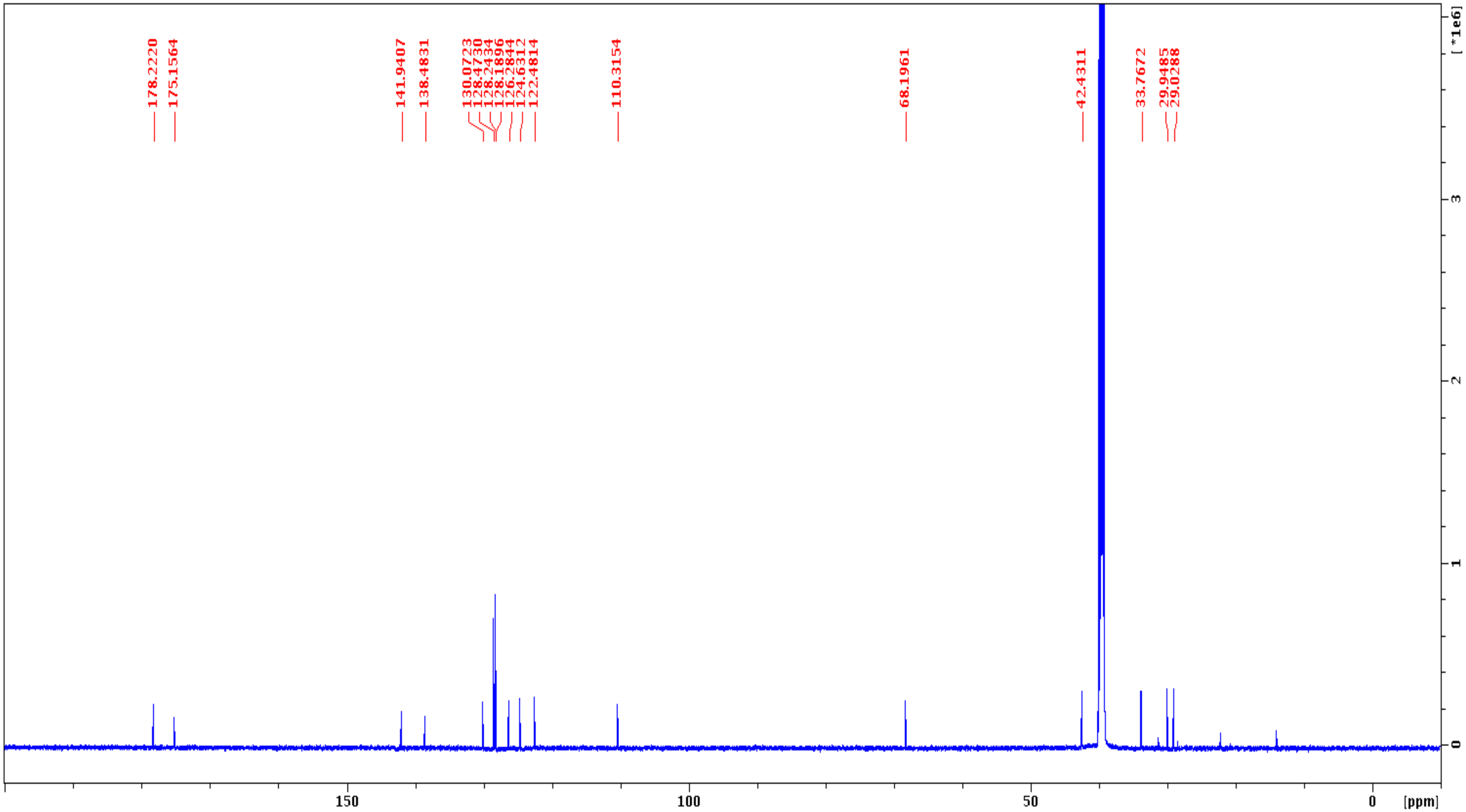

^{13}C NMR spectrum of compound **8g**

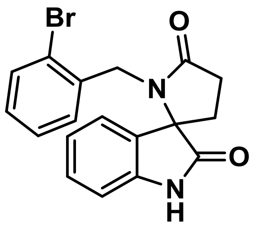

8h

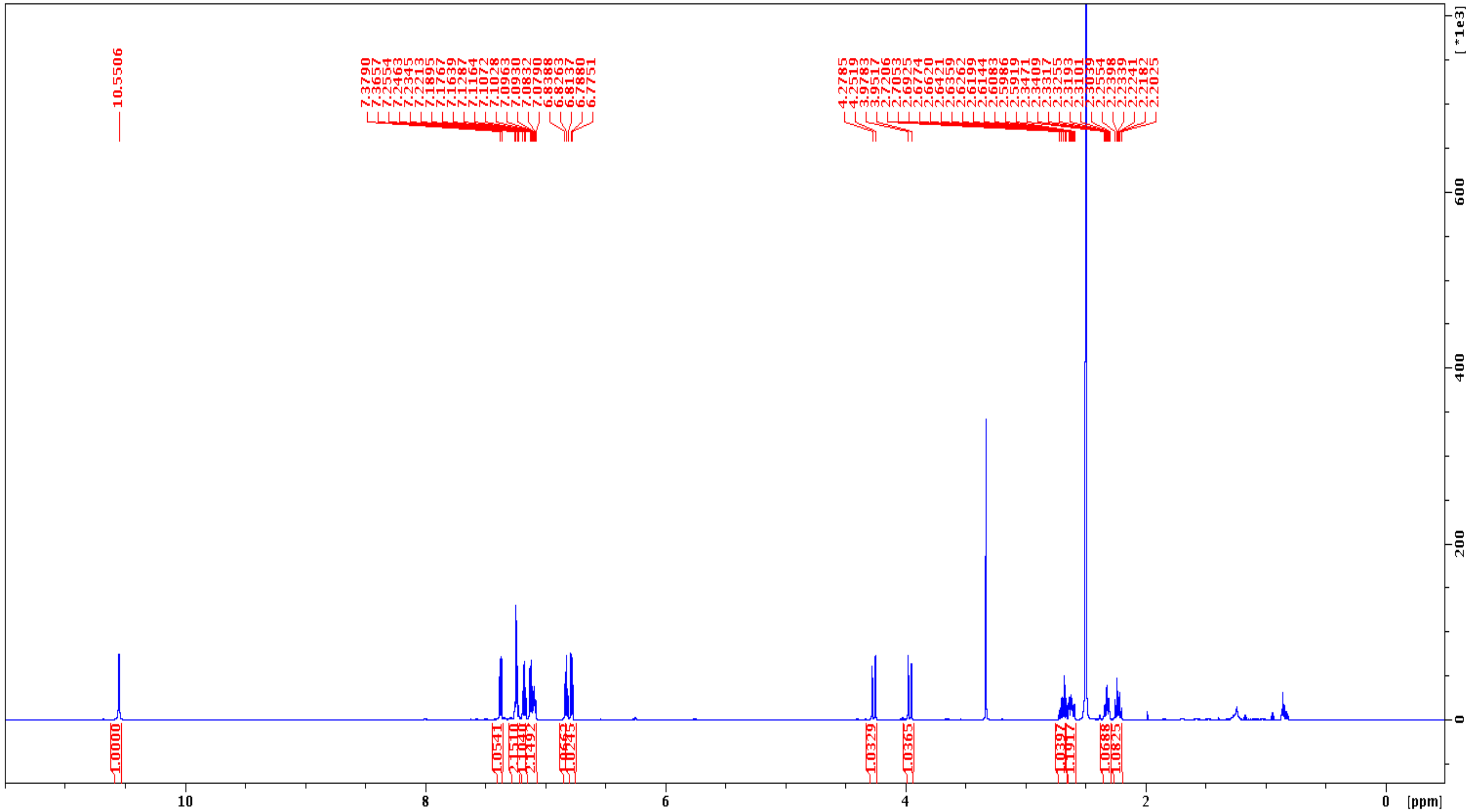

^{1}H NMR spectrum of compound **8h**

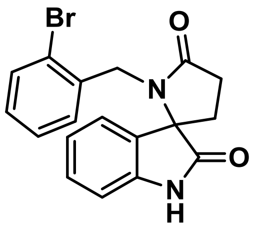

8h

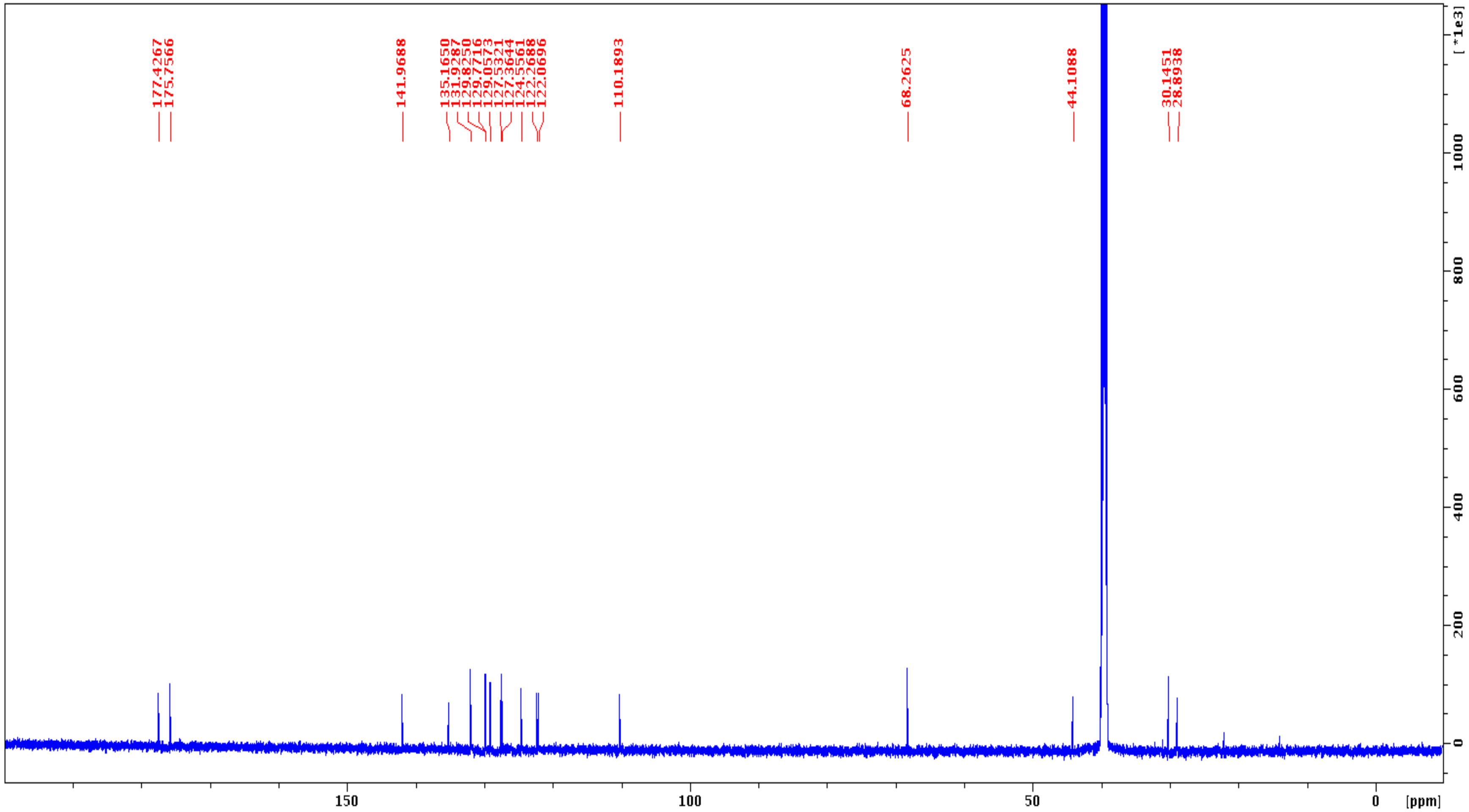

^{13}C NMR spectrum of compound **8h**

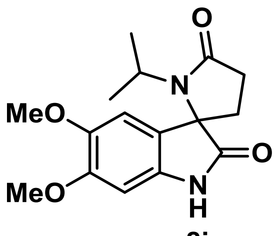

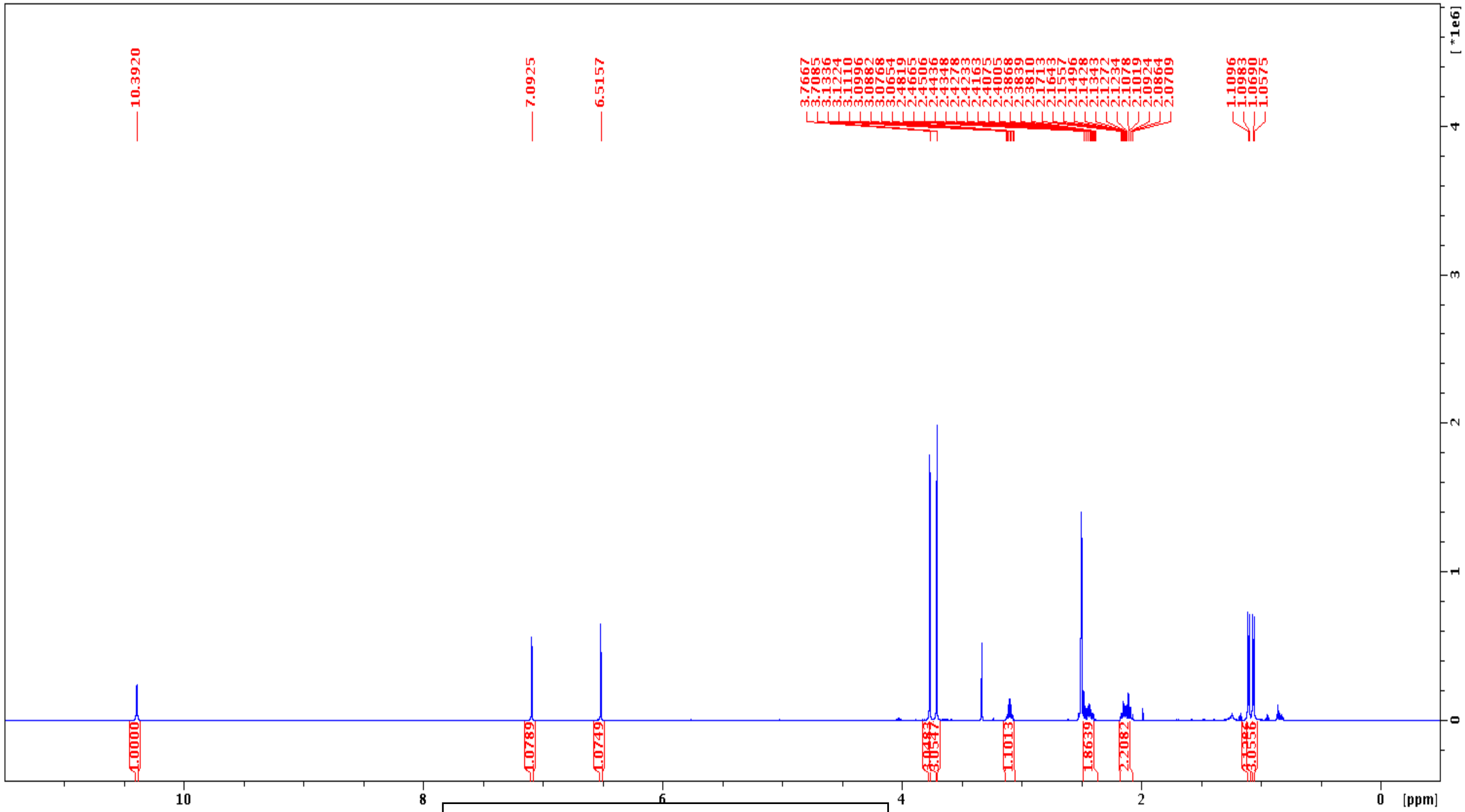

^{1}H NMR spectrum of compound **8i**

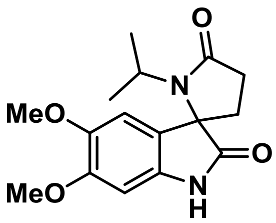

8i

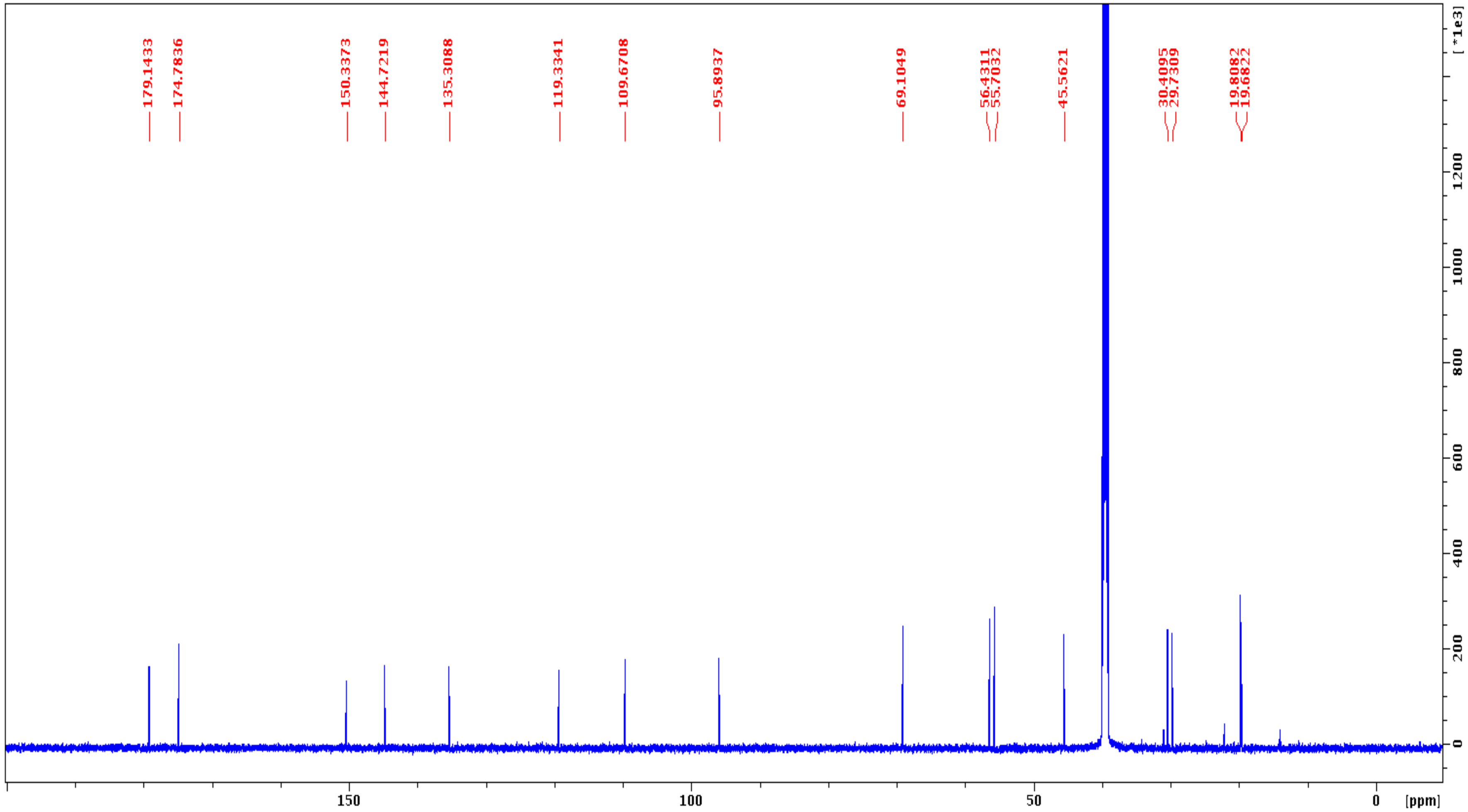

^{13}C NMR spectrum of compound **8i**

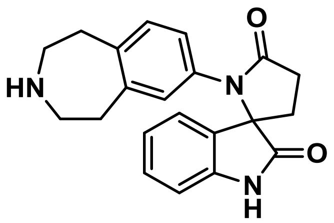

8j

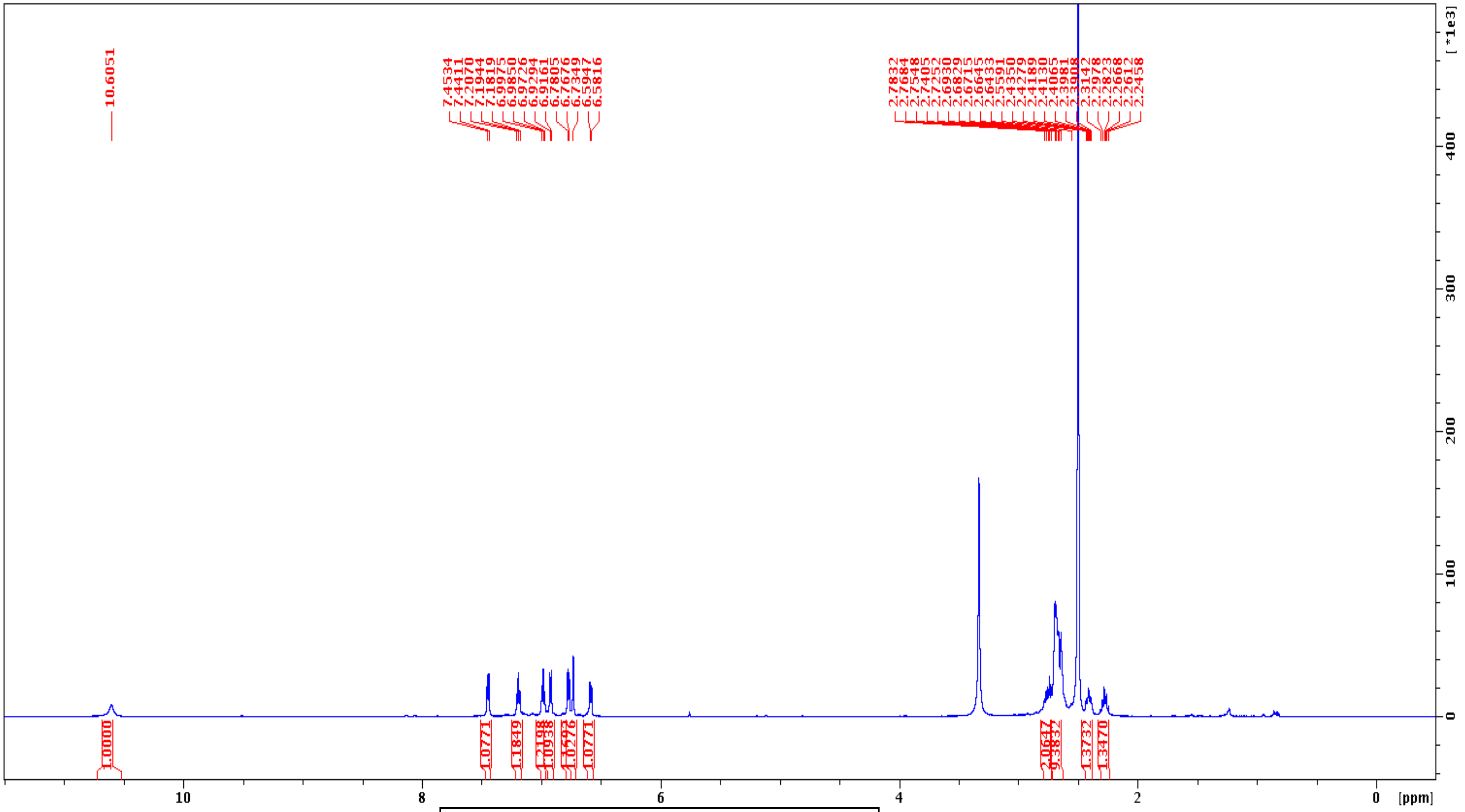

^{1}H NMR spectrum of compound **8j**

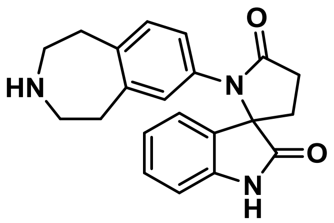

8j

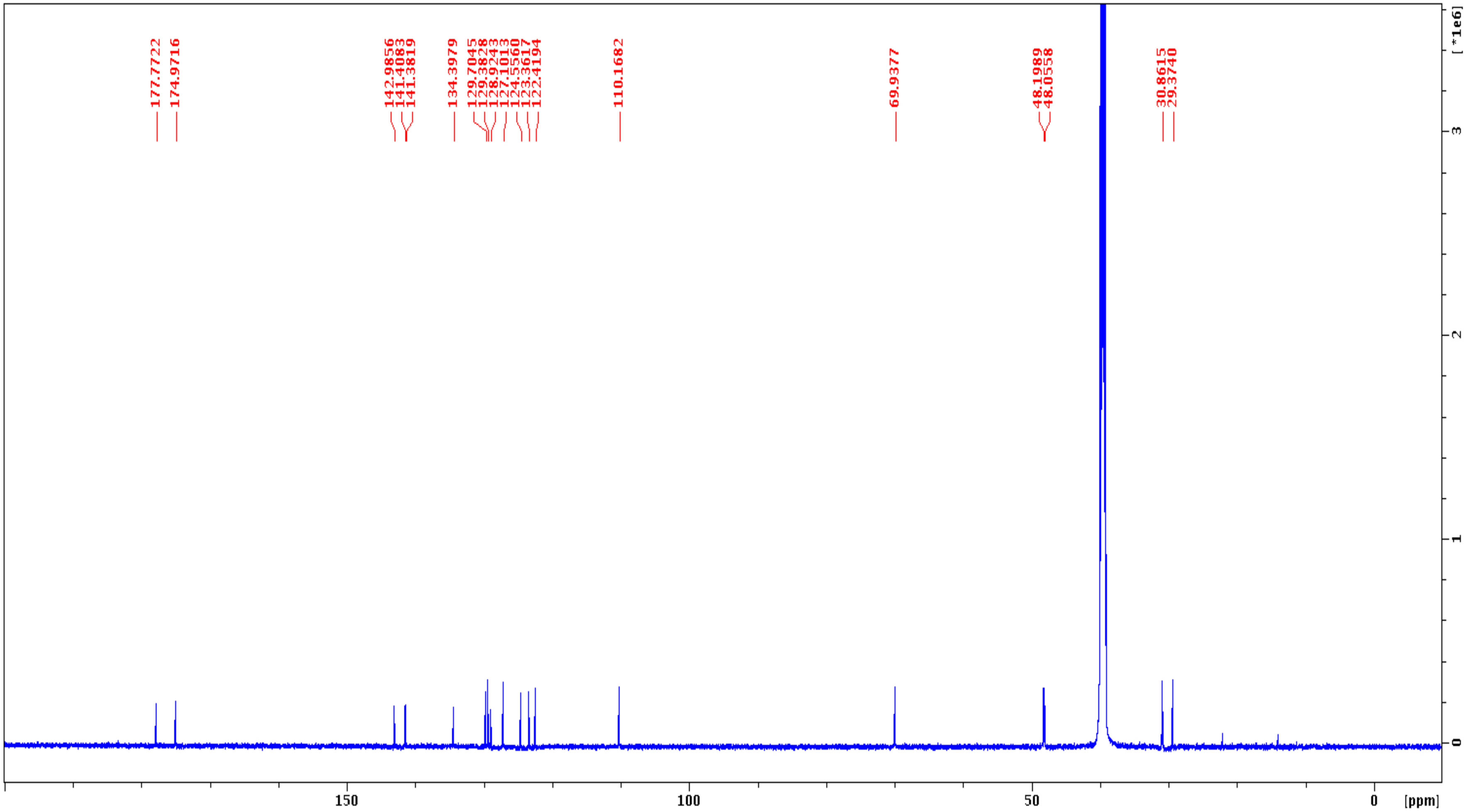

^{13}C NMR spectrum of compound **8j**

X-ray crystal structure details for 3'-methyl-1'-phenylspiro[indoline-3,2'-pyrrole]-2,5'(1'*H*)-dione (7a).

A specimen of $C_{18}H_{14}N_2O_2$ was used for the X-ray crystallographic analysis. The X-ray intensity data were measured. The integration of the data using a monoclinic unit cell yielded a total of 23672 reflections to a maximum θ angle of 33.03° (0.65 Å resolution), of which 4886 were independent (average redundancy 4.845, completeness = 95.9%, R_{int} = 2.10%, R_{sig} = 1.47%) and 4423 (90.52%) were greater than 2σ (F^2). The final cell constants of a = 8.610(2) Å, b = 15.226(4) Å, c = 10.416(2) Å, β = 98.327(3)°, volume = 1351.1(5) $Å^3$, are based upon the refinement of the XYZ-centroids of 9908 reflections above 20 σ(I) with 4.772° < 2θ < 65.99°. Data were corrected for absorption effects using the Multi-Scan method (SADABS). The ratio of minimum to maximum apparent transmission was 0.914. The structure was solved and refined using the Bruker SHELXTL Software Package, using the space group P 1 21/n 1, with Z = 4 for the formula unit, $C_{18}H_{14}N_2O_2$. The final anisotropic full-matrix least-squares refinement on F^2 with 255 variables converged at R1 = 3.63%, for the observed data and wR2 = 10.25% for all data. The goodness-of-fit was 1.079. The largest peak in the final difference electron density synthesis was 0.529 e/$Å^3$ and the largest hole was −0.226 e/$Å^3$ with an RMS deviation of 0.055 e/$Å^3$. On the basis of the final model, the calculated density was 1.427 g/cm^3 and F(000), 608 e.

Sample and crystal data for compound 7a

**Chemical formula**	$C_{18}H_{14}N_2O_2$
**Formula weight**	290.31 g/mol
**Temperature**	100(2) K

**Wavelength**	0.71073 Å	
**Crystal system**	monoclinic	
**Space group**	*P*121/*n*1	
**Unit cell dimensions**	*a* = 8.610(2) Å	α = 90°
	b = 15.226(4) Å	β = 98.327(3)°
	c = 10.416(2) Å	γ = 90°
**Volume**	1351.1(5) $Å^3$	
**Z**	4	
**Density (calculated)**	1.427 g/cm^3	
**Absorption coefficient**	0.095 mm^{-1}	
**F(000)**	608	

Data collection and structure refinement for compound 7a.

**Theta range for data collection**	2.39 to 33.03°
**Index ranges**	-12<=h<=13, -23<=k<=22, -15<=l<=15
**Reflections collected**	23672
**Independent reflections**	4886 [R(int) = 0.0210]
**Coverage of independent reflections**	95.9%
**Absorption correction**	Multi-Scan
**Structure solution technique**	direct methods
**Structure solution program**	XT, VERSION 2014/4

**Refinement method**	Full-matrix least-squares on F^2
**Refinement program**	SHELXL-2014/7 (Sheldrick, 2014)
**Function minimized**	$\Sigma\ w(F_o^2 - F_c^2)^2$
**Data / restraints / parameters**	4886 / 0 / 255
**Goodness-of-fit on F^2**	1.079
**Δ/σ_{max}**	0.001
**Final R indices**	4423 data; $I>2\sigma(I)$ R1 = 0.0363, wR2 = 0.0986
	all data R1 = 0.0405, wR2 = 0.1025
**Weighting scheme**	$w=1/[\sigma^2(F_o^2)+(0.0590P)^2+0.3554P]$<br>where $P=(F_o^2+2F_c^2)/3$
**Largest diff. peak and hole**	0.529 and -0.226 eÅ^{-3}
**R.M.S. deviation from mean**	0.055 eÅ^{-3}

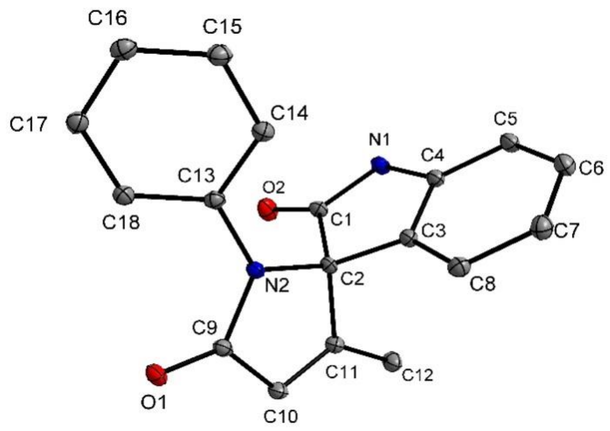

ORTEP diagram for compound **7a**.
